# Safety and Efficacy Analysis of Targeted and Immune Combination Therapy in Advanced Melanoma—A Systematic Review and Network Meta-Analysis

**DOI:** 10.3390/ijms252312821

**Published:** 2024-11-28

**Authors:** Anna Sára Lengyel, Fanni Adél Meznerics, Noémi Ágnes Galajda, Noémi Gede, Tamás Kói, Alzahra Ahmed Mohammed, Petra Nikolett Péter, Alexandra IT Lakatos, Máté Krebs, Dezső Csupor, András Bánvölgyi, Péter Hegyi, Péter Holló, Lajos V. Kemény

**Affiliations:** 1 Department of Dermatology, Venereology and Dermatooncology, Faculty of Medicine, Semmelweis University, 1085 Budapest, Hungary; 2 Centre for Translational Medicine, Semmelweis University, 1085 Budapest, Hungary; 3 HCEMM-SU Translational Dermatology Research Group, Semmelweis University, 1094 Budapest, Hungary; 4 Department of Physiology, Semmelweis University, Tűzoltó Str. 37–47, 1094 Budapest, Hungary; 5 Institute for Translational Medicine, Medical School, University of Pécs, 7623 Pécs, Hungary; 6 Department of Stochastics, Institute of Mathematics, Budapest University of Technology and Economics, 1111 Budapest, Hungary; 7 Institute of Clinical Pharmacy, Faculty of Pharmacy, University of Szeged, 6725 Szeged, Hungary; 8 Institute of Pancreatic Diseases, Semmelweis University, 1083 Budapest, Hungary; 9 MTA-SE Lendület “Momentum” Dermatooncology Research Group, 1094 Budapest, Hungary

**Keywords:** advanced melanoma, targeted therapy, immune checkpoint inhibitor, triple therapy, network meta-analysis

## Abstract

The combinations of BRAF inhibitor-based targeted therapies with immune checkpoint inhibitors currently represent less common therapeutic approaches in advanced melanoma. The aim of this study was to assess the safety and efficacy of currently available melanoma treatments by conducting a systematic review and network meta-analysis. Four databases were systematically searched for randomized clinical studies that included patients with advanced/metastatic melanoma receiving chemotherapy, immune checkpoint inhibitors, BRAF/MEK inhibitor therapy, or combinations thereof. The primary endpoints were treatment-related adverse events (TRAE), serious adverse events (SAE) of grade ≥ 3 adverse events, therapy discontinuation, progression-free survival (PFS), as well as objective response rate (ORR) and complete response rate (CRR). A total of 63 articles were eligible for our systematic review; 59 of them were included in the statistical analysis. A separate subgroup analysis was conducted to evaluate the efficacy outcomes, specifically in BRAF-positive patients. Triple combination therapy or triple therapy (inhibiting BRAF, MEK and PD1/PDL1 axis) showed significantly longer progression-free survival compared to BRAF + MEK combination therapies (HR = 0.76; 95% CI 0.64–0.9), but similar objective and complete response rates in BRAF-mutated melanoma. This safety analysis suggests that triple therapy is not inferior to combined immune checkpoint inhibitors (ICI) and BRAF/MEK therapies in terms of serious adverse events and therapy discontinuation rates. However, monotherapies and BRAF/MEK combinations showed notable advantage over triple therapy in terms of treatment-related adverse events. Combination strategies including BRAF/MEK-targeted therapies with ICI therapies are effective first-line options for advanced, BRAF-mutant melanoma; however, they are associated with more frequent side effects. Therefore, future RCTs are required to evaluate and identify high-risk subpopulations where triple therapy therapies should be considered.

## 1. Introduction

Advanced melanoma, defined by the spread of the disease to distant organs, was once considered a diagnosis with limited treatment options. The emergence of targeted therapies and immune checkpoint inhibitors has introduced a new era in melanoma care, establishing them as primary treatment options [1,2].

Approximately 40–60% of melanomas harbor mutations in the BRAF gene, with the V600(E/K) mutation being the most common [1,2]. Alterations of the BRAF gene result in hyperactivation of the mitogen-activated protein kinase (MAPK) signal transduction pathway with subsequent MEK activation, leading to accelerated tumor proliferation and poorer survival [1,2]. An increased efficacy has been observed when the simultaneous inhibition of BRAF and MEK proteins is used, with significant overall survival benefits and accelerated tumor regression [3,4]. The most common side effects of combination therapy—such as fever and chills, fatigue, diarrhea, hypertension, and vomiting—often require dose interruptions and adjustments to maintain continuous treatment [5].

For melanoma, immune checkpoint inhibitors (ICIs) have emerged as a key treatment option. By blocking immune checkpoint proteins, such as programmed cell death receptor 1 (PD-1) and T lymphocyte-associated antigen 4 (CTLA-4) among others, ICIs enhance T-cell mediated antitumor response [6,7]. Immunotherapy has shown promising results in improving overall survival rates and prolonging the duration of response in patients with BRAF wild-type melanoma [8,9,10]. Immune-related adverse events (irAE) are diverse and different from targeted therapies or conventional cytotoxic therapies in timing and clinical manifestation [11,12]. These toxicities primarily arise from disrupted immune tolerance. CTLA-4 inhibitors frequently lead to colitis, hypophysitis, and rash, while PD-1/PD-L1 inhibitors are more commonly associated with pneumonitis, colitis, and thyroiditis. The combination of PD-1/PD-L1 and CTLA-4 inhibitors is often linked to cutaneous and endocrine toxicities. [13] Early recognition allows for many adverse events to be reversible and manageable through supportive care or immunosuppressive therapy [11].

Recent publications suggest that triple therapy—BRAF-inhibitor (BRAFi) + MEK-inhibitor (MEKi) coupled with PD-1 or PD-L1 immunotherapy—demonstrates remarkable antitumor activity and a lower risk of progressive disease [14,15,16]. However, these innovative treatment combinations also present challenges, as both immune checkpoint inhibitors and targeted therapies can trigger a range of side effects, which may include autoimmune reactions, skin toxicities, and constitutional symptoms [17,18,19]. Frequent side effects raise the critical issue of managing adverse events, emphasizing the need for vigilant monitoring and proactive interventions to ensure the well-being of patients undergoing such treatments [5,20,21].

The aim of this study was to evaluate the relative safety and, where possible, the efficacy of pharmacological treatment options and combination strategies in advanced melanoma—in particular, triple combination therapy—by conducting a systematic review and network meta-analysis. We sought to expand upon the existing limited data on the relative safety and efficacy of triple therapy compared to targeted therapies or combined immunotherapies.

## 2. Method

### 2.1. Protocol and Registration

To report our systematic review and network meta-analysis, the recommendations of the Preferred Reporting Items for Systematic Reviews and Meta-Analyses (PRISMA) 2020 guideline [22] (Table S14) and the latest version of the Cochrane Handbook were followed to conduct our network meta-analysis [23]. The predefined protocol of this study was registered on PROSPERO (registration number: CRD42022376080).

### 2.2. Eligibility Criteria

The eligible articles reported on the randomized controlled trials of patients with advanced or metastatic stage 3 and stage 4 melanoma who received chemotherapy, targeted BRAF and/or an MEK inhibitor, immune checkpoint inhibitor (PD-1, PD-L1, CTLA-4) therapy, or any combination of these. The trials comparing different chemotherapy regimens were excluded. Trials were included in our analysis if they provided data on adverse events, the therapy discontinuation rate, and any of the following: progression-free survival, overall survival, or the objective or complete therapy response rate.

### 2.3. Information Sources

Our systematic literature search was conducted on the 5 March 2023. Four databases were searched: Medline, Embase, CENTRAL (Cochrane Library), and Web of Science.

### 2.4. Search Strategy

The search terms used for the systematic search and the number of hits can be found in Supplementary Materials (Table S1). For “all fields search” on Web of Science, we modified the search key, which is shown in Table S1.

### 2.5. Selection Process

A reference library was created using EndNote20 (Clarivate Analytics, Philadelphia, PA, USA) [24]. After duplicate removal, the selection process was performed by using a software tool from Rayyan Systems (Cambridge, MA, USA) [25]. Two authors (NG, ASL) independently selected the articles by title, abstract, and full text, with any disagreement resolved by consensus or by a third author (FAM).

### 2.6. Data Collection Process

The data from eligible articles were retrieved by four reviewers independently (AAM, PNP, AITL, ASL, MK) using a pre-designed sheet. Disagreements were solved by a fifth (FAM) and a sixth (LVK) author. For the studies providing survival data only for Kaplan–Meier curves, WebPlotDigitizer was used for data extraction [26,27].

### 2.7. Data Items

The data extracted included the first author, the year of publication, the study population, the study period, the population characteristics, the assigned intervention, the control treatment, and all the outcome parameters (ORR, CRR, PFS, OS, TRAE, SAE, grade 3 ≤ AE, therapy discontinuation rate).

### 2.8. Study Risk of Bias and Certainty of Evidence

The risk of bias was assessed independently by five authors (AAM, PNP, AITL, ASL, MK) using version 2 of the Cochrane Risk-of-Bias Tool for Randomized Trials (RoB-2) [28]. Each trial outcome was assessed individually for the risk of bias as “low”, “some risk”, or “high”. Any disagreement was resolved by ASL and FAM.

The Confidence in Network Meta-Analysis (CINEMA version 2.0.0), and Grading of Recommendations, Assessment, Development, and Evaluation (GRADE) software tools were used to assess the certainty of evidence for all the outcome parameters in the main analysis [29,30].

### 2.9. Synthesis Methods

A network meta-analysis was performed [31]. As considerable between-study heterogeneity was assumed in all the cases, a random-effects model was used to pool the effect sizes. An effect size measure hazard ratio (HR) with a confidence interval (CI) of 95% was used. Furthermore, an odds ratio (OR) with a confidence interval of 95% (CI) was used for the effect size measure of dichotomous data. To calculate the odds ratio, the total number of patients and those with the event of interest in each group separately (“raw data”) were extracted or calculated from the studies where available.

A network plot was used to visualize the treatments, with nodes representing the different treatments, and the edges representing direct comparisons. Evidence plots were created to visualize the direct/indirect comparisons of treatments for each outcome. The network estimates (pooled estimates of direct and indirect data) of each treatment are presented in the league tables. The interventions were ranked by calculating the P-score. It ranged from 0 to 1, with 0 or 1 being the theoretically worst or best treatment, respectively. The consistency was examined using forest plot that could help identify loops where inconsistency was present. An Egger’s test and funnel plots were used to graphically assess the publication bias. A statistical analysis of the data was performed using the R software version 4.2.2. using the BUGSnet version 1.1.0 and Netmeta version 2.7-0 [32,33,34] packages for calculations. The primary, grouped analysis included pooled interventions: therapies that were pooled into treatment modalities based on their mechanism of action. To enhance the internal validity of our networks, we also pooled various PD-1 and PD-L1 inhibitory agents to maximize the similarity within the intervention nodes and minimize it between the nodes. The second, stratified sub-analysis investigated each drug or drug combination against each other. Separate, subgroup analysis was performed for the BRAF-mutant subgroup receiving first-line triple therapy versus BRAF/MEK therapy. However, for the BRAF-mutant subgroup analysis of efficacy outcomes, we repeated the analysis without pooling the PD-1/PD-L1-containing triple therapies, thereby accounting for the differences between PD-1 and PD-L1 therapies to be reflected.

## 3. Results

### 3.1. Search and Selection

**Figure 1:**
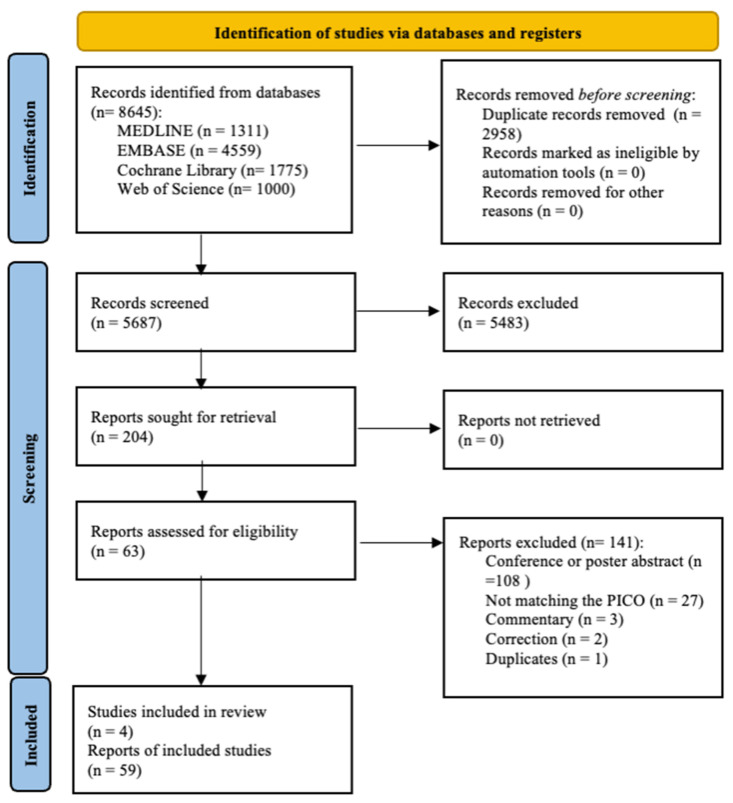
PRISMA 2020 flow diagram for the selection process.

In total, 8645 studies were identified by our search terms: Medline (1311), Embase (4559), Cochrane Library (1775), and Web of Science (1000). After duplicate removal, 5687 individual articles were screened. The articles that compared different doses of an intervention or examined sequential dosing were excluded from the statistical analysis. A total of 63 studies [4,8,9,10,14,15,16,35,36,37,38,39,40,41,42,43,44,45,46,47,48,49,50,51,52,53,54,55,56,57,58,59,60,61,62,63,64,65,66,67,68,69,70,71,72,73,74,75,76,77,78,79,80,81,82,83,84,85,86,87,88] were included in this analysis, 59 of these studies [4,8,9,10,14,15,16,35,36,37,38,39,40,41,42,43,44,45,46,47,48,49,50,51,52,53,54,55,56,57,58,59,60,61,62,63,64,65,66,67,68,69,70,71,72,73,74,75,76,77,78,79,80,81,82,83,84] contributing data from 10,679 patients to the NMA (Figure 1). The Cohen’s kappa coefficient was 0.94 for title and abstract selection and κ = 0.9 for full-text selection.

### 3.2. Main Characteristics of Included Studies

All the studies included in the qualitative and quantitative analyses were randomized controlled trials; the characteristics of the included studies are detailed in Table 1.

### 3.3. Quantitative Analysis

**Figure 2:**
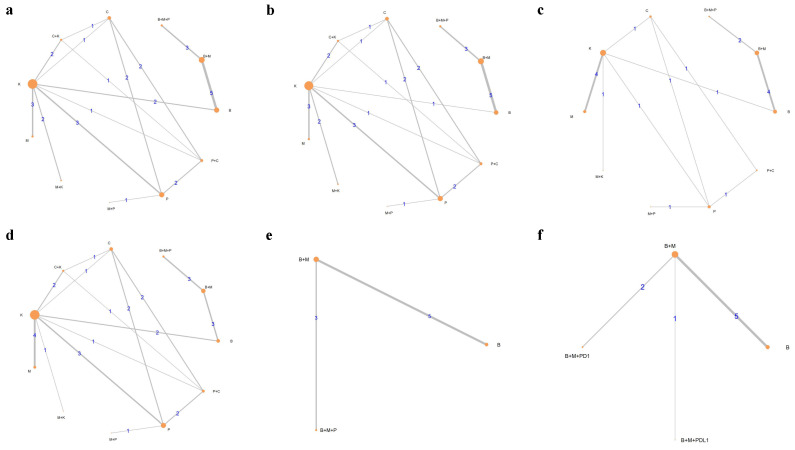
Comparative network plots for interventions, with nodes representing treatments and the edges indicating direct comparisons between them. The size of the nodes is proportional to the number of patients assigned to the therapy. (**a**) TRAE; (**b**) grade 3 ≤ AE; (**c**) SAE OS; (**d**) TDR; (**e**) ORR, CRR, PFS, OS; (**f**) ORR, CRR, PFS, OS. Abbreviations: ORR, objective response rate; CRR, complete response rate; PFS, progression-free survival; OS, overall survival; AE, adverse event; TRAE, treatment-related adverse event; SAE, serious adverse event; TDR, treatment/therapy discontinuation rate; B, BRAF inhibitor; M, MEK inhibitor; P, PD-(L)1 inhibitor; PD1, PD-1 inhibitor; PDL1, PD-L1 inhibitor; C, CTLA-4 inhibitor; K, chemotherapy.

To evaluate the safety-related endpoints, we performed two independent network meta-analyses. First, we grouped the treatments targeting identical pathways (e.g., PD1 and PDL1 inhibitors), which together allowed more robust and reliable comparisons between the various treatment modalities. Similarly, different BRAF and MEK inhibitors were pooled. Second, we performed an alternative, stratified analysis without grouping similar treatment modalities, which allowed for a detailed examination of each pharmaceutical therapy. Due to the lack of reported data on serious adverse events (SAEs), only a pooled analysis was performed for the SAEs. Figure 2 shows comparative network plots for the relevant outcomes based on treatment modalities. Supplementary Figure S3 shows networks of the stratified, second approach results in a weak network, suggesting that a pooled approach (Figure 2) is superior in terms of connectivity for safety-related endpoints. A leave-one-out analysis was also performed for efficacy outcomes, excluding studies specifically targeting brain metastatic-only patients (NIBIT-M2 and NCT02374242); the results can be found in Supplementary Materials (Figure S3, Table S4b). However, leaving the individual studies out did not significantly alter any of the conclusions.

**Figure 3:**
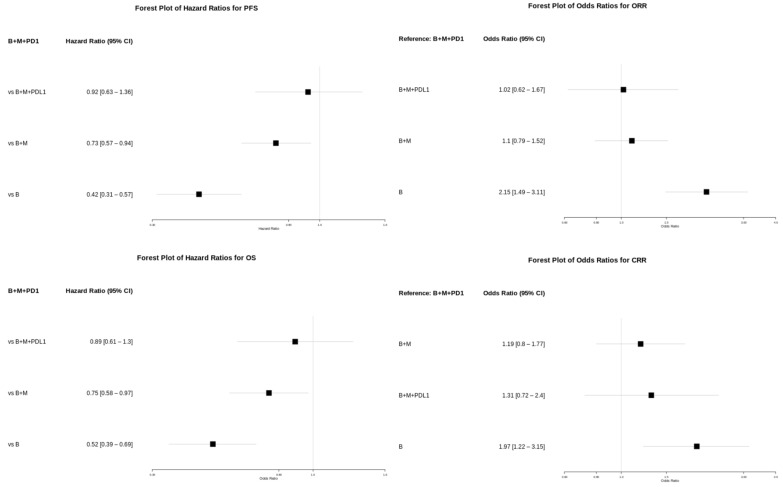
Forest plots for key efficacy outcomes in BRAF-mutant subgroup. Abbreviations: CI, confidence interval; B, BRAF inhibitor; M, MEK inhibitor; PD1, PD-1 inhibitor; PDL1, PD-L1 inhibitor.

To evaluate the efficacy-related endpoints in a homogenous patient population, we performed a subgroup analysis of the first, pooled approach focusing on the BRAF-mutated patients receiving first-line therapy only, mostly without CNS metastasis. This allowed the comparison of first-line BRAF/MEK therapy or triple therapy in advanced BRAF-mutated melanoma patients in the first-line setting (Figure 2e,f). Forest plots were created for key outcomes of the subgroup analysis to improve data visualization (Figure 3).

#### 3.3.1. Safety Outcomes

The safety and therapy discontinuations were analyzed using odds ratios (ORs) as an effect measure. The odds ratios were calculated separately from the reported events in each group for each trial (“raw data”). The P-scores showed the relative superiority of the PD-(L)1 inhibitors and K (chemotherapy) in terms of therapy discontinuation and adverse events (Table 2). Additional results from the stratified sub-analysis for adverse events and therapy discontinuation are in Supplementary Materials (Table S5).

As expected, generally, monotherapies outperformed other interventions regarding the number and type of treatment-related adverse events (TRAE). Focusing on combination therapies, in terms of serious adverse events (SAE), the odds ratio for the triple therapy arm was not significantly lower than that for combined immunotherapy (OR 0.67; 95% CI 0.09–4.79). It is important to note that in the studies, combined ICI therapy was administered at a dosage of 1 mg/kg nivolumab in combination with 3 mg/kg ipilimumab. The BRAF/MEK therapy compared to the triple therapy did not show a significantly lower risk for developing an SAE OR 0.62 95% CI 0.29–1.33 (Table 3). Generally, the risk of early therapy discontinuation was lower for individuals on monotherapies. The risk for therapy interruption was not significantly different in dual BRAF/MEK therapy compared to triple therapy (OR 0.61; 95% CI 0.29–1.28). Furthermore, the OR for triple combination compared to PD-1 + CTLA-4 therapy was lower, but not significantly (OR 0.56; 95% CI 0.09–3.51) (Table 3).

However, the occurrence of treatment-related adverse events (TRAE) and grade 3 ≤ adverse events were among the highest for triple therapy. Dual BRAF and MEK inhibition showed significantly lower risk for treatment-related grade 3 ≤ adverse events compared to triple therapy OR 0.42; 95% CI 0.23–0.77; however, ICI combination therapy (PD-1 + CTLA-4) was not associated with significantly less grade 3 ≤ adverse events compared to triple therapy OR 0.25 95% CI 0.06–1.05 (Table 3).

#### 3.3.2. Efficacy Outcomes

A statistical analysis was performed using a hazard ratio (HR) as the effect measure for progression-free survival and overall survival and an odds ratio (OR) for the objective and complete therapy response. The interventions were ranked according to their relative probability of being superior treatment using random P-scores (Table 2). The results presented here focus on the BRAF-mutant, first-line therapy subgroup; additional findings can be found in the Supplementary File.

An analysis of the progression-free survival times showed that longer progression-free survival times can be achieved with triple therapy. When the treatments were pooled into treatment modalities, PD1/PD-L1 + BRAF + MEK combination therapy showed a 24% lower risk for disease progression compared to BRAF + MEK (HR 0.76; 95% CI 0.64–0.9) (Table 4). An indirect comparison between PD1 + BRAF + MEK and PD-L1 + BRAF + MEK therapy suggested no significant superiority of PD-1-containing interventions.

The overall survival times were slightly longer with triple therapy than with BRAF + MEK therapy (HR 0.79; 95% CI 0.66–0.96) (Table 4). Similarly to the PFS findings, the triple therapy regimens that included PD-1 inhibitors appeared to provide a similar survival benefit to those based on PD-L1-based inhibitors, with P-scores of 90% and 71% (Table 2 and Table 4, Figure 3). However, triple therapy did not yield a significantly better objective response compared to dual BRAF and MEK inhibition (OR 1.09; 95% CI 0.85–1.39). Similarly, the complete response rate was also not better in the triple therapy group compared to BRAF/MEK therapy OR 1.06; 95% CI 0.79–1.43 (Table 4). Additional efficacy outcomes are presented in Supplementary Materials (Tables S3–S4). Collectively, these results do not suggest the superior efficacy of triple therapies compared to BRAF/MEK combination therapies.

### 3.4. Systematic Review

The studies that could not be statistically analyzed due to sequential or dose-comparison interventions are summarized in Table S2. The CheckMate 511 trial evaluated two dosing regimens of combination immunotherapy and concluded that nivolumab 3 mg/kg +ipilimumab 1 mg/kg resulted in a lower incidence of grade 3 ≤ adverse events with similar survival times [85]. The CheckMate 064 study established the optimal sequence of ICI monotherapy: nivolumab followed by ipilimumab results in longer progression-free and overall survival and higher response rates than reversed administration [88]. For the sequential administration of targeted therapy and immunotherapy, two independent trials (Dreamseq, Secombit) came to the same conclusion that immunotherapy followed by the continuation of BRAF/MEK-targeted therapy could be the most beneficial for individuals with advanced melanoma harboring BRAF mutations [86,87].

### 3.5. Risk of Bias Assessment and Certainty of Evidence

The risk of bias assessment of the RCTs showed a predominantly low to medium risk of bias, with only some outcomes having a high risk of bias. However, IMspire170, NCT00338130, NEMO, and the NIBIT-M2 trials are suspected of having a high risk of bias due to their randomization process. The detailed evaluation is presented in Supplementary Table S6.

The funnel plots for our outcomes showed no evidence of publication bias; an Egger’s test for the regression intercept gave *p*-values between 0.0705 and 0.8941, indicating no evidence of publication bias (Figures S19–S33). For subgroup analysis, only the funnel plots were drawn; an Egger’s test could not be applied. A consistency assessment was performed, revealing no substantial inconsistencies at any endpoints (Figures S34–S48). However, this may be due to our broad inclusion criteria, which may have complicated the effectiveness of an inconsistency analysis. Our patient population was quite heterogeneous in terms of disease stage and tumor mutational status (except for the subgroup analysis that only consisted of BRAF-mutant patients receiving first-line therapies). The certainty of evidence was mostly low to moderate across the outcomes due to the low number of direct comparisons and the substantial heterogeneity among studies. The detailed results of the CINEMA version 2.0.0 and GRADE assessments can be found in Supplementary Materials (Tables S7–S13).

## 4. Discussion

The main aim of our systematic review and network meta-analysis was to synthesize the available evidence on the safety of various treatment options in advanced melanoma. To our knowledge, the current study utilized the largest patient population investigating the safety and efficacy of combination therapy approaches in advanced melanoma. We included three published trials on triple therapy versus BRAF/MEK-targeted therapy; however, there are ongoing studies on triple therapy (STARBOARD, PORTSIDE, SWOG S2000), and their findings could serve as a valuable contribution to the topic and future meta-analyses [89,90,91]. Previous network meta-analyses were already performed on this topic [92,93]. Boutros and colleagues had comparable results to ours; based on their findings, triple therapy compared to ipilimumab/nivolumab or BRAF/MEK therapy showed better PFS; on the other hand, the response rate of triple therapy was similar to BRAF/MEK [93]. In terms of the adverse event, combined ICI was the least optimal and BRAF/MEK was better tolerated than triple therapy [92,93].

The safety outcomes were generally favorable for monotherapies, with lower rates for all and any type of adverse events. The analysis of TRAE with a grade 3 ≤ AEs and SAE highlighted that the populations receiving PD1/PD-L1 intervention reported the lowest number of events, suggesting a favorable safety profile for these treatments. Furthermore, the individuals on triple therapy compared to BRAF/MEK showed a higher risk for grade 3 ≤ adverse events, but no statistical difference in terms of serious adverse events or therapy discontinuation rates. Indirect evidence from the SAEs with a grade 3 ≤ AEs and a treatment interruption analysis suggested that triple therapy is not inferior to combined ICIs. However, given the limited effectiveness of triple therapy, it may not be recommended as a first-line treatment option. These findings underscore the importance of carefully balancing treatment efficacy with tolerability, especially in multi-agent regimens. Alternative strategies, such as treatment sequencing or intermittent administration, could potentially reduce toxicity while maintaining comparable efficacy [94].

In terms of objective and complete response, triple therapy showed no advantage compared to BRAF/MEK inhibitors. A treatment combination of PD-1/PD-L1 with BRAF/MEK inhibitors showed the longest progression-free survival times. On the other hand, the statistically significant effect of triple therapy in clinical practice was less explicit [95]. A sensitivity analysis for the pooling of PD1 and PDL1 inhibitor-containing triple therapies revealed no difference between the inhibition of two ligands. Identifying high-risk patient populations who may benefit from the potential extended PFS associated with triple therapy remains an open question. A recently published study suggests triple therapy as a treatment option for patients who have already failed targeted therapies and immunotherapies, and it also highlights the potential benefit of triple therapy in patients with CNS metastasis combined with radiotherapy [96]. A valuable addition to this topic is the ongoing SWOG S2000 study directly comparing first-line triple therapy with ipilimumab plus nivolumab in participants with brain metastasis [90]. These findings highlight the potential of combining targeted therapies and immunotherapy and also raise the potential benefits of quadruple therapy, as previously mentioned by others [97]. In addition, it is possible that sequential therapy options may carry the benefit of triple therapy with a favorable side effect profile. The results from the Dreamseq and Secombit trials suggest a potential sequence for ICIs and targeted therapy [86,87]. First-line immunotherapy followed by a switch to targeted BRAF + MEK therapy could be the most beneficial for patients with BRAF-mutated melanoma. A similar conclusion can be drawn from the recent IMPemBra trial that immunotherapy followed by intermittent dabrafenib plus trametinib provides a survival benefit, reduces the risk of toxicity presented by triple therapy, and lowers the chance of acquired resistance to targeted therapy [94].

### 4.1. Strengths and Limitations

To our knowledge, this investigation represents the most recent and largest comprehensive analysis of all the available RCTs of advanced melanoma. Pooling treatment modalities allowed us to comprehensively summarize all the endpoints and confirm the indirect comparisons. The network meta-analysis setting allowed us to indirectly compare immunotherapies and targeted therapies. We conducted multiple analyses to eliminate the potential biases arising from pooling subgroup analyses for BRAF-positive patients receiving first-line treatment to minimize heterogeneity.

A key limitation of our analysis was its between-study heterogeneity, which affected the efficacy analyses more than the safety analyses. Our inclusion criteria were broad, including studies with both BRAF-mutant and BRAF wild-type melanomas without distinguishing the byline of therapy. Additionally, a notable limitation was the inclusion of studies with only brain metastasis patients. To mitigate this effect, we conducted efficacy analyses excluding the NCT02374242 and NIBIT-M2 studies. Another limitation was the inclusion of older studies that involved chemotherapy, where crossover to the investigational drug was allowed, though the crossover adjustments were not consistently detailed. Another limitation of the current study was the exclusion of LAG-3 inhibitor studies such as Relativity-047, justified by the lack of triple-therapy approaches using LAG-3 inhibitors [98]. A notable limitation was the disparities in the follow-up time for the adverse events throughout the studies, and that the individual patient data could not be obtained to sufficiently analyze the types of adverse events.

### 4.2. Implications for Practice and Research

The translation of scientific knowledge into immediate use in healthcare is crucial for the benefit of patients [99,100]. Our network meta-analysis highlights the importance of a personalized consideration for high-risk individuals. Given the modest evidence in effectiveness and the side-effect profile of first-line triple therapy, a sequential approach may benefit some patients more than concurrent triple therapy. Triple therapy could be more suitable as a second- or third-line option, depending on the tumor characteristics, disease burden, and tolerance for side effect [96]. Further specific prospective randomized multicentric studies will be needed to refine the use of triple therapy and the identification of the optimal patient population.

## 5. Conclusions

Combined medications for melanoma management generally carry elevated risks for developing adverse events. The increased risk of serious adverse events and therapy discontinuation is crucial for healthcare providers to anticipate, manage, and communicate with patients effectively. Based on our findings, the effectiveness of triple therapy as a first-line intervention in BRAF-mutant melanoma is not well supported; additional studies are required to identify the potential population who could still benefit from triple therapy.

## Figures and Tables

**Table 1:** Basic characteristics of studies included in the network meta-analysis. Abbreviations: ICC, investigator’s choice chemotherapy.

Study Name	Study Period	Population	Treatment	N	Male (N)	Age †
BREAK-3 (NCT01227889) [55,65]	23 December 2010–19 December 2011	Patients with stage 4 or unresectable stage 3, BRAF V600E mutated.	dabrafenib	187	112	53·0 (22–93)
			dacarbazine	63	37	50·0 (21–82)
CheckMate 037 (NCT01721746) [10,64]	21 December 2012–10 Jan 2014	Unresectable stage 3C or 4 metastatic melanoma patients, BRAF wild-type or BRAFV600 mutation-positive tumor.	nivolumab	272	176	59 (23–88)
			ICC (dacarbazine or carboplatin plus paclitaxel)	133	85	62 (29–85)
CheckMate 066 (NCT01721772) [38,40,78,79]	January 2013–February 2014	Unresectable, previously untreated stage 3 or 4 melanoma patients without a BRAF mutation.	nivolumab	210	121	64 (18–86)
			dacarbazine	208	125	66 (26–87)
CA184-024 (NCT00324155) [9,72]	8 August 2006–22 January 2008	Previously untreated stage 3 (unresectable) or stage 4 melanoma with measurable lesions.	ipilimumab plus dacarbazine	250	152	mean age: 57.5
			dacarbazine	252	149	mean age: 56.4
METRIC (NCT01245062) [50,77]	22 November 2010–26 October 2011	Patients with unresectable stage 3C or 4 cutaneous melanoma with a V600E or V600K BRAF mutation.	trametinib	214	120	55 (23–85)
			dacarbazine or paclitaxel	108	53	54 (21–77)
BRIM-3 (NCT01006980) [41,42]	4 January 2010–16 December 2010	Patients with unresectable, previously untreated stage 3C or stage 4 melanoma that tested positive for the BRAF V600E.	vemurafenib	337	200	56 (21–86)
			dacarbazine	338	181	52 (17–86)
PACMEL (EudraCT:2011-002545-35) [83]	April 2012–March 2016	Patients with measurable unresectable BRAF wild-type stage 3 or 4 melanoma.	paclitaxel–trametinib	36	23	60 (27–80)
			paclitaxel	38	27	64 (35–80)
NCT00936221 [76]	20 July 2009–8 April 2010	Patients with advanced (stage 3 and 4), BRAF-mutant, cutaneous or unknown primary melanoma were included.	selumetinib plus dacarbazine	45	22	57 (48–69)
			dacarbazine	46	28	52 (40–65)
Keynote 002 (NCT01704287) [54,75]	30 November 2012–13 November 2013	Patients with confirmed unresectable stage 3 or stage 4 melanoma, confirmed disease progression of the last ipilimumab dose, previous BRAF or MEK inhibitor therapy or both (if BRAFV600 mutant-positive)	pembrolizumab	181	109	60 (27–89)
			ICC (paclitaxel plus carboplatin, paclitaxel, carboplatin, dacarbazine, or temozolomide)	179	114	63 (27–87)
Ribas 2013 [NCT00257205) [74]	March 2006–July 2007	Patients with stage 3C or 4, measurable, unresectable melanoma	tremelimumab	328	190	mean age 57 (range: 22–90)
			DTIC or temzolomide	327	182	mean age 56 (range: 22–90)
NCT01693068 [66]	December 2012–July 2015	Patients with measurable, unresectable locally advanced or metastatic cutaneous melanoma (stage 3C or 4 [M1a-c]) NRAS mutated.	pimasertib	130	68	65 (21–83)
			DTIC	64	36	62 (23–83)
NCT00338130 [60]	July 2006–September 2007	Patients with confirmed unresectable stage 3 or 4 malignant melanoma.	selumetinib	104	55	mean age 57,1 (range: 20–84)
			temozolomide	96	65	mean age 57 (range: 28–84)
NCT00050102 (CA184013) [56]	September 2002–August 2004	Patients with histologic diagnosis of unresectable metastatic melanoma and progressive disease.	ipilimumab with DTIC	35	26	60 (27–82)
			ipilimumab	37	21	66 (25–82)
NEMO (NCT01763164) [48]	19 August 2013–28 April 2015	Patients with histologically confirmed locally advanced unresectable or metastatic cutaneous melanoma harboring an NRAS mutation.	binimetinib	269	166	65 (18–90)
			dacarbazine	133	85	62 (27–89)
NIBIT-M2 (NCT02460068) [43]	January 2013–September 2018	Patients with BRAF wild-type or mutant melanoma, and active, untreated, asymptomatic brain metastases.	ipilimumab with nivolumab	27	17	56 (25–79)
			fotemustine and ipilimumab	26	16	60 (31–74)
			fotemustine	23	15	57 (20–80)
DOC-MEK (NCT01256359) [52]	October 2010–May 2012	Participants with unresectable stage 3 or 4 BRAF wild-type melanoma.	selumetinib and docetaxel	41	31	62 (52–68)
			docetaxel	42	27	63 (49–68)
Keynote-022 (NCT02130466) [14,37]	30 November 2015–24 April 2017	Patients with previously untreated advanced melanoma, unresectable stage 3/ 4 cutaneous melanoma, BRAF V600E/K mutation positive. Patients should not have previously received systemic therapy.	pembrolizumab with dabrafenib and trametinib	60	33	54 (18–82)
			dabrafenib and trametinib	60	36	58 (21–82)
COMBI-I (NCT02967692) [15]	13 September 2017–4 July 2018	Histologically confirmed unresectable or metastatic BRAF V600–mutant cutaneous melanoma without prior systemic anticancer treatment.	spartalizumab with dabrafenib and trametinib	267	NA	56 (46–66)
			dabrafenib and trametinib	265	NA	55 (47–65)
IMspire150 (NCT02908672) [16,53]	13 January 2017–26 April 2018	Patients with unresectable stage 3C, BRAFV600 mutation positive melanoma, patients had not received previous systemic treatment.	atezolizumab plus vemurafenib and cobimetinib	256	150	54 (44,8–64)
			vemurafenib and cobimetinib	258	149	53,5 (43–63,8)
IMspire170 (NCT03273153) [51]	11 December 2017–29 January 2019	Patients with locally advanced and unresectable or metastatic melanoma that was negative for BRAFV600.	cobimetinib plus atezolizumab	222	129	66 (54–73)
			pembrolizumab	224	141	66 (55–73)
coBRIM (NCT01689519) [35,39,44,61]	8 January 2013–31 January 2014	Patients with histologically confirmed unresectable stage 3C or stage 4 melanoma harboring a BRAFV600 mutation	cobimetinib with vemurafenib	247	146	56 (23–88)
			vemurafenib	248	140	55 (25–85)
Keynote-006 (NCT01866319) [80,81,82]	18 September 2013–3 March 2014	Patients with unresectable stage 3 or 4 melanoma and up to one previous systemic therapy. Additional eligibility criteria included known BRAF status.	pembrolizumab	279	161	61 (18–89)
			pembrolizumab	277	174	63 (22–89)
			ipilimumab	278	162	62 (18–88)
CheckMate 067 (NCT01844505) [8,58,62,63,84]	July 2013–March 2014	Eligible adult patients had previously untreated and unresectable or metastatic histologically confirmed stage 3 or stage 4 melanoma with known BRAF V600 mutation status.	nivolumab plus ipilimumab	314	206	mean age 59 (range: 18–88)
			nivolumab	316	202	mean age 59 (range: 25–90)
			ipilimumab	315	202	mean age 61 (range: 18–89)
Combi-v (NCT01597908) [4]	June 2012–June 2014	Patients with metastatic melanoma with a BRAF V600 mutation.	dabrafenib and trametinib	352	208	55 (18–91)
			vemurafenib	352	180	54 (18–88)
COMBI-d (NCT01584648) [3,69,70]	May 2012–January 2013	Previously untreated patients who had unresectable stage 3C or stage 4 melanoma with a BRAF V600E/K mutation without previous systemic anticancer therapy (including BRAF or MEK inhibitors).	dabrafenib and trametinib	211	111	55 (22–89)
			dabrafenib	212	114	56.5 (22–86)
COLUMBUS (NCT01909453) [36,45,46,47]	30 December 2013–10 April 2015	Patients have a histologically confirmed locally advanced stage 3B/C, or 4, unresectable or metastatic cutaneous melanoma, have the presence of a BRAFV600E or BRAFV600K mutation.	encorafenib plus binimetinib	192	115	57 (20–89)
			encorafenib	194	108	54 (23–88)
			vemurafenib	191	111	56 (21–82)
CheckMate 069 (NCT01927419) [57,73]	2013–2014	Patients aged ≥18 years with previously untreated, unresectable stage 3 or 4 melanoma, with known BRAFV600 mutation status.	nivolumab plus ipilimumab	95	63	64 (27–87)
			ipilimumab	47	32	67 (31–80)
NCT02374242 (Part A,B) [67]	4 November 2014–21 April 2017	Patients with histologically confirmed stage 4 melanoma (excluding ocular melanoma), had at least one target intracranial lesion.	nivolumab with ipilimumab	35	29	59 (53–68)
			nivolumab	25	19	63 (52–74)
NCT01072175 (Part C) [49,59,68,71]	26 March 2010–31 May 2012	Patients who had histologically confirmed metastatic melanoma with either BRAF V600E/K mutations.	dabrafenib	54	29	50 (18–82)
			dabrafenib and trametinib	54	34	49 (23–85)

† median (range) unless otherwise stated. ICC: investigator’s choice chemotherapy.

**Table 2:** Random P-scores of interventions based on the relative probability of being better compared to another. Abbreviations: ORR, objective response rate; CRR, complete response rate; PFS, progression-free survival; OS, overall survival; AE, adverse event; TRAE, treatment-related adverse event; SAE, serious adverse event; TDR, treatment/therapy discontinuation rate; B, BRAF inhibitor; M, MEK inhibitor; P, PD-(L)1 inhibitor; PD1, PD-1 inhibitor; PDL1, PD-L1 inhibitor; C, CTLA-4 inhibitor; K, chemotherapy.

TRAE		SAE		Grade 3 ≤ AE		TDR	
Treatment	P-Score	Treatment	P-Score	Treatment	P-Score	Treatment	P-Score
K	0.9175	K	0.9700	P	0.9714	K	0.9728
P	0.8585	P	0.8773	K	0.8775	P	0.8015
C	0.8003	C	0.6481	C	0.8403	B	0.6698
B+M	0.5819	M	0.6475	P+C	0.5031	C	0.6240
P+C	0.4986	B	0.4864	M	0.4932	M+K	0.5224
B	0.4866	M+P	0.4622	M+P	0.4681	M+P	0.4605
C+K	0.4764	M+K	0.4378	C+K	0.3979	M	0.4544
M+K	0.4486	B+M	0.2667	B	0.3819	B+M	0.4528
M	0.1664	B+M+P	0.1253	B+M	0.2789	B+M+P	0.2539
B+M+P	0.1552	P+C	0.0785	M+K	0.2541	C+K	0.2001
M+P	0.1098			B+M+P	0.0335	P+C	0.0880
ORR		CRR		PFS		OS	
B+M+P	0.8745	B+M+P	0.8216	B+M+P	0.9995	B+M+P	0.9958
B+M	0.6255	B+M	0.6770	B+M	0.5005	B+M	0.5042
B	0.0000	B	0.0014	B	0.0000	B	0.0000
ORR		CRR		PFS		OS	
B+M+PD1	0.7478	B+M+PD1	0.8724	B+M+PD1	0.8842	B+M+PD1	0.9082
B+M+PDL1	0.7057	B+M	0.6188	B+M+PDL1	0.7608	B+M+PDL1	0.7106
B+M	0.5465	B+M+PDL1	0.4864	B+M	0.3549	B+M	0.3811
B	0.0001	B	0.0224	B	0.0000	B	0.0001

Adverse Events (TRAE, Grade 3 ≤ AE, SAE, Discontinuation Rate).

**Table 3:** League table of safety outcomes in advanced melanoma. Pooled interventions, direct comparisons are marked in light gray, and indirect (network) results are in white. Abbreviations: OR, odds ratio; CI, confidence interval; B, BRAF inhibitor; M, MEK inhibitor; P, PD-(L)1 inhibitor; C, CTLA-4 inhibitor; K, chemotherapy.

Adverse Events (OR, 95% CI)										
K	0.86 [0.41; 1.83]	0.45 [0.13; 1.56]	.	1.36 [0.24; 7.83]	0.32 [0.12; 0.87]	0.43 [0.13; 1.44]	0.26 [0.02; 2.66]	0.09 [0.02; 0.38]	.	.
0.87 [0.46; 1.65]	P	1.18 [0.53; 2.59]	.	0.20 [0.07; 0.60]	.	.	.	.	.	0.08 [0.02; 0.28]
0.76 [0.37; 1.58]	0.87 [0.45; 1.68]	C	.	0.46 [0.17; 1.25]	.	0.37 [0.07; 1.93]	.	.	.	.
0.40 [0.10; 1.53]	0.46 [0.10; 2.02]	0.52 [0.11; 2.42]	B+M	.	0.81 [0.33; 2.00]	.	.	.	0.22 [0.07; 0.65]	.
0.34 [0.14; 0.84]	0.39 [0.17; 0.90]	0.45 [0.20; 1.02]	0.86 [0.17; 4.31]	P+C	.	0.83 [0.15; 4.52]	.	.	.	.
0.32 [0.12; 0.87]	0.37 [0.11; 1.20]	0.42 [0.12; 1.45]	0.81 [0.33; 2.00]	0.94 [0.25; 3.61]	B	.	.	.	.	.
0.32 [0.12; 0.85]	0.36 [0.13; 1.04]	0.42 [0.15; 1.15]	0.79 [0.15; 4.19]	0.93 [0.31; 2.81]	0.98 [0.24; 3.97]	C+K	.	.	.	.
0.26 [0.02; 2.66]	0.29 [0.03; 3.31]	0.34 [0.03; 3.90]	0.64 [0.04; 9.53]	0.75 [0.06; 9.20]	0.79 [0.06; 10.08]	0.81 [0.06; 10.24]	M+K	.	.	.
0.09 [0.02; 0.38]	0.10 [0.02; 0.50]	0.12 [0.02; 0.60]	0.23 [0.03; 1.63]	0.27 [0.05; 1.46]	0.28 [0.05; 1.62]	0.29 [0.05; 1.64]	0.36 [0.02; 5.59]	M	.	.
0.09 [0.02; 0.49]	0.10 [0.02; 0.63]	0.11 [0.02; 0.75]	0.22 [0.07; 0.65]	0.25 [0.04; 1.79]	0.27 [0.07; 1.11]	0.27 [0.04; 2.01]	0.34 [0.02; 6.27]	0.95 [0.10; 9.03]	B+M+P	.
0.07 [0.02; 0.28]	0.08 [0.02; 0.28]	0.09 [0.02; 0.38]	0.17 [0.02; 1.21]	0.20 [0.04; 0.92]	0.21 [0.04; 1.20]	0.21 [0.04; 1.12]	0.26 [0.02; 4.12]	0.74 [0.10; 5.63]	0.77 [0.08; 7.32]	M+P
**Serious Adverse Events (OR, 95% CI)**										
K	0.95 [0.30; 2.96]	0.31 [0.12; 0.84]	0.38 [0.21; 0.68]	0.23 [0.09; 0.62]	.	0.22 [0.06; 0.82]	.	.	.	
0.71 [0.30; 1.71]	P	0.73 [0.27; 1.99]	.	.	0.33 [0.12; 0.91]	.	.	.	0.05 [0.00; 0.49]	
0.39 [0.17; 0.88]	0.54 [0.24; 1.20]	C	.	.	.	.	.	.	0.17 [0.04; 0.71]	
0.38 [0.21; 0.68]	0.53 [0.18; 1.51]	0.97 [0.36; 2.67]	M	.	.	.	.	.	.	
0.23 [0.09; 0.62]	0.32 [0.09; 1.20]	0.59 [0.16; 2.14]	0.61 [0.19; 1.91]	B	.	.	0.58 [0.34; 0.97]	.	.	
0.24 [0.06; 0.91]	0.33 [0.12; 0.91]	0.62 [0.17; 2.23]	0.63 [0.15; 2.71]	1.04 [0.20; 5.47]	M+P	.	.	.	.	
0.22 [0.06; 0.82]	0.30 [0.06; 1.49]	0.56 [0.12; 2.67]	0.57 [0.13; 2.45]	0.94 [0.18; 4.95]	0.91 [0.14; 5.97]	M+K	.	.	.	
0.13 [0.04; 0.40]	0.18 [0.04; 0.76]	0.34 [0.09; 1.36]	0.35 [0.10; 1.23]	0.58 [0.34; 0.97]	0.55 [0.10; 3.15]	0.61 [0.11; 3.47]	B+M	0.62 [0.29; 1.33]	.	
0.08 [0.02; 0.32]	0.11 [0.02; 0.57]	0.21 [0.04; 1.03]	0.22 [0.05; 0.95]	0.36 [0.14; 0.90]	0.34 [0.05; 2.30]	0.38 [0.06; 2.53]	0.62 [0.29; 1.33]	B+M+P	.	
0.05 [0.01; 0.23]	0.08 [0.02; 0.30]	0.14 [0.04; 0.49]	0.15 [0.03; 0.68]	0.24 [0.04; 1.37]	0.23 [0.04; 1.24]	0.25 [0.04; 1.80]	0.42 [0.07; 2.56]	0.67 [0.09; 4.79]	P+C	
**Grade ≥ 3 Adverse Events (OR, 95% CI)**										
P	0.82 [0.45; 1.50]	0.81 [0.40; 1.62]	0.19 [0.08; 0.43]	.	0.25 [0.09; 0.67]	.	.	.	.	.
0.78 [0.47; 1.30]	K	0.49 [0.19; 1.29]	2.18 [0.50; 9.56]	0.33 [0.17; 0.61]	.	0.33 [0.14; 0.74]	0.24 [0.09; 0.64]	.	0.19 [0.07; 0.48]	.
0.71 [0.40; 1.23]	0.90 [0.50; 1.63]	C	0.24 [0.11; 0.52]	.	.	0.50 [0.11; 2.29]	.	.	.	.
0.26 [0.14; 0.50]	0.33 [0.17; 0.67]	0.37 [0.20; 0.70]	P+C	.	.	0.19 [0.04; 0.83]	.	.	.	.
0.25 [0.11; 0.57]	0.33 [0.17; 0.61]	0.36 [0.15; 0.85]	0.97 [0.38; 2.50]	M	.	.	.	.	.	.
0.25 [0.09; 0.67]	0.32 [0.10; 0.97]	0.35 [0.11; 1.10]	0.95 [0.29; 3.13]	0.98 [0.27; 3.53]	M+P	.	.	.	.	.
0.21 [0.09; 0.48]	0.27 [0.13; 0.56]	0.30 [0.13; 0.67]	0.81 [0.33; 1.95]	0.83 [0.32; 2.16]	0.85 [0.23; 3.08]	C+K	.	.	.	.
0.19 [0.06; 0.56]	0.24 [0.09; 0.64]	0.27 [0.08; 0.83]	0.72 [0.22; 2.40]	0.74 [0.23; 2.36]	0.76 [0.17; 3.34]	0.89 [0.26; 3.02]	B	0.83 [0.53; 1.31]	.	.
0.16 [0.05; 0.51]	0.20 [0.07; 0.59]	0.22 [0.06; 0.76]	0.60 [0.16; 2.16]	0.61 [0.18; 2.13]	0.63 [0.13; 2.97]	0.74 [0.20; 2.72]	0.83 [0.53; 1.31]	B+M	.	0.42 [0.23; 0.77]
0.15 [0.05; 0.43]	0.19 [0.07; 0.48]	0.21 [0.07; 0.64]	0.56 [0.17; 1.83]	0.58 [0.19; 1.79]	0.59 [0.14; 2.56]	0.70 [0.21; 2.30]	0.78 [0.20; 3.05]	0.95 [0.23; 3.96]	M+K	.
0.07 [0.02; 0.25]	0.08 [0.02; 0.29]	0.09 [0.02; 0.37]	0.25 [0.06; 1.05]	0.26 [0.06; 1.04]	0.27 [0.05; 1.41]	0.31 [0.07; 1.31]	0.35 [0.17; 0.75]	0.42 [0.23; 0.77]	0.45 [0.09; 2.12]	B+M+P
**Therapy Discontinuation Rate (OR, 95% CI)**										
K	0.84 [0.37; 1.88]	0.40 [0.12; 1.32]	0.21 [0.06; 0.80]	0.30 [0.06; 1.46]	.	0.25 [0.11; 0.58]	.	.	0.10 [0.03; 0.33]	0.09 [0.01; 1.03]
0.58 [0.29; 1.15]	P	.	0.94 [0.40; 2.23]	.	0.44 [0.12; 1.65]	.	.	.	.	0.19 [0.07; 0.55]
0.40 [0.12; 1.32]	0.69 [0.18; 2.74]	B	.	.	.	.	0.61 [0.30; 1.26]	.	.	.
0.39 [0.17; 0.86]	0.67 [0.33; 1.36]	0.96 [0.23; 4.05]	C	.	.	.	.	.	0.28 [0.02; 3.66]	0.25 [0.10; 0.64]
0.30 [0.06; 1.46]	0.52 [0.09; 2.92]	0.74 [0.10; 5.44]	0.77 [0.13; 4.59]	M+K	.	.	.	.	.	.
0.25 [0.06; 1.13]	0.44 [0.12; 1.65]	0.63 [0.09; 4.27]	0.66 [0.15; 2.96]	0.85 [0.10; 7.54]	M+P	.	.	.	.	.
0.25 [0.11; 0.58]	0.44 [0.15; 1.29]	0.63 [0.15; 2.70]	0.66 [0.21; 2.08]	0.85 [0.14; 5.11]	1.00 [0.18; 5.50]	M	.	.	.	.
0.25 [0.06; 0.99]	0.42 [0.09; 2.01]	0.61 [0.30; 1.26]	0.64 [0.13; 3.17]	0.82 [0.10; 6.83]	0.97 [0.13; 7.45]	0.97 [0.19; 4.92]	B+M	0.61 [0.29; 1.28]	.	.
0.15 [0.03; 0.73]	0.26 [0.05; 1.45]	0.38 [0.13; 1.05]	0.39 [0.07; 2.29]	0.50 [0.05; 4.74]	0.59 [0.07; 5.19]	0.59 [0.10; 3.54]	0.61 [0.29; 1.28]	B+M+P	.	.
0.12 [0.04; 0.34]	0.21 [0.07; 0.66]	0.31 [0.06; 1.48]	0.32 [0.10; 0.99]	0.41 [0.06; 2.74]	0.49 [0.09; 2.77]	0.49 [0.13; 1.82]	0.50 [0.09; 2.82]	0.82 [0.13; 5.36]	C+K	0.26 [0.04; 1.62]
0.08 [0.03; 0.21]	0.15 [0.06; 0.34]	0.21 [0.05; 0.96]	0.22 [0.10; 0.49]	0.28 [0.04; 1.79]	0.33 [0.07; 1.60]	0.33 [0.10; 1.17]	0.34 [0.06; 1.84]	0.56 [0.09; 3.51]	0.69 [0.21; 2.20]	P+C

**Table 4:** League table of efficacy outcomes in BRAF-mutant patients with advanced melanoma. Pooled interventions and direct comparisons are marked in light gray, and indirect (network) results are in white. Tables should be read from left to right, with “best” treatment in top left-hand corner. Abbreviations: B, BRAF inhibitor; M, MEK inhibitor; P, PD-(L)1 inhibitor; PD1, PD-1 inhibitor; PDL1, PD-L1 inhibitor.

TRAE		SAE		Grade 3 ≤ AE		TDR	
Treatment	P-Score	Treatment	P-Score	Treatment	P-Score	Treatment	P-Score
K	0.9175	K	0.9700	P	0.9714	K	0.9728
P	0.8585	P	0.8773	K	0.8775	P	0.8015
C	0.8003	C	0.6481	C	0.8403	B	0.6698
B+M	0.5819	M	0.6475	P+C	0.5031	C	0.6240
P+C	0.4986	B	0.4864	M	0.4932	M+K	0.5224
B	0.4866	M+P	0.4622	M+P	0.4681	M+P	0.4605
C+K	0.4764	M+K	0.4378	C+K	0.3979	M	0.4544
M+K	0.4486	B+M	0.2667	B	0.3819	B+M	0.4528
M	0.1664	B+M+P	0.1253	B+M	0.2789	B+M+P	0.2539
B+M+P	0.1552	P+C	0.0785	M+K	0.2541	C+K	0.2001
M+P	0.1098			B+M+P	0.0335	P+C	0.0880
ORR		CRR		PFS		OS	
B+M+P	0.8745	B+M+P	0.8216	B+M+P	0.9995	B+M+P	0.9958
B+M	0.6255	B+M	0.6770	B+M	0.5005	B+M	0.5042
B	0.0000	B	0.0014	B	0.0000	B	0.0000
ORR		CRR		PFS		OS	
B+M+PD1	0.7478	B+M+PD1	0.8724	B+M+PD1	0.8842	B+M+PD1	0.9082
B+M+PDL1	0.7057	B+M	0.6188	B+M+PDL1	0.7608	B+M+PDL1	0.7106
B+M	0.5465	B+M+PDL1	0.4864	B+M	0.3549	B+M	0.3811
B	0.0001	B	0.0224	B	0.0000	B	0.0001

## Data Availability

The datasets used for out study can be found in the full-text articles included in the systematic review and network meta-analysis.

## Supplementary Materials

The following supporting information can be downloaded at: https://www.mdpi.com/article/10.3390/ijms252312821/s1.
