# Supplementary material for: Safety and Efficacy Analysis of Targeted and Immune Combination Therapy in Advanced Melanoma—A Systematic Review and Network Meta-Analysis
Source: Int J Mol Sci. 2024 Nov 28;25(23):12821. doi: 10.3390/ijms252312821 (PMC11641726; doi:10.3390/ijms252312821)
Supplement: Supplementary file 1 [file ijms-25-12821-s001.zip › ijms-3288229-supplementary.pdf]

Supplementary materials for

Safety and Efficacy Analysis of Targeted and Immune Combination Therapy in Advanced Melanoma — A Systematic Review and Network Meta-analysis

Lengyel A.S., et al.

Abbreviations:

AE: adverse event
Atez: atezolizumab
Bini: binimetinib
CINEMA: Confidence in Network Meta-analysis
Cobi: cobimetinib
CRR: complete response rate
Dabra: dabrafenib
Dac: dacarbazine
Encora: encorafenib
Fote: fotemustine
HR: hazard ratio
ICC: investigator's choice chemotherapy
Ipi: ipilimumab
Nivo: nivolumab
OR: odds ratio
ORR: objective response rate
OS: overall survival
PFS: progression-free survival
Pembro: pembrolizumab
Pima: pimasertib
SAE: serious adverse event
Selu: selumetinib
Sparta: spartalizumab
TDR: treatment/therapy discontinuation rate
TRAE: treatment-related adverse event
Trame: trametinib
Vemura: vemurafenib

Supplementary Table S1. Search terms and number of hits for the databases.
Supplementary Table S2. Characteristics of included studies in systematic review.
Supplementary Figure S3. Comparative network plots.
Supplementary Table S3. P-scores of the pooled and detailed analysis.
Supplementary Table S4a. League tables of efficacy outcomes.
Supplementary Table S4b. League tables of efficacy outcomes excluding NIBIT-M2 and NCT02374242 studies.
Supplementary Table S5. League tables of safety outcomes.
Supplementary Table S6.1. Risk of bias across studies.
Supplementary Table S6.2. Risk of bias table for all outcomes.

Supplementary Figure S4a. Evidence plot for pooled PFS (HR).
Supplementary Figure S4b. Evidence plot for BRAF subgroup (PFS-HR).
Supplementary Figure S5a. Evidence plot for pooled ORR (OR).
Supplementary Figure S5b. Evidence plot for BRAF subgroup (ORR-OR).
Supplementary Figure S6a. Evidence plot for pooled CRR (OR).
Supplementary Figure S6b. Evidence plot for BRAF subgroup (CRR-OR).
Supplementary Figure S7. Evidence plot for pooled TRAE (OR).
Supplementary Figure S8. Evidence plot for pooled SAE (OR).
Supplementary Figure S9. Evidence plot for pooled grade 3≤ AE (OR).
Supplementary Figure S10. Evidence plot for pooled TDR (OR).
Supplementary Figure S11. Evidence plot for PFS (HR).
Supplementary Figure S12. Evidence plot for ORR (OR).
Supplementary Figure S13. Evidence plot for CRR (OR).
Supplementary Figure S14. Evidence plot for TRAE (OR).
Supplementary Figure S15. Evidence plot for grade 3≤ AE (OR).
Supplementary Figure S16. Evidence plot for TDR (OR).
Supplementary Figure S17a. Funnel plot for pooled PFS (HR).
Supplementary Figure S17b. Funnel plot for BRAF subgroup (PFS-HR).
Supplementary Figure S18a. Funnel plot for pooled ORR (OR).
Supplementary Figure S18b. Funnel plot for BRAF subgroup (ORR-OR).
Supplementary Figure S19a. Funnel plot for pooled CRR (OR).
Supplementary Figure S19b. Funnel plot for BRAF subgroup (CRR-OR).
Supplementary Figure S20. Funnel plot for pooled TRAE (OR)
Supplementary Figure S21. Funnel plot for pooled SAE (OR)
Supplementary Figure S22. Funnel plot for pooled grade 3≤ AE (OR)
Supplementary Figure S23. Funnel plot for pooled TDR (OR)
Supplementary Figure S24. Funnel plot for PFS (HR)
Supplementary Figure S25. Funnel plot for ORR (OR)
Supplementary Figure S26. Funnel plot for CRR (OR)
Supplementary Figure S27. Funnel plot for TRAE (OR)
Supplementary Figure S28. Funnel plot for grade 3≤ AE (OR)
Supplementary Figure S29. Funnel plot for TDR (OR)
Supplementary Figure S30. Forest plot for pooled PFS (HR)
Supplementary Figure S31. Forest plot for pooled ORR (OR)
Supplementary Figure S32. Forest plot for pooled CRR (OR)
Supplementary Figure S33. Forest plot for pooled TRAE (OR)
Supplementary Figure S34. Forest plot for pooled SAE (OR)
Supplementary Figure S35. Forest plot for pooled grade 3≤ AE (OR)
Supplementary Figure S36. Forest plot for pooled TDR (OR)
Supplementary Figure S37. Forest plot for PFS (HR)
Supplementary Figure S38. Forest plot for ORR (OR)
Supplementary Figure S39. Forest plot for CRR (OR)
Supplementary Figure S40. Forest plot for TRAE (OR)
Supplementary Figure S41. Forest plot for grade 3≤ AE (OR)
Supplementary Figure S42. Forest plot for TDR (OR)
Supplementary Table S7. CINEMA analysis for ORR (OR)
Supplementary Table S8. CINEMA analysis for CRR (OR)
Supplementary Table S9. CINEMA analysis for TRAE (OR)
Supplementary Table S10. CINEMA analysis for SAE (OR)
Supplementary Table S11. CINEMA analysis for grade 3≤ AE (OR)

Supplementary Table S12. CINEMA analysis for TDR (OR)
Supplementary Table S13. GRADE assessment for PFS

Supplementary Table S1. Search terms and number of hits for the databases (date of search: 5th March 2023).

**PubMed (1311 hits), EMBASE (4559 hits), Cochrane Library (1775 hits):**
melanom* or melanocyt* AND (BRAF or BRAF* or MEK1 or MEK2 or MEK* or MAPK or ERK1 or ERK2 or ERK* or vemurafenib or zelboraf or dabrafenib or tanfilar or encorafenib or braftovi or trametinib or mekinist or cobimetinib or cotellic or binimetinib or mektovi or MEK162 or pd-1 or pd-l1 or pd-l2 or "programmed cell death receptor" or "programmed cell death 1 receptor" or "programmed cell death 2 receptor" or CLTA-4 or "Cytotoxic T-Lymphocyte-Associated Antigen 4" or "Cytotoxic T Lymphocyte Associated Antigen 4" or "Cytotoxic T-Lymphocyte Antigen 4" or "Cytotoxic T Lymphocyte Antigen 4" or CD152 or CD28 OR ipilimumab or MDX-CTLA-4 or Yervoy or tremelimumab or Imjuno or nivolumab or Opdivo or spartalizumab or pembrolizumab or Keytruda or atezolizumab or Tecentriq or durvalumab or Imfinzi or DTIC or dacarbazin or temozolomide or fotemustine or vindesine or paclitaxel or docetaxel or cabazitaxel or carboplatin or cisplatin or oxaliplatin or satraplatin or polyplatillen) AND (random*)
**Web of Science (1000 hits):**
ALL=((melanom* or melanocyt*) AND (BRAF or BRAF* or MEK1 or MEK2 or MEK* or MAPK or ERK1 or ERK2 or ERK* or vemurafenib or dabrafenib or encorafenib or trametinib or cobimetinib or binimetinib or MEK162 or pd-1 or pd-l1 or pd-l2 or "programmed cell death receptor" or CLTA-4 or "Cytotoxic T-Lymphocyte-Associated Antigen 4" or "Cytotoxic T Lymphocyte Associated Antigen 4" or CD152 or CD28 OR ipilimumab or MDX-CTLA-4 or tremelimumab or nivolumab or spartalizumab or pembrolizumab or atezolizumab or durvalumab or DTIC or dacarbazin or temozolomide or fotemustine or vindesine or paclitaxel or docetaxel or cabazitaxel or carboplatin or cisplatin or oxaliplatin or satraplatin or polyplatillen) AND (random*))

Supplementary Table S2. Characteristics of included studies in systematic review. Abbreviations: NIVO: Nivolumab; IPI: Ipilimumab; ORR: objective response rate; PFS: progression-free survival; OS: overall survival; AE: adverse event; TRAE: treatment-related adverse event.

Study	Purpose	Study period	Study type/ population	Intervention	Comparison/ Control	Endpoint	Summarized conclusion
CheckMate 511	Assess whether the combination of nivolumab 3 mg/kg with ipilimumab 1 mg/kg enhances the safety profile of the established dosing regimen for patients with advanced melanoma.	4 April 2016 – 27 March 2017	patients (N = 360) with previously untreated, unresectable stage III or IV melanoma, no prior systemic therapy for metastatic melanoma	nivolumab 3 mg/kg plus ipilimumab 1 mg/kg (NIVO3+IPI1)	nivolumab 1 mg/kg plus ipilimumab 3 mg/kg (NIVO1+IPI3)	Treatment-related grade 3-5 AEs	Significantly lower incidence of treatment-related grade 3 to 5 AEs on the NIVO3+IPI1 arm.
						PFS, OS	The survival analysis showed no meaningful difference in survival times.
CheckMate 064	Evaluating if the sequential application of nivolumab first, followed by ipilimumab, or the reversed sequence, could enhance safety without compromising effectiveness.	30 April 2013 – 21 July 2014	patients (N= 140) had unresectable stage III or stage IV melanoma, and were previously untreated or had progressed on previous systemic therapy	nivolumab 3 mg/kg followed by ipilimumab 3 mg/kg (N–I)	ipilimumab 3 mg/kg followed by nivolumab 3 mg/kg	Treatment-related grade 3–5 adverse events	The frequency and type of adverse events were similar.
						Overall response, OS	Nivolumab followed by ipilimumab was associated with a lower proportion of patients with disease progression, a higher proportion of patients with a confirmed response, and longer median overall survival.
Dreamseq	Identifying the most effective initial treatment or treatment sequence in advanced melanoma.	13 July 2015 – 16 July 2021	patients (N=265) with unresectable stage III or IV melanoma containing a BRAFV600E/K mutation.	Nivolumab/ipilimumab (A) followed by dabrafenib/trametinib (C)	Nivolumab/ipilimumab (A) followed by dabrafenib/trametinib (C)	OS, PFS, ORR	The recommended treatment sequence for most patients is starting with a combination of nivolumab and ipilimumab, followed by BRAF and MEK inhibitor therapy if deemed required.
						TRAE	Grade ≥ 3 toxicities occurred with similar frequency between arms.
Secombit	The lack of available prospective data on sequential immunotherapy and BRAF/MEK inhibition for BRAFV600-mutant metastatic melanoma.	November 2016 – May 2019	Patients (N=209) with untreated, metastatic BRAFV600-mutant melanoma	arm A: encorafenib plus binimetinib until progressive disease [PD] -> ipilimumab plus nivolumab		OS, PFS,safety	Patients with BRAFV600-mutant melanoma can experience clinically significant survival advantages through the sequential utilization of immunotherapy and targeted therapy.
				arm B: ipilimumab plus nivolumab until PD -> encorafenib plus binimetinib			
				arm C: encorafenib plus binimetinib -> ipilimumab plus nivolumab until PD -> encorafenib plus binimetinib			

Supplementary Figure S3. Comparative network plots for pooled and detailed analysis (a-b) ORR, CRR; (c-d) PFS; (e-f) OS; (g) TRAEs; (h) grade 3≤ AEs; (i) TDR.
Network plots for efficacy outcomes when NIBIT-M2 and NCT02374242 studies are excluded: (j-k) PFS; (l-m) OS; (n) ORR; (o) CRR.

a

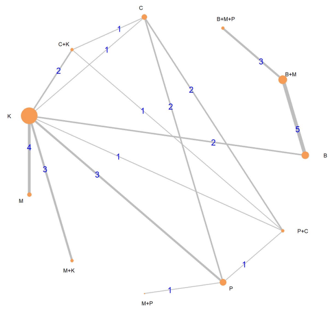

b

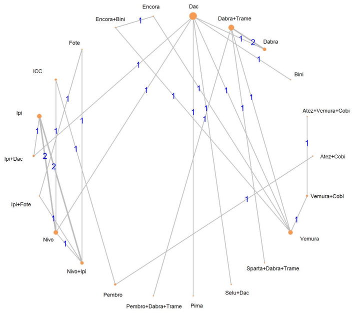

c

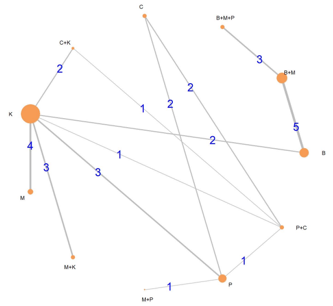

d

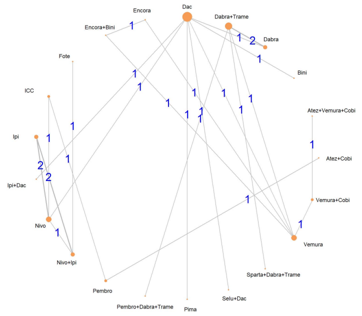

e

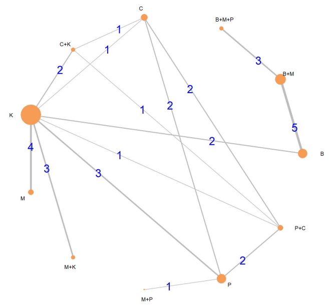

f

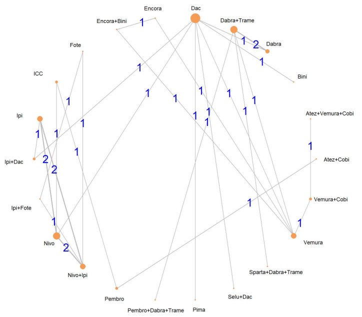

g

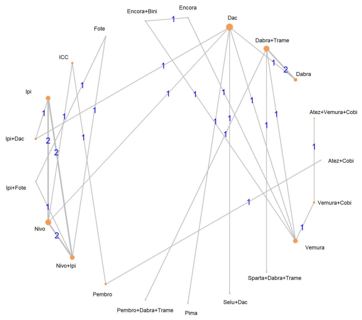

h

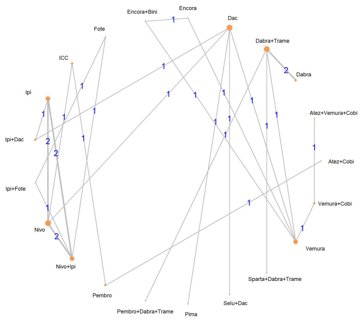

i

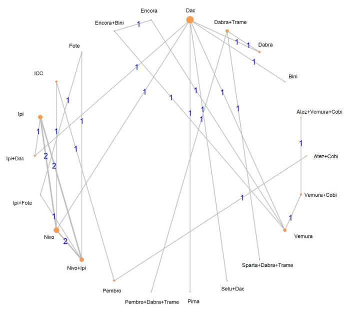
Encora
Dac
Encora+Bini
Dabra+Trame
Dabra
Fote
Bini
ICC
Ipi
Atez+Vemura+Cobi
Ipi+Dac
Atez+Cobi
Vemura+Cobi
Ipi+Fote
Nivo
Vemura
Sparta+Dabra+Trame
Nivo+Ipi
Selu+Dac
Pembro
Pembro+Dabra+Trame
Pima

j

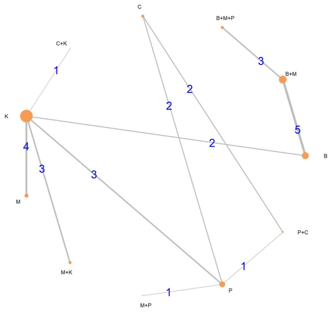
C
B+M+P
C+K
B+M
K
B
M
P+C
M+K
P
M+P

k

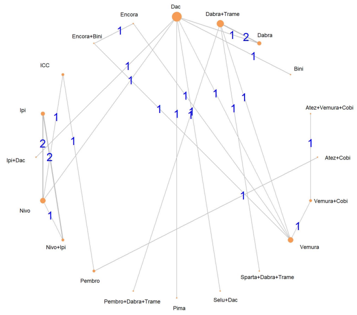
Dac
Encora
Dabra+Trame
Encora+Bini
Dabra
Bini
ICC
Atez+Vemura+Cobi
Ipi
Ipi+Dac
Atez+Cobi
Vemura+Cobi
Nivo
Nivo+Ipi
Vemura
Pembro
Sparta+Dabra+Trame
Pembro+Dabra+Trame
Selu+Dac
Pima

l

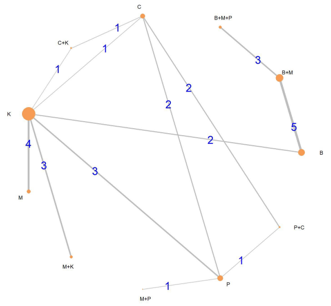
C
B+M+P
C+K
B+M
K
B
M
P+C
M+K
P
M+P

m

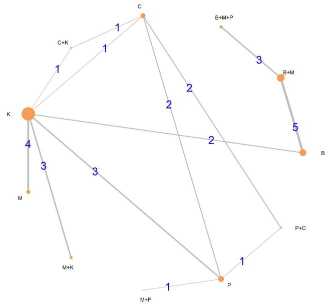
C
B+M+P
C+K
B+M
K
B
M
P+C
M+K
P
M+P

n

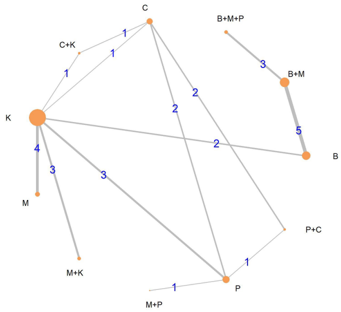
C
B+M+P
C+K
B+M
K
B
M
P+C
M+K
P
M+P

o

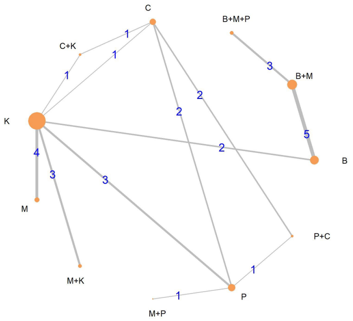
C
B+M+P
C+K
B+M
K
B
M
P+C
M+K
P
M+P

Supplementary Table S3. P-scores of detailed safety and pooled efficacy analysis.

ORR		CRR		PFS		OS		TRAE		Grade 3≤ AE	
**Treatment**	**P-score**	**Treatment**	**P-score**	**Treatment**	**P-score**	**Treatment**	**P-score**	**Treatment**	**P-score**	**Treatment**	**P-score**
Encora+Bini	0.9452	Nivo+Ipi	0.8886	Pembro+Dabra+Trame	0.9802	Pembro+Dabra+Trame	0.9011	Pembro	0.9569	Pembro	0.9932
Atez+Vemura+Cobi	0.9283	Nivo	0.8281	Atez+Vemura+Cobi	0.8855	Nivo+Ipi	0.8610	ICC	0.9296	ICC	0.9050
Vemura+Cobi	0.9064	Pembro	0.7731	Sparta+Dabra+Trame	0.8714	Pembro	0.8603	Dac	0.7842	Nivo	0.8940
Sparta+Dabra+Trame	0.8810	Atez+Cobi	0.7415	Encora+Bini	0.8020	Sparta+Dabra+Trame	0.8108	Nivo	0.7425	Dac	0.8741
Dabra+Trame	0.8029	Pembro+Dabra+Trame	0.6935	Dabra+Trame	0.7694	Atez+Cobi	0.7944	Ipi	0.7283	Ipi	0.8120
Encora	0.7742	Sparta+Dabra+Trame	0.6482	Vemura+Cobi	0.7662	Atez+Vemura+Cobi	0.7445	Encora+Bini	0.6883	Atez+Cobi	0.6961
Pembro+Dabra+Trame	0.7045	Dabra+Trame	0.5892	Pembro	0.7181	Nivo	0.6938	Dabra	0.6071	Dabra	0.6635
Vemura	0.6570	Encora+Bini	0.5835	Encora	0.6899	ICC	0.6350	Atez+Cobi	0.5702	Dabra+Trame	0.6299
Dabra	0.6276	Bini	0.5570	Atez+Cobi	0.6430	Encora+Bini	0.6245	Encora	0.5278	Selu+Dac	0.5955
Pembro	0.5714	Vemura+Cobi	0.5387	Dabra	0.5244	Dabra+Trame	0.6028	Dabra+Trame	0.5181	Ipi+Dac	0.5753
Nivo+Ipi	0.5220	Ipi+Dac	0.4985	Vemura	0.5206	Vemura+Cobi	0.5871	Selu+Dac	0.5092	Vemura	0.4815
Atez+Cobi	0.4960	Atez+Vemura+Cobi	0.4885	Nivo+Ipi	0.4938	Encora	0.5855	Pembro+Dabra+Trame	0.4546	Nivo+Ipi	0.4176
Nivo	0.4188	Ipi+Fote	0.4839	ICC	0.3869	Ipi+Dac	0.4230	Nivo+Ipi	0.4500	Encora	0.3976
Ipi+Fote	0.3485	Ipi	0.4785	Nivo	0.3731	Ipi	0.3242	Ipi+Dac	0.4199	Encora+Bini	0.3976
Bini	0.3300	Dabra	0.4335	Pima	0.2441	Dabra	0.3221	Ipi+Fote	0.4195	Sparta+Dabra+Trame	0.2861
Selu+Dac	0.2716	Vemura	0.3202	Bini	0.2252	Vemura	0.2626	Fote	0.3924	Pima	0.2162
Ipi+Dac	0.2506	Encora	0.3114	Selu+Dac	0.2186	Fote	0.2106	Vemura	0.2937	Vemura+Cobi	0.2091
ICC	0.1665	ICC	0.3037	Ipi	0.1570	Pima	0.2066	Vemura+Cobi	0.1756	Fote	0.1943
Ipi	0.1427	Fote	0.2898	Ipi+Dac	0.1410	Selu+Dac	0.1699	Pima	0.1395	Pembro+Dabra+Tram	0.1355
Pima	0.1084	Selu+Dac	0.2595	Fote	0.0539	Ipi+Fote	0.1590	Sparta+Dabra+Trame	0.1251	Atez+Vemura+Cobi	0.0927
Dac	0.1079	Dac	0.1668	Dac	0.0361	Bini	0.1188	Atez+Vemura+Cobi	0.0673	Ipi+Fote	0.0333
Fote	0.0386	Pima	0.1246			Dac	0.1024				

ORR		CRR		PFS		OS		TDR	
**Treatment**	**P-score**	**Treatment**	**P-score**	**Treatment**	**P-score**	**Treatment**	**P-score**	**Treatment**	**P-score**
B+M+P	0.9635	P+C	0.9126	B+M+P	0.9984	B+M+P	0.9652	Dabra	0.9210
B+M	0.9364	P	0.8074	B+M	0.9014	P+C	0.8738	Dac	0.9153
B	0.7969	M+P	0.7732	B	0.7861	B+M	0.7913	Fote	0.8308
P+C	0.7015	B+M+P	0.6746	P+C	0.7042	P	0.6605	Dabra+Trame	0.7946
P	0.5746	B+M	0.6351	P	0.5498	M+P	0.5833	Nivo	0.6990
M+P	0.4677	C+K	0.4551	M+P	0.4284	C+K	0.5036	Ipi	0.6579
M+K	0.3954	B	0.4001	M	0.3531	B	0.4390	Sparta+Dabra+Trame	0.6536
C+K	0.2830	C	0.3630	C+K	0.3156	C	0.2670	Ipi+Fote	0.6197
M	0.2708	M	0.2106	M+K	0.3086	M	0.1695	Selu+Dac	0.5986
C	0.0824	M+K	0.1478	C	0.0992	K	0.1398	Encora	0.5674
K	0.0277	K	0.1205	K	0.0552	M+K	0.1070	Encora+Bini	0.5674
								Bini	0.5629
								Vemura	0.5014
								Pembro+Dabra+Trame	0.4415
								ICC	0.4137
								Pembro	0.2734
								Atez+Vemura+Cobi	0.2395
								Nivo+Ipi	0.2131
								Vemura+Cobi	0.2029
								Ipi+Dac	0.1821
								Atez+Cobi	0.1016
								Pima	0.0423

Supplementary Table S4a. League tables of efficacy outcomes.

Objective response rate (OR, 95% CI)

B+M+P	1.06 [0.76; 1.47]	.	.	.	.	.	.	.	.	.
1.06 [0.76; 1.47]	B+M	1.99 [1.57; 2.52]	.	.	.	.	.	.	.	.
2.11 [1.41; 3.15]	1.99 [1.57; 2.52]	B	.	.	.	.	.	.	.	15.74 [8.58; 28.87]
4.41 [1.85; 10.53]	4.16 [1.86; 9.33]	2.09 [0.97; 4.53]	P+C	1.71 [1.07; 2.73]	.	.	2.54 [0.76; 8.48]	.	6.73 [4.29; 10.57]	37.90 [2.05; 701.88]
7.69 [3.44; 17.16]	7.26 [3.48; 15.12]	3.65 [1.82; 7.31]	1.74 [1.17; 2.60]	P	1.31 [0.76; 2.27]	.	.	.	3.58 [2.52; 5.08]	4.54 [3.04; 6.78]
10.10 [3.82; 26.72]	9.53 [3.81; 23.84]	4.80 [1.98; 11.62]	2.29 [1.16; 4.52]	1.31 [0.76; 2.27]	M+P	.	.	.	.	.
12.62 [4.83; 32.98]	11.92 [4.83; 29.40]	5.99 [2.51; 14.33]	2.86 [1.30; 6.29]	1.64 [0.81; 3.34]	1.25 [0.51; 3.07]	M+K	.	.	.	2.63 [1.40; 4.91]
17.04 [6.86; 42.30]	16.08 [6.88; 37.59]	8.09 [3.58; 18.28]	3.86 [2.02; 7.38]	2.22 [1.22; 4.04]	1.69 [0.75; 3.81]	1.35 [0.59; 3.09]	C+K	.	2.92 [0.51; 16.70]	1.72 [0.93; 3.20]
17.51 [7.57; 40.48]	16.53 [7.64; 35.77]	8.31 [3.99; 17.33]	3.97 [2.11; 7.47]	2.28 [1.33; 3.89]	1.73 [0.81; 3.73]	1.39 [0.66; 2.94]	1.03 [0.52; 2.04]	M	.	1.89 [1.25; 2.86]
29.33 [12.88; 66.81]	27.69 [13.01; 58.95]	13.93 [6.80; 28.54]	6.65 [4.46; 9.92]	3.82 [2.80; 5.20]	2.90 [1.55; 5.46]	2.32 [1.12; 4.84]	1.72 [0.93; 3.18]	1.68 [0.95; 2.94]	C	1.14 [0.62; 2.09]
33.15 [16.00; 68.69]	31.29 [16.31; 60.03]	15.74 [8.58; 28.87]	7.52 [4.67; 12.11]	4.31 [3.08; 6.04]	3.28 [1.72; 6.25]	2.63 [1.40; 4.91]	1.95 [1.13; 3.35]	1.89 [1.25; 2.86]	1.13 [0.77; 1.66]	K

Complete response rate (OR, 95% CI)

P+C	1.27 [0.86; 1.87]	.	.	.	5.14 [0.80; 33.21]	.	4.84 [2.87; 8.18]	.	.	14.21 [0.75; 267.52]
1.24 [0.85; 1.79]	P	1.14 [0.40; 3.20]	.	.	.	.	3.99 [2.59; 6.14]	.	.	14.02 [5.33; 36.87]
1.41 [0.47; 4.22]	1.14 [0.40; 3.20]	M+P	.	.	.	.	.	.	.	.
2.28 [0.33; 15.71]	1.85 [0.27; 12.48]	1.62 [0.18; 14.26]	B+M+P	1.06 [0.79; 1.43]	.	.	.	.	.	.
2.42 [0.36; 16.25]	1.95 [0.30; 12.91]	1.72 [0.20; 14.79]	1.06 [0.79; 1.43]	B+M	.	1.65 [1.27; 2.14]	.	.	.	.
4.14 [1.22; 14.06]	3.35 [0.98; 11.42]	2.94 [0.59; 14.64]	1.81 [0.21; 15.74]	1.71 [0.20; 14.56]	C+K	.	5.60 [0.26; 120.80]	.	.	2.17 [0.48; 9.84]
3.99 [0.60; 26.33]	3.22 [0.50; 20.92]	2.83 [0.33; 24.02]	1.75 [1.18; 2.59]	1.65 [1.27; 2.14]	0.96 [0.12; 8.07]	B	.	.	.	2.77 [0.48; 15.91]
5.61 [3.57; 8.82]	4.53 [3.02; 6.81]	3.99 [1.31; 12.10]	2.46 [0.36; 16.59]	2.32 [0.35; 15.31]	1.36 [0.40; 4.62]	1.41 [0.22; 9.12]	C	.	.	1.38 [0.55; 3.49]
8.96 [2.74; 29.33]	7.24 [2.28; 23.06]	6.37 [1.35; 30.07]	3.92 [0.52; 29.81]	3.71 [0.50; 27.55]	2.17 [0.47; 10.06]	2.25 [0.31; 16.43]	1.60 [0.50; 5.08]	M	.	1.23 [0.48; 3.18]
13.16 [2.00; 86.48]	10.64 [1.65; 68.70]	9.36 [1.11; 78.92]	5.76 [0.47; 70.22]	5.45 [0.46; 65.17]	3.18 [0.38; 26.51]	3.30 [0.28; 38.98]	2.35 [0.36; 15.14]	1.47 [0.20; 10.68]	M+K	0.84 [0.15; 4.79]
11.04 [5.42; 22.50]	8.93 [4.59; 17.34]	7.85 [2.30; 26.82]	4.84 [0.81; 29.03]	4.57 [0.78; 26.75]	2.67 [0.80; 8.93]	2.77 [0.48; 15.91]	1.97 [1.02; 3.81]	1.23 [0.48; 3.18]	0.84 [0.15; 4.79]	K

Progression-free survival (HR, 95% CI)

B+M+P	0.73 [0.55; 0.97]	.	.	.	.	.	.	.	.	.
0.73 [0.55; 0.97]	B+M	0.55 [0.45; 0.69]	.	.	.	.	.	.	.	.
0.41 [0.29; 0.58]	0.55 [0.45; 0.69]	B	.	.	.	.	.	.	.	0.27 [0.18; 0.4]
0.3 [0.15; 0.57]	0.4 [0.22; 0.73]	0.73 [0.41; 1.26]	P+C	0.79 [0.51; 1.23]	.	.	0.47 [0.22; 0.99]	.	0.4 [0.28; 0.57]	0.27 [0.12; 0.6]
0.21 [0.12; 0.38]	0.28 [0.17; 0.48]	0.51 [0.32; 0.81]	0.7 [0.5; 0.99]	P	0.87 [0.54; 1.4]	.	.	.	0.55 [0.4; 0.75]	0.55 [0.42; 0.73]
0.18 [0.08; 0.39]	0.25 [0.12; 0.5]	0.44 [0.23; 0.87]	0.61 [0.34; 1.11]	0.87 [0.54; 1.4]	M+P	.	.	.	.	.
0.16 [0.09; 0.29]	0.22 [0.13; 0.36]	0.39 [0.25; 0.63]	0.54 [0.34; 0.86]	0.77 [0.54; 1.11]	0.89 [0.49; 1.62]	M	.	.	.	0.7 [0.54; 0.89]
0.15 [0.08; 0.29]	0.21 [0.12; 0.37]	0.38 [0.22; 0.64]	0.52 [0.32; 0.84]	0.74 [0.48; 1.14]	0.85 [0.44; 1.62]	0.95 [0.62; 1.48]	C+K	.	.	0.7 [0.48; 1.02]
0.15 [0.08; 0.29]	0.21 [0.12; 0.36]	0.37 [0.22; 0.63]	0.51 [0.3; 0.87]	0.73 [0.47; 1.13]	0.84 [0.44; 1.62]	0.95 [0.62; 1.45]	0.99 [0.6; 1.65]	M+K	.	0.73 [0.52; 1.04]
0.12 [0.06; 0.22]	0.16 [0.09; 0.28]	0.28 [0.17; 0.49]	0.39 [0.28; 0.54]	0.55 [0.41; 0.75]	0.64 [0.36; 1.13]	0.72 [0.46; 1.13]	0.76 [0.46; 1.23]	0.76 [0.45; 1.27]	C	.
0.11 [0.07; 0.19]	0.15 [0.1; 0.24]	0.27 [0.18; 0.4]	0.38 [0.25; 0.56]	0.54 [0.41; 0.7]	0.62 [0.36; 1.06]	0.7 [0.54; 0.89]	0.73 [0.5; 1.05]	0.73 [0.52; 1.04]	0.96 [0.66; 1.4]	K

Overall survival (HR, 95% CI)

B+M+P	.	0.79 [0.64; 0.98]	.	.	.	.	.	.	.	.
0.85 [0.55; 1.31]	P+C	.	0.84 [0.64; 1.11]	.	0.41 [0.21; 0.81]	.	0.55 [0.44; 0.7]	.	0.44 [0.22; 0.89]	.
0.79 [0.64; 0.98]	0.92 [0.63; 1.35]	B+M	.	.	.	0.69 [0.59; 0.8]	.	.	.	.
0.67 [0.45; 0.99]	0.79 [0.63; 0.98]	0.85 [0.61; 1.19]	P	0.94 [0.59; 1.49]	.	.	0.68 [0.57; 0.81]	.	0.68 [0.56; 0.82]	.
0.63 [0.35; 1.16]	0.74 [0.44; 1.23]	0.8 [0.46; 1.42]	0.94 [0.59; 1.49]	M+P	.	.	.	.	.	.
0.58 [0.38; 0.88]	0.68 [0.5; 0.92]	0.73 [0.51; 1.05]	0.86 [0.66; 1.12]	0.91 [0.54; 1.54]	C+K	.	0.8 [0.47; 1.38]	.	0.74 [0.58; 0.94]	.
0.54 [0.42; 0.7]	0.64 [0.45; 0.9]	0.69 [0.59; 0.8]	0.81 [0.61; 1.08]	0.86 [0.5; 1.48]	0.94 [0.68; 1.31]	B	.	.	0.79 [0.63; 1.01]	.
0.47 [0.32; 0.7]	0.55 [0.45; 0.68]	0.6 [0.43; 0.84]	0.7 [0.6; 0.83]	0.75 [0.46; 1.21]	0.82 [0.63; 1.06]	0.87 [0.64; 1.17]	C	.	0.88 [0.64; 1.2]	.
0.44 [0.3; 0.66]	0.52 [0.38; 0.7]	0.56 [0.4; 0.78]	0.66 [0.51; 0.84]	0.69 [0.41; 1.17]	0.76 [0.57; 1.02]	0.81 [0.59; 1.09]	0.93 [0.72; 1.21]	M	0.99 [0.83; 1.19]	.
0.44 [0.3; 0.62]	0.51 [0.4; 0.66]	0.55 [0.41; 0.73]	0.65 [0.55; 0.76]	0.68 [0.42; 1.12]	0.76 [0.61; 0.94]	0.79 [0.63; 1.01]	0.92 [0.76; 1.11]	0.99 [0.83; 1.19]	K	0.95 [0.73; 1.25]
0.41 [0.26; 0.64]	0.48 [0.34; 0.7]	0.52 [0.35; 0.77]	0.61 [0.45; 0.84]	0.65 [0.37; 1.14]	0.72 [0.51; 1.01]	0.76 [0.53; 1.08]	0.87 [0.63; 1.21]	0.94 [0.68; 1.3]	0.95 [0.73; 1.25]	M+K

Objective response rate (OR, 95% CI)

[illegible]

Complete response rate (OR, 95% CI)

[illegible]

Progression-free survival (HR, 95% CI)

[illegible]

Overall survival (HR, 95% CI)

[illegible]

Supplementary Table S4b. League tables of efficacy outcomes excluding NIBIT-M2 and NCT02374242 studies.

**Progression-free survival (HR 95% CI)**																			
B+M+P	0.73 [0.55; 0.98]	.	.	.	.	.	.	.	.	.									
0.73 [0.55; 0.98]	B+M	0.55 [0.45; 0.69]	.	.	.	.	.	.	.	.									
0.41 [0.28; 0.58]	0.55 [0.45; 0.69]	B	.	.	.	.	.	.	.	0.27 [0.18; 0.41]									
0.27 [0.13; 0.55]	0.37 [0.19; 0.71]	0.66 [0.35; 1.23]	P+C	0.79 [0.5; 1.25]	.	.	.	.	0.4 [0.28; 0.57]	.									
0.2 [0.11; 0.37]	0.28 [0.16; 0.47]	0.49 [0.3; 0.8]	0.75 [0.51; 1.11]	P	0.87 [0.53; 1.42]	.	.	.	0.55 [0.4; 0.76]	0.55 [0.42; 0.73]									
0.17 [0.08; 0.38]	0.24 [0.12; 0.49]	0.43 [0.21; 0.85]	0.65 [0.35; 1.21]	0.87 [0.53; 1.42]	M+P	.	.	.	.	.									
0.16 [0.09; 0.29]	0.22 [0.13; 0.37]	0.39 [0.25; 0.63]	0.59 [0.35; 1.03]	0.8 [0.55; 1.16]	0.92 [0.5; 1.72]	M	.	.	.	0.69 [0.54; 0.89]									
0.15 [0.08; 0.29]	0.21 [0.12; 0.37]	0.37 [0.22; 0.63]	0.57 [0.31; 1.03]	0.76 [0.48; 1.19]	0.87 [0.44; 1.7]	0.94 [0.61; 1.46]	M+K	.	.	0.73 [0.52; 1.04]									
0.15 [0.07; 0.3]	0.2 [0.11; 0.38]	0.36 [0.2; 0.66]	0.54 [0.28; 1.06]	0.73 [0.43; 1.25]	0.84 [0.41; 1.73]	0.91 [0.54; 1.54]	0.96 [0.54; 1.72]	C+K	.	0.76 [0.48; 1.2]									
0.11 [0.06; 0.21]	0.15 [0.08; 0.28]	0.27 [0.15; 0.48]	0.4 [0.29; 0.57]	0.54 [0.4; 0.74]	0.63 [0.35; 1.12]	0.68 [0.41; 1.11]	0.72 [0.41; 1.25]	0.74 [0.4; 1.38]	C	.									
0.11 [0.07; 0.19]	0.15 [0.1; 0.24]	0.27 [0.18; 0.41]	0.41 [0.26; 0.67]	0.55 [0.42; 0.73]	0.64 [0.36; 1.13]	0.69 [0.54; 0.89]	0.73 [0.52; 1.04]	0.76 [0.48; 1.2]	1.02 [0.67; 1.55]	K									
Pembro+Dabra+Trame	.	.	.	0.53 [0.32; 0.88]	.	.	.	.	.	.	.	.	.	.	.	.	.	.	.
0.67 [0.32; 1.4]	Atez+Vemura+Cobi	.	.	.	0.79 [0.58; 1.08]	.	.	.	.	.	.	.	.	.	.	.	.	.	.
0.64 [0.35; 1.17]	0.97 [0.52; 1.82]	Sparta+Dabra+Trame	.	0.82 [0.59; 1.13]	.	.	.	.	.	.	.	.	.	.	.	.	.	.	.
0.55 [0.28; 1.09]	0.83 [0.47; 1.48]	0.86 [0.49; 1.49]	Encora+Bini	.	.	.	0.83 [0.58; 1.17]	.	.	0.55 [0.39; 0.78]	.	.	.	.	.	.	.	.	.
0.53 [0.32; 0.88]	0.79 [0.46; 1.36]	0.82 [0.59; 1.13]	0.96 [0.61; 1.51]	Dabra+Trame	.	.	.	.	0.59 [0.45; 0.78]	0.56 [0.41; 0.76]	.	.	.	.	.	.	.	.	.
0.53 [0.27; 1.03]	0.79 [0.58; 1.08]	0.82 [0.47; 1.4]	0.95 [0.59; 1.54]	1 [0.64; 1.54]	Vemura+Cobi	.	.	.	.	0.58 [0.42; 0.8]	.	.	.	.	.	.	.	.	.
0.45 [0.18; 1.15]	0.68 [0.28; 1.65]	0.7 [0.3; 1.65]	0.82 [0.36; 1.88]	0.86 [0.39; 1.88]	0.86 [0.38; 1.95]	Pembro	.	0.87 [0.61; 1.25]	.	.	.	0.47 [0.3; 0.73]	.	.	.	.	.	.	.
0.45 [0.23; 0.9]	0.68 [0.38; 1.21]	0.7 [0.4; 1.22]	0.82 [0.58; 1.16]	0.85 [0.54; 1.35]	0.86 [0.53; 1.39]	1 [0.43; 2.29]	Encora	.	.	0.68 [0.48; 0.97]	.	.	.	.	.	.	.	.	.
0.39 [0.15; 1.07]	0.59 [0.23; 1.54]	0.61 [0.24; 1.54]	0.71 [0.29; 1.77]	0.75 [0.31; 1.77]	0.75 [0.3; 1.84]	0.87 [0.61; 1.25]	0.87 [0.35; 2.16]	Atez+Cobi	.	.	.	.	.	.	.	.	.	.	.
0.31 [0.17; 0.54]	0.46 [0.26; 0.82]	0.48 [0.32; 0.72]	0.55 [0.34; 0.91]	0.58 [0.45; 0.75]	0.58 [0.36; 0.94]	0.68 [0.31; 1.49]	0.68 [0.41; 1.12]	0.78 [0.33; 1.86]	Dabra	.	.	.	.	.	.	.	.	.	0.3 [0.17; 0.54]
0.31 [0.17; 0.55]	0.46 [0.29; 0.73]	0.47 [0.31; 0.73]	0.55 [0.39; 0.79]	0.58 [0.43; 0.77]	0.58 [0.42; 0.8]	0.68 [0.32; 1.43]	0.68 [0.48; 0.96]	0.78 [0.34; 1.79]	1 [0.7; 1.42]	Vemura	.	.	.	.	.	.	.	.	0.26 [0.18; 0.37]
0.28 [0.13; 0.59]	0.41 [0.2; 0.84]	0.43 [0.22; 0.82]	0.5 [0.26; 0.94]	0.52 [0.29; 0.92]	0.52 [0.28; 0.97]	0.61 [0.31; 1.17]	0.61 [0.32; 1.15]	0.7 [0.33; 1.48]	0.9 [0.5; 1.6]	0.9 [0.53; 1.54]	Nivo+Ipi	.	0.79 [0.58; 1.08]	.	.	.	0.4 [0.31; 0.53]	.	.
0.21 [0.09; 0.49]	0.32 [0.15; 0.68]	0.33 [0.16; 0.68]	0.39 [0.19; 0.78]	0.4 [0.21; 0.77]	0.4 [0.2; 0.81]	0.47 [0.3; 0.73]	0.47 [0.23; 0.96]	0.54 [0.31; 0.95]	0.69 [0.36; 1.34]	0.7 [0.38; 1.28]	0.77 [0.47; 1.27]	ICC	0.97 [0.64; 1.46]	.	.	.	.	.	.
0.21 [0.1; 0.42]	0.31 [0.16; 0.59]	0.32 [0.18; 0.58]	0.38 [0.21; 0.66]	0.39 [0.24; 0.64]	0.39 [0.22; 0.69]	0.46 [0.25; 0.84]	0.46 [0.26; 0.82]	0.53 [0.26; 1.06]	0.68 [0.4; 1.13]	0.68 [0.43; 1.07]	0.75 [0.57; 0.99]	0.97 [0.64; 1.46]	Nivo	.	.	.	0.55 [0.44; 0.68]	.	0.4 [0.29; 0.55]
0.14 [0.07; 0.3]	0.21 [0.1; 0.42]	0.22 [0.11; 0.42]	0.25 [0.13; 0.48]	0.26 [0.15; 0.47]	0.27 [0.14; 0.5]	0.31 [0.14; 0.69]	0.31 [0.17; 0.58]	0.36 [0.15; 0.86]	0.46 [0.26; 0.82]	0.46 [0.27; 0.78]	0.51 [0.28; 0.92]	0.66 [0.34; 1.28]	0.68 [0.4; 1.15]	Pima	.	.	.	.	0.59 [0.39; 0.9]
0.13 [0.06; 0.28]	0.2 [0.1; 0.39]	0.21 [0.11; 0.38]	0.24 [0.13; 0.44]	0.25 [0.15; 0.43]	0.25 [0.14; 0.45]	0.3 [0.14; 0.64]	0.3 [0.16; 0.54]	0.34 [0.14; 0.79]	0.44 [0.25; 0.75]	0.44 [0.27; 0.71]	0.49 [0.28; 0.85]	0.63 [0.33; 1.19]	0.64 [0.39; 1.05]	0.95 [0.54; 1.67]	Bini	.	.	.	0.62 [0.43; 0.9]
0.13 [0.06; 0.28]	0.2 [0.1; 0.39]	0.2 [0.11; 0.38]	0.24 [0.13; 0.44]	0.25 [0.15; 0.42]	0.25 [0.14; 0.45]	0.29 [0.13; 0.63]	0.29 [0.16; 0.53]	0.33 [0.14; 0.79]	0.43 [0.25; 0.74]	0.43 [0.26; 0.7]	0.48 [0.27; 0.84]	0.62 [0.32; 1.17]	0.64 [0.39; 1.04]	0.93 [0.53; 1.65]	0.98 [0.58; 1.67]	Selu+Dac	.	.	0.63 [0.43; 0.92]
0.11 [0.05; 0.24]	0.17 [0.09; 0.33]	0.18 [0.09; 0.33]	0.2 [0.11; 0.38]	0.21 [0.12; 0.37]	0.21 [0.12; 0.39]	0.25 [0.13; 0.47]	0.25 [0.14; 0.46]	0.29 [0.14; 0.59]	0.37 [0.21; 0.64]	0.37 [0.22; 0.61]	0.41 [0.32; 0.53]	0.53 [0.34; 0.84]	0.54 [0.44; 0.67]	0.8 [0.45; 1.42]	0.84 [0.5; 1.43]	0.86 [0.5; 1.48]	Ipi	.	.
0.11 [0.05; 0.22]	0.16 [0.09; 0.31]	0.17 [0.09; 0.3]	0.2 [0.11; 0.35]	0.21 [0.13; 0.34]	0.21 [0.12; 0.36]	0.24 [0.11; 0.51]	0.24 [0.14; 0.42]	0.28 [0.12; 0.64]	0.35 [0.21; 0.59]	0.36 [0.23; 0.55]	0.39 [0.23; 0.67]	0.51 [0.28; 0.94]	0.53 [0.34; 0.83]	0.78 [0.46; 1.31]	0.82 [0.51; 1.32]	0.83 [0.51; 1.35]	0.96 [0.59; 1.58]	Ipi+Dac	0.76 [0.56; 1.03]
0.08 [0.04; 0.16]	0.12 [0.07; 0.22]	0.13 [0.08; 0.21]	0.15 [0.09; 0.24]	0.16 [0.11; 0.23]	0.16 [0.1; 0.25]	0.18 [0.09; 0.36]	0.18 [0.11; 0.3]	0.21 [0.1; 0.45]	0.27 [0.18; 0.4]	0.27 [0.2; 0.37]	0.3 [0.2; 0.46]	0.39 [0.23; 0.66]	0.4 [0.29; 0.55]	0.59 [0.39; 0.9]	0.62 [0.43; 0.9]	0.63 [0.43; 0.92]	0.73 [0.5; 1.08]	0.76 [0.56; 1.03]	Dac
**Overall survival (HR 95% CI)**																			
B+M+P	.	0.79 [0.64; 0.98]	.	.	.	.	.	.	.	.									
0.81 [0.52; 1.27]	P+C	.	0.84 [0.63; 1.12]	.	.	.	0.55 [0.44; 0.7]	.	.	.									
0.79 [0.64; 0.98]	0.97 [0.65; 1.45]	B+M	.	.	.	0.69 [0.59; 0.8]	.	.	.	.									
0.66 [0.45; 0.98]	0.82 [0.64; 1.04]	0.84 [0.61; 1.16]	P	.	0.94 [0.59; 1.49]	.	0.68 [0.57; 0.81]	.	0.68 [0.56; 0.82]	.									
0.62 [0.41; 0.95]	0.76 [0.54; 1.08]	0.79 [0.54; 1.14]	0.94 [0.71; 1.23]	C+K	.	.	0.8 [0.47; 1.38]	.	0.69 [0.53; 0.9]	.									
0.63 [0.34; 1.14]	0.77 [0.46; 1.3]	0.79 [0.45; 1.39]	0.94 [0.59; 1.49]	1.01 [0.59; 1.72]	M+P	.	.	.	.	.									
0.54 [0.42; 0.7]	0.67 [0.47; 0.97]	0.69 [0.59; 0.8]	0.83 [0.61; 1.11]	0.88 [0.63; 1.23]	0.87 [0.51; 1.51]	B	.	.	0.79 [0.63; 1.01]	.									
0.47 [0.31; 0.7]	0.57 [0.46; 0.72]	0.59 [0.42; 0.84]	0.7 [0.6; 0.83]	0.75 [0.57; 0.99]	0.75 [0.46; 1.21]	0.85 [0.63; 1.16]	C	.	0.88 [0.64; 1.2]	.									
0.44 [0.3; 0.66]	0.54 [0.39; 0.76]	0.56 [0.4; 0.78]	0.66 [0.52; 0.85]	0.71 [0.52; 0.96]	0.7 [0.42; 1.19]	0.81 [0.59; 1.09]	0.94 [0.73; 1.23]	M	0.99 [0.83; 1.19]	.									
0.44 [0.3; 0.62]	0.54 [0.41; 0.7]	0.55 [0.41; 0.73]	0.66 [0.56; 0.78]	0.7 [0.55; 0.89]	0.7 [0.43; 1.14]	0.79 [0.63; 1.01]	0.93 [0.77; 1.13]	0.99 [0.83; 1.19]	K	0.95 [0.73; 1.25]									
0.41 [0.26; 0.64]	0.51 [0.35; 0.75]	0.52 [0.35; 0.77]	0.63 [0.45; 0.85]	0.66 [0.46; 0.95]	0.66 [0.38; 1.15]	0.76 [0.53; 1.08]	0.89 [0.64; 1.23]	0.94 [0.68; 1.3]	0.95 [0.73; 1.25]	M+K									
Pembro+Dabra+Trame	.	.	.	.	.	.	.	.	0.64 [0.39; 1.06]	.	.	.	.	.	.	.	.	.	.
0.86 [0.45; 1.63]	Nivo+Ipi	.	.	.	.	0.84 [0.67; 1.05]	.	.	.	.	.	.	0.54 [0.45; 0.65]	.	.	.	.	.	.
0.9 [0.41; 1.95]	1.05 [1.6; 1.77]	Pembro	.	0.94 [0.62; 1.45]	.	.	0.74 [0.5; 1.11]	.	.	.	.	.	.	.	.	.	.	.	.
0.81 [0.45; 1.45]	0.94 [0.58; 1.54]	0.9 [0.47; 1.73]	Sparta+Dabra+Trame	.	.	.	.	.	0.79 [0.59; 1.05]	.	.	.	.	.	.	.	.	.	.
0.85 [0.35; 2.05]	0.99 [0.51; 1.93]	0.94 [0.62; 1.45]	1.05 [2.1; 2.29]	Atez+Cobi	.	.	.	.	.	.	.	.	.	.	.	.	.	.	.
0.74 [0.38; 1.45]	0.87 [0.53; 1.43]	0.83 [0.42; 1.62]	0.91 [0.54; 1.54]	0.88 [0.39; 1.93]	Atez+Vemura+Cobi	.	.	.	.	0.85 [0.64; 1.12]	.	.	.	.	.	.	.	.	.
0.7 [0.38; 1.3]	0.82 [0.67; 1]	0.78 [0.48; 1.26]	0.87 [0.55; 1.36]	0.83 [0.44; 1.57]	0.94 [0.59; 1.51]	Nivo	0.95 [0.73; 1.23]	.	.	.	.	.	0.68 [0.59; 0.77]	.	.	.	.	.	0.5 [0.4; 0.63]
0.66 [0.34; 1.3]	0.78 [0.56; 1.08]	0.74 [0.5; 1.11]	0.82 [0.49; 1.39]	0.79 [0.44; 1.4]	0.9 [0.53; 1.54]	0.95 [0.73; 1.23]	ICC	.	.	.	.	.	.	.	.	.	.	.	.
0.65 [0.36; 1.2]	0.76 [0.5; 1.15]	0.73 [0.39; 1.32]	0.8 [0.52; 1.25]	0.76 [0.36; 1.6]	0.88 [0.57; 1.35]	0.93 [0.64; 1.35]	0.98 [0.62; 1.54]	Encora+Bini	.	.	0.97 [0.76; 1.25]	.	.	.	0.68 [0.53; 0.85]	.	.	.	.
0.64 [0.39; 1.06]	0.75 [0.5; 1.12]	0.71 [0.39; 1.28]	0.79 [0.59; 1.05]	0.76 [0.36; 1.57]	0.86 [0.56; 1.34]	0.91 [0.64; 1.3]	0.96 [0.62; 1.49]	0.98 [0.7; 1.38]	Dabra+Trame	.	.	.	.	0.74 [0.58; 0.94]	0.69 [0.53; 0.9]	.	.	.	.
0.63 [0.34; 1.16]	0.73 [0.49; 1.12]	0.7 [0.38; 1.28]	0.78 [0.5; 1.21]	0.74 [0.35; 1.57]	0.85 [0.64; 1.12]	0.9 [0.62; 1.31]	0.95 [0.6; 1.51]	0.97 [0.69; 1.36]	0.99 [0.7; 1.38]	Vemura+Cobi	.	.	.	.	0.7 [0.55; 0.89]	.	.	.	.
0.63 [0.34; 1.15]	0.73 [0.48; 1.12]	0.7 [0.38; 1.28]	0.78 [0.5; 1.21]	0.74 [0.35; 1.55]	0.84 [0.54; 1.31]	0.9 [0.61; 1.31]	0.94 [0.59; 1.49]	0.96 [0.75; 1.23]	0.98 [0.7; 1.38]	0.99 [0.7; 1.4]	Encora	.	.	.	0.71 [0.55; 0.9]	.	.	.	.
0.53 [0.29; 0.97]	0.62 [0.45; 0.84]	0.59 [0.34; 1.01]	0.65 [0.42; 1.01]	0.62 [0.31; 1.23]	0.71 [0.45; 1.12]	0.76 [0.58; 0.97]	0.79 [0.55; 1.15]	0.81 [0.57; 1.16]	0.83 [0.59; 1.15]	0.84 [0.58; 1.2]	0.84 [0.58; 1.21]	Ipi+Dac	0.8 [0.48; 1.34]	.	.	.	.	.	0.69 [0.57; 0.84]
0.47 [0.25; 0.88]	0.55 [0.46; 0.66]	0.53 [0.32; 0.86]	0.58 [0.37; 0.93]	0.56 [0.29; 1.07]	0.64 [0.39; 1.03]	0.68 [0.59; 0.76]	0.71 [0.53; 0.95]	0.73 [0.49; 1.07]	0.74 [0.51; 1.06]	0.75 [0.51; 1.11]	0.76 [0.51; 1.12]	0.9 [0.68; 1.17]	Ipi	.	.	.	.	.	.
0.47 [0.27; 0.82]	0.55 [0.36; 0.84]	0.53 [0.29; 0.96]	0.58 [0.41; 0.84]	0.55 [0.27; 1.16]	0.64 [0.4; 1.02]	0.68 [0.47; 0.98]	0.71 [0.45; 1.12]	0.73 [0.5; 1.06]	0.74 [0.59; 0.92]	0.75 [0.51; 1.09]	0.76 [0.52; 1.11]	0.9 [0.63; 1.28]	1 [0.68; 1.48]	Dabra	.	.	.	.	0.76 [0.48; 1.2]
0.44 [0.25; 0.77]	0.52 [0.36; 0.73]	0.49 [0.28; 0.86]	0.54 [0.38; 0.79]	0.52 [0.26; 1.05]	0.59 [0.41; 0.86]	0.63 [0.47; 0.84]	0.66 [0.45; 0.98]	0.68 [0.54; 0.86]	0.69 [0.54; 0.88]	0.7 [0.55; 0.89]	0.7 [0.55; 0.9]	0.84 [0.64; 1.09]	0.93 [0.68; 1.27]	0.93 [0.7; 1.25]	Vemura	.	.	.	0.81 [0.66; 1]
0.4 [0.2; 0.8]	0.47 [0.29; 0.76]	0.45 [0.23; 0.85]	0.5 [0.29; 0.86]	0.47 [0.22; 1.03]	0.54 [0.31; 0.95]	0.58 [0.37; 0.89]	0.61 [0.36; 1]	0.62 [0.38; 1.01]	0.63 [0.39; 1.01]	0.64 [0.39; 1.04]	0.64 [0.39; 1.05]	0.76 [0.5; 1.16]	0.85 [0.54; 1.32]	0.85 [0.52; 1.39]	0.91 [0.59; 1.39]	Pima	.	.	0.89 [0.61; 1.3]
0.39 [0.2; 0.75]	0.45 [0.3; 0.68]	0.43 [0.23; 0.79]	0.48 [0.29; 0.79]	0.45 [0.22; 0.95]	0.52 [0.31; 0.88]	0.55 [0.38; 0.8]	0.58 [0.36; 0.92]	0.59 [0.38; 0.92]	0.6 [0.39; 0.92]	0.61 [0.39; 0.95]	0.61 [0.39; 0.96]	0.73 [0.51; 1.05]	0.81 [0.55; 1.21]	0.81 [0.52; 1.27]	0.87 [0.6; 1.27]	0.96 [0.58; 1.57]	Selu+Dac	.	0.93 [0.68; 1.28]
0.36 [0.19; 0.68]	0.42 [0.28; 0.63]	0.4 [0.22; 0.72]	0.44 [0.27; 0.73]	0.42 [0.2; 0.88]	0.48 [0.29; 0.8]	0.51 [0.36; 0.73]	0.54 [0.35; 0.84]	0.55 [0.36; 0.84]	0.56 [0.38; 0.84]	0.57 [0.37; 0.87]	0.57 [0.37; 0.87]	0.68 [0.48; 0.95]	0.76 [0.52; 1.09]	0.76 [0.5; 1.15]	0.81 [0.57; 1.15]	0.89 [0.55; 1.43]	0.93 [0.61; 1.43]	Bini	1 [0.75; 1.34]
0.36 [0.2; 0.64]	0.42 [0.32; 0.55]	0.4 [0.24; 0.67]	0.44 [0.3; 0.66]	0.42 [0.21; 0.83]	0.48 [0.32; 0.73]	0.51 [0.41; 0.63]	0.54 [0.38; 0.76]	0.55 [0.4; 0.75]	0.56 [0.42; 0.74]	0.57 [0.41; 0.78]	0.57 [0.41; 0.78]	0.68 [0.57; 0.82]	0.76 [0.6; 0.96]	0.76 [0.56; 1.03]	0.81 [0.66; 0.99]	0.89 [0.61; 1.3]	0.93 [0.68; 1.28]	1 [0.75; 1.34]	Dac
**Objective response rate (OR 95% CI)**																			
B+M+P	1.06 [0.77; 1.47]	.	.	.	.	.	.	.	.	.									
1.06 [0.77; 1.47]	B+M	1.99 [1.57; 2.52]	.	.	.	.	.	.	.	.									
2.11 [1.41; 3.15]	1.99 [1.57; 2.52]	B	.	.	.	.	.	.	.	15.74 [8.59; 28.85]									
4.25 [1.75; 10.32]	4.01 [1.76; 9.16]	2.02 [0.92; 4.45]	P+C	1.71 [1.08; 2.72]	.	.	.	.	6.73 [4.29; 10.54]	.									
7.59 [3.40; 16.97]	7.16 [3.43; 14.95]	3.60 [1.80; 7.23]	1.79 [1.18; 2.70]	P	1.31 [0.76; 2.27]	.	.	.	3.58 [2.52; 5.07]	4.54 [3.04; 6.77]									
9.97 [3.77; 26.39]	9.41 [3.76; 23.54]	4.73 [1.95; 11.48]	2.35 [1.18; 4.66]	1.31 [0.76; 2.27]	M+P	.	.	.	.	.									
12.63 [4.84; 32.94]	11.92 [4.84; 29.36]	6.00 [2.51; 14.31]	2.97 [1.33; 6.64]	1.66 [0.82; 3.39]	1.27 [0.52; 3.11]	M+K	.	.	.	2.63 [1.41; 4.90]									
17.50 [7.58; 40.40]	16.52 [7.64; 35.70]	8.31 [3.99; 17.31]	4.12 [2.14; 7.93]	2.31 [1.35; 3.95]	1.76 [0.81; 3.78]	1.39 [0.66; 2.93]	M	.	.	1.89 [1.25; 2.86]									
19.48 [7.61; 49.87]	18.38 [7.61; 44.41]	9.25 [3.95; 21.63]	4.58 [2.13; 9.84]	2.57 [1.31; 5.03]	1.95 [0.82; 4.65]	1.54 [0.65; 3.66]	1.11 [0.54; 2.30]	C+K	2.92 [0.51; 16.69]	1.56 [0.83; 2.93]									
28.96 [12.69; 66.07]	27.33 [12.80; 58.31]	13.75 [6.69; 28.25]	6.81 [4.51; 10.28]	3.81 [2.80; 5.19]	2.90 [1.55; 5.45]	2.29 [1.10; 4.79]	1.65 [0.94; 2.92]	1.49 [0.75; 2.95]	C	1.14 [0.62; 2.09]									
33.15 [16.02; 68.60]	31.28 [16.33; 59.95]	15.74 [8.59; 28.85]	7.80 [4.69; 12.96]	4.37 [3.10; 6.16]	3.32 [1.74; 6.35]	2.63 [1.41; 4.90]	1.89 [1.25; 2.86]	1.70 [0.94; 3.09]	1.14 [0.78; 1.69]	K									
**Complete response rate (OR 95% CI)**																			
P+C	1.27 [0.86; 1.87]	.	.	.	.	.	4.84 [2.87; 8.18]	.	.	.									
1.23 [0.84; 1.78]	P	1.14 [0.40; 3.20]	.	.	.	.	3.99 [2.59; 6.14]	.	.	14.02 [5.33; 36.87]									
1.39 [0.46; 4.18]	1.14 [0.40; 3.20]	M+P	.	.	.	.	.	.	.	.									
2.19 [0.31; 15.33]	1.79 [0.26; 12.22]	1.57 [0.18; 13.94]	B+M+P	1.06 [0.79; 1.43]	.	.	.	.	.	.									
2.32 [0.34; 15.86]	1.89 [0.28; 12.63]	1.67 [0.19; 14.46]	1.06 [0.79; 1.43]	B+M	.	1.65 [1.27; 2.14]	.	.	.	.									
3.52 [0.70; 17.81]	2.87 [0.58; 14.18]	2.53 [0.38; 16.92]	1.61 [0.16; 16.64]	1.52 [0.15; 15.42]	C+K	.	5.60 [0.26; 120.80]	.	.	2.03 [0.37; 11.20]									
3.83 [0.57; 25.70]	3.12 [0.48; 20.48]	2.75 [0.32; 23.48]	1.75 [1.18; 2.59]	1.65 [1.27; 2.14]	1.09 [0.11; 10.88]	B	.	.	.	2.77 [0.48; 15.91]									
5.53 [3.49; 8.76]	4.51 [3.00; 6.78]	3.97 [1.31; 12.05]	2.52 [0.37; 17.17]	2.38 [0.36; 15.85]	1.57 [0.32; 7.62]	1.45 [0.22; 9.44]	C	.	.	1.38 [0.55; 3.49]									
8.61 [2.56; 28.89]	7.02 [2.17; 22.70]	6.17 [1.29; 29.49]	3.92 [0.52; 29.81]	3.71 [0.50; 27.55]	2.44 [0.41; 14.41]	2.25 [0.31; 16.43]	1.56 [0.48; 5.00]	M	.	1.23 [0.48; 3.18]									
12.64 [1.89; 84.41]	10.31 [1.58; 67.24]	9.07 [1.07; 77.15]	5.76 [0.47; 70.22]	5.45 [0.46; 65.17]	3.59 [0.36; 35.76]	3.30 [0.28; 38.98]	2.28 [0.35; 14.85]	1.47 [0.20; 10.68]	M+K	0.84 [0.15; 4.79]									
10.60 [4.99; 22.53]	8.65 [4.33; 17.28]	7.61 [2.19; 26.38]	4.84 [0.81; 29.03]	4.57 [0.78; 26.75]	3.01 [0.67; 13.49]	2.77 [0.48; 15.91]	1.92 [0.97; 3.79]	1.23 [0.48; 3.18]	0.84 [0.15; 4.79]	K									

Supplementary Table S5. League table of safety outcomes. **Grade 3≤ AE:** OR for Nivo+Ipi was 0.41 (95% CI 0.13-1.23) and for Dabra+Trame was 0.24 (95% CI 0.11-0.53) versus Pembro+Dabra+Trame intervention. Although no significant difference was seen in survival outcomes between Encora+Bini and Dabra+Trame (OS HR 0.98 95% CI 0.7-1.38; PFS HR 0.96 95% CI 0.61-1.51); in terms of grade 3-5 adverse events Dabra+Trame reached significantly lower risk (OR 0.56; 95% CI 0.32-0.99) compared to Encora+Bini. **TDR:** OR for Dabra+Trame group was 0.29 (95% CI 0.13-0.64) versus Pembro+Dabra+Trame and 0.45 (95% CI 0.05-3.94) for Pembro+Dabra+Trame compared to Nivo+Ipi treatment.

[illegible]elated adverse events (OR, 95% CI)

Pembro	0.76 [0.29; 1.95]	.	.	.	.	.	0.08 [0.03; 0.23]	.	.	.	.	.	.	.	.	.	.	.	.	.
0.76 [0.29; 1.95]	ICC	.	0.18 [0.03; 1.22]	.	.	.	.	.	.	.	.	.	.	.	.	.	.	.	.	.
0.16 [0.02; 1.58]	0.21 [0.03; 1.70]	Dac	0.94 [0.37; 2.37]	.	.	0.61 [0.23; 1.64]	.	.	.	0.33 [0.01; 9.26]	.	.	0.19 [0.04; 0.85]	.	.	0.08 [0.01; 0.40]	.	0.03 [0.00; 0.55]	.	.
0.14 [0.02; 1.15]	0.18 [0.03; 1.22]	0.86 [0.37; 2.01]	Nivo	1.18 [0.63; 2.22]	.	.	.	.	.	.	.	0.21 [0.08; 0.54]	.	.	.	.	.	.	.	.
0.13 [0.01; 1.19]	0.18 [0.02; 1.27]	0.82 [0.31; 2.19]	0.95 [0.53; 1.73]	Ipi	.	.	.	.	.	.	.	0.43 [0.18; 1.02]	0.37 [0.08; 1.67]	.	.	.	.	.	.	.
0.15 [0.00; 8.38]	0.19 [0.00; 9.90]	0.90 [0.03; 25.63]	1.04 [0.03; 33.19]	1.09 [0.03; 35.98]	Encora+Bini	.	.	0.42 [0.05; 3.40]	.	.	.	.	.	.	.	0.15 [0.01; 3.14]	.	.	.	.
0.08 [0.01; 0.96]	0.11 [0.01; 1.05]	0.51 [0.20; 1.27]	0.59 [0.17; 2.07]	0.62 [0.16; 2.37]	0.56 [0.02; 16.57]	Dabra	.	.	1.01 [0.25; 4.09]	.	.	.	.	.	.	.	.	.	.	.
0.08 [0.03; 0.23]	0.10 [0.02; 0.44]	0.48 [0.04; 5.97]	0.55 [0.05; 6.00]	0.58 [0.05; 6.76]	0.53 [0.01; 35.44]	0.94 [0.06; 13.89]	Atez+Cobi	.	.	.	.	.	.	.	.	.	.	.	.	.
0.06 [0.00; 4.24]	0.08 [0.00; 5.03]	0.38 [0.01; 13.43]	0.44 [0.01; 17.28]	0.46 [0.01; 18.70]	0.42 [0.05; 3.40]	0.75 [0.02; 27.17]	0.80 [0.01; 63.35]	Encora	.	.	.	.	.	.	.	0.34 [0.01; 9.32]	.	.	.	.
0.06 [0.00; 0.80]	0.07 [0.01; 0.89]	0.35 [0.09; 1.35]	0.41 [0.08; 2.01]	0.43 [0.08; 2.27]	0.39 [0.01; 10.79]	0.69 [0.21; 2.32]	0.73 [0.04; 12.97]	0.92 [0.03; 31.49]	Dabra+Trame	.	0.74 [0.13; 4.18]	.	.	.	.	0.57 [0.13; 2.48]	.	.	0.11 [0.03; 0.40]	.
0.05 [0.00; 3.04]	0.07 [0.00; 3.59]	0.33 [0.01; 9.26]	0.39 [0.01; 12.00]	0.41 [0.01; 13.01]	0.37 [0.00; 41.84]	0.66 [0.02; 20.75]	0.70 [0.01; 45.78]	0.88 [0.01; 114.89]	0.95 [0.03; 34.58]	Selu+Dac	.	.	.	.	.	.	.	.	.	.
0.04 [0.00; 0.99]	0.06 [0.00; 1.13]	0.26 [0.03; 2.33]	0.30 [0.03; 3.17]	0.31 [0.03; 3.50]	0.29 [0.01; 12.17]	0.51 [0.06; 4.22]	0.54 [0.02; 15.51]	0.68 [0.01; 34.73]	0.74 [0.13; 4.18]	0.77 [0.01; 41.62]	Pembro+Dabra+Trame	.	.	.	.	.	.	.	.	.
0.04 [0.00; 0.43]	0.06 [0.01; 0.47]	0.28 [0.09; 0.88]	0.32 [0.14; 0.73]	0.34 [0.15; 0.75]	0.31 [0.01; 10.72]	0.55 [0.12; 2.40]	0.58 [0.05; 7.24]	0.73 [0.02; 30.90]	0.79 [0.13; 4.70]	0.83 [0.02; 28.02]	1.08 [0.09; 12.92]	Nivo+Ipi	.	0.83 [0.18; 3.94]	0.74 [0.15; 3.72]	.	.	.	.	.
0.04 [0.00; 0.45]	0.05 [0.01; 0.49]	0.24 [0.08; 0.78]	0.28 [0.09; 0.93]	0.30 [0.09; 0.95]	0.27 [0.01; 9.44]	0.48 [0.11; 2.12]	0.51 [0.04; 7.34]	0.64 [0.02; 27.21]	0.70 [0.12; 4.15]	0.73 [0.02; 24.69]	0.95 [0.08; 11.39]	0.88 [0.23; 3.43]	Ipi+Dac	.	.	.	.	.	.	.
0.04 [0.00; 0.58]	0.05 [0.00; 0.65]	0.23 [0.03; 1.60]	0.27 [0.05; 1.55]	0.28 [0.05; 1.61]	0.26 [0.01; 12.36]	0.46 [0.05; 3.88]	0.49 [0.03; 9.37]	0.61 [0.01; 35.06]	0.66 [0.06; 7.01]	0.69 [0.01; 32.39]	0.90 [0.05; 16.80]	0.83 [0.18; 3.94]	0.95 [0.12; 7.47]	Ipi+Fote	0.88 [0.17; 4.64]	.	.	.	.	.
0.03 [0.00; 0.54]	0.04 [0.00; 0.60]	0.20 [0.03; 1.49]	0.24 [0.04; 1.46]	0.25 [0.04; 1.51]	0.23 [0.00; 11.23]	0.40 [0.04; 3.61]	0.43 [0.02; 8.58]	0.54 [0.01; 31.82]	0.58 [0.05; 6.48]	0.61 [0.01; 29.43]	0.79 [0.04; 15.40]	0.74 [0.15; 3.72]	0.84 [0.10; 6.95]	0.88 [0.17; 4.64]	Fote	.	.	.	.	.
0.02 [0.00; 0.30]	0.03 [0.00; 0.33]	0.13 [0.03; 0.50]	0.15 [0.03; 0.74]	0.16 [0.03; 0.83]	0.15 [0.01; 3.14]	0.26 [0.06; 1.04]	0.27 [0.02; 4.79]	0.34 [0.01; 9.32]	0.37 [0.11; 1.30]	0.39 [0.01; 14.05]	0.51 [0.06; 4.30]	0.47 [0.08; 2.75]	0.53 [0.09; 3.14]	0.56 [0.05; 5.93]	0.64 [0.06; 7.01]	Vemura	0.39 [0.06; 2.44]	.	.	.
0.01 [0.00; 0.21]	0.01 [0.00; 0.24]	0.05 [0.01; 0.49]	0.06 [0.01; 0.67]	0.06 [0.01; 0.73]	0.06 [0.00; 2.03]	0.10 [0.01; 1.01]	0.11 [0.00; 3.20]	0.13 [0.00; 5.84]	0.15 [0.02; 1.34]	0.15 [0.00; 8.52]	0.20 [0.01; 3.30]	0.18 [0.01; 2.34]	0.21 [0.02; 2.67]	0.22 [0.01; 4.34]	0.25 [0.01; 5.09]	0.39 [0.06; 2.44]	Vemura+Cobi	.	.	0.17 [0.01; 3.70]
0.00 [0.00; 0.19]	0.01 [0.00; 0.23]	0.03 [0.00; 0.55]	0.03 [0.00; 0.72]	0.03 [0.00; 0.79]	0.03 [0.00; 2.78]	0.06 [0.00; 1.26]	0.06 [0.00; 2.94]	0.07 [0.00; 7.68]	0.08 [0.00; 2.12]	0.08 [0.00; 7.30]	0.11 [0.00; 4.43]	0.10 [0.00; 2.48]	0.11 [0.00; 2.82]	0.12 [0.00; 4.25]	0.14 [0.00; 4.95]	0.21 [0.01; 5.66]	0.55 [0.01; 23.38]	Pima	.	.
0.01 [0.00; 0.12]	0.01 [0.00; 0.13]	0.04 [0.01; 0.25]	0.04 [0.01; 0.34]	0.05 [0.01; 0.38]	0.04 [0.00; 1.48]	0.07 [0.01; 0.44]	0.08 [0.00; 1.84]	0.10 [0.00; 4.26]	0.11 [0.03; 0.40]	0.11 [0.00; 5.11]	0.14 [0.02; 1.28]	0.13 [0.01; 1.23]	0.15 [0.02; 1.41]	0.16 [0.01; 2.41]	0.18 [0.01; 2.84]	0.29 [0.05; 1.76]	0.73 [0.06; 9.69]	1.34 [0.04; 45.88]	Sparta+Dabra+Trame	.
0.00 [0.00; 0.12]	0.00 [0.00; 0.14]	0.01 [0.00; 0.40]	0.01 [0.00; 0.51]	0.01 [0.00; 0.55]	0.01 [0.00; 1.09]	0.02 [0.00; 0.80]	0.02 [0.00; 1.79]	0.02 [0.00; 2.98]	0.02 [0.00; 1.10]	0.03 [0.00; 4.13]	0.03 [0.00; 2.18]	0.03 [0.00; 1.70]	0.04 [0.00; 1.94]	0.04 [0.00; 2.73]	0.04 [0.00; 3.16]	0.07 [0.00; 2.39]	0.17 [0.01; 3.70]	0.31 [0.00; 39.88]	0.23 [0.00; 12.95]	Atez+Vemura+Cobi

Grade ≥3 adverse events (OR, 95% CI)

Pembro	0.56 [0.33; 0.97]	.	.	.	0.25 [0.16; 0.38]	.	.	.	.	.	.	.	.	.	.	.	.	.	.	.
0.56 [0.33; 0.97]	ICC	0.93 [0.57; 1.50]	.	.	.	.	.	.	.	.	.	.	.	.	.	.	.	.	.	.
0.52 [0.25; 1.08]	0.93 [0.57; 1.50]	Nivo	0.90 [0.52; 1.54]	0.80 [0.61; 1.07]	.	.	.	.	.	.	0.20 [0.14; 0.28]	.	.	.	.	.	.	.	.	.
0.50 [0.21; 1.20]	0.88 [0.44; 1.76]	0.95 [0.58; 1.57]	Dac	.	.	.	.	0.34 [0.14; 0.82]	0.29 [0.20; 0.44]	0.24 [0.16; 0.35]	.	.	.	.	0.12 [0.06; 0.24]	.	.	.	.	.
0.41 [0.19; 0.90]	0.74 [0.42; 1.28]	0.79 [0.60; 1.04]	0.84 [0.48; 1.45]	Ipi	.	.	.	.	0.50 [0.14; 1.71]	.	0.25 [0.18; 0.36]	.	.	.	.	.	.	.	.	.
0.25 [0.16; 0.38]	0.44 [0.22; 0.88]	0.48 [0.21; 1.10]	0.50 [0.19; 1.33]	0.60 [0.25; 1.45]	Atez+Cobi	.	.	.	.	.	.	.	.	.	.	.	.	.	.	.
0.19 [0.07; 0.57]	0.34 [0.14; 0.88]	0.37 [0.17; 0.83]	0.39 [0.21; 0.73]	0.47 [0.20; 1.08]	0.78 [0.24; 2.50]	Dabra	0.90 [0.63; 1.30]	.	.	.	.	.	.	.	.	.	.	.	.	.
0.18 [0.06; 0.48]	0.31 [0.13; 0.73]	0.34 [0.17; 0.68]	0.35 [0.21; 0.59]	0.42 [0.20; 0.89]	0.71 [0.23; 2.12]	0.90 [0.63; 1.30]	Dabra+Trame	.	.	0.68 [0.48; 0.95]	.	.	.	0.41 [0.28; 0.61]	.	.	.	0.24 [0.11; 0.53]	.	.
0.17 [0.05; 0.59]	0.30 [0.10; 0.92]	0.32 [0.12; 0.89]	0.34 [0.14; 0.82]	0.41 [0.14; 1.15]	0.68 [0.18; 2.54]	0.87 [0.30; 2.56]	0.96 [0.35; 2.66]	Selu+Dac	.	.	.	.	.	.	.	.	.	.	.	.
0.15 [0.06; 0.39]	0.27 [0.12; 0.58]	0.29 [0.16; 0.52]	0.30 [0.21; 0.45]	0.36 [0.19; 0.68]	0.61 [0.22; 1.70]	0.78 [0.37; 1.62]	0.86 [0.46; 1.63]	0.89 [0.34; 2.34]	Ipi+Dac	.	.	.	.	.	.	.	.	.	.	.
0.12 [0.05; 0.31]	0.21 [0.10; 0.47]	0.23 [0.12; 0.43]	0.24 [0.16; 0.35]	0.29 [0.15; 0.56]	0.48 [0.17; 1.37]	0.61 [0.37; 1.01]	0.68 [0.48; 0.95]	0.70 [0.27; 1.83]	0.79 [0.46; 1.35]	Vemura	.	0.83 [0.52; 1.30]	0.83 [0.52; 1.30]	.	.	0.48 [0.31; 0.73]	.	.	.	.
0.10 [0.05; 0.23]	0.18 [0.10; 0.33]	0.20 [0.14; 0.27]	0.21 [0.12; 0.37]	0.25 [0.18; 0.34]	0.41 [0.17; 1.02]	0.53 [0.22; 1.25]	0.59 [0.27; 1.27]	0.61 [0.21; 1.75]	0.68 [0.35; 1.32]	0.86 [0.43; 1.74]	Nivo+Ipi	.	.	.	.	.	0.46 [0.14; 1.48]	.	.	0.19 [0.06; 0.61]
0.10 [0.03; 0.28]	0.17 [0.07; 0.43]	0.19 [0.09; 0.41]	0.20 [0.11; 0.36]	0.24 [0.11; 0.53]	0.40 [0.13; 1.24]	0.51 [0.26; 0.99]	0.56 [0.32; 0.99]	0.58 [0.20; 1.68]	0.65 [0.32; 1.32]	0.83 [0.52; 1.30]	0.95 [0.41; 2.20]	Encora	1.00 [0.63; 1.59]	.	.	.	.	.	.	.
0.10 [0.03; 0.28]	0.17 [0.07; 0.43]	0.19 [0.09; 0.41]	0.20 [0.11; 0.36]	0.24 [0.11; 0.53]	0.40 [0.13; 1.24]	0.51 [0.26; 0.99]	0.56 [0.32; 0.99]	0.58 [0.20; 1.68]	0.65 [0.32; 1.32]	0.83 [0.52; 1.30]	0.95 [0.41; 2.20]	1.00 [0.63; 1.59]	Encora+Bini	.	.	.	.	.	.	.
0.07 [0.02; 0.22]	0.13 [0.05; 0.33]	0.14 [0.06; 0.31]	0.15 [0.08; 0.28]	0.18 [0.08; 0.41]	0.29 [0.09; 0.94]	0.37 [0.22; 0.64]	0.41 [0.28; 0.61]	0.43 [0.15; 1.27]	0.48 [0.23; 1.01]	0.61 [0.37; 1.02]	0.71 [0.30; 1.68]	0.74 [0.37; 1.47]	0.74 [0.37; 1.47]	Sparta+Dabra+Trame	.	.	.	.	.	.
0.06 [0.02; 0.18]	0.10 [0.04; 0.28]	0.11 [0.05; 0.26]	0.12 [0.06; 0.24]	0.14 [0.06; 0.34]	0.23 [0.07; 0.78]	0.30 [0.12; 0.76]	0.33 [0.14; 0.78]	0.34 [0.11; 1.05]	0.38 [0.17; 0.85]	0.48 [0.22; 1.08]	0.56 [0.22; 1.40]	0.59 [0.23; 1.48]	0.59 [0.23; 1.48]	0.79 [0.31; 2.05]	Pima	.	.	.	.	.
0.06 [0.02; 0.16]	0.10 [0.04; 0.25]	0.11 [0.05; 0.23]	0.12 [0.07; 0.20]	0.14 [0.06; 0.30]	0.23 [0.07; 0.71]	0.29 [0.15; 0.57]	0.33 [0.19; 0.56]	0.34 [0.12; 0.96]	0.38 [0.19; 0.75]	0.48 [0.31; 0.73]	0.55 [0.24; 1.26]	0.58 [0.31; 1.09]	0.58 [0.31; 1.09]	0.78 [0.40; 1.53]	0.99 [0.40; 2.46]	Vemura+Cobi	.	.	0.64 [0.42; 0.97]	.
0.05 [0.01; 0.19]	0.08 [0.02; 0.31]	0.09 [0.03; 0.31]	0.10 [0.03; 0.35]	0.11 [0.03; 0.38]	0.19 [0.04; 0.84]	0.24 [0.06; 1.04]	0.27 [0.07; 1.10]	0.28 [0.06; 1.36]	0.31 [0.08; 1.20]	0.40 [0.10; 1.55]	0.46 [0.14; 1.48]	0.48 [0.11; 2.03]	0.48 [0.11; 2.03]	0.65 [0.15; 2.79]	0.82 [0.18; 3.65]	0.83 [0.20; 3.46]	Fote	.	.	0.41 [0.13; 1.32]
0.04 [0.01; 0.15]	0.07 [0.02; 0.24]	0.08 [0.03; 0.23]	0.08 [0.03; 0.22]	0.10 [0.03; 0.30]	0.17 [0.04; 0.65]	0.22 [0.09; 0.52]	0.24 [0.11; 0.53]	0.25 [0.07; 0.90]	0.28 [0.10; 0.76]	0.35 [0.15; 0.83]	0.41 [0.13; 1.23]	0.43 [0.16; 1.13]	0.43 [0.16; 1.13]	0.57 [0.24; 1.39]	0.73 [0.22; 2.36]	0.73 [0.28; 1.91]	0.88 [0.18; 4.44]	Pembro+Dabra+Trame	.	.
0.04 [0.01; 0.11]	0.07 [0.02; 0.17]	0.07 [0.03; 0.17]	0.07 [0.04; 0.15]	0.09 [0.04; 0.22]	0.15 [0.04; 0.49]	0.19 [0.09; 0.41]	0.21 [0.11; 0.41]	0.22 [0.07; 0.67]	0.24 [0.11; 0.54]	0.31 [0.17; 0.56]	0.36 [0.14; 0.89]	0.37 [0.18; 0.79]	0.37 [0.18; 0.79]	0.50 [0.23; 1.10]	0.64 [0.23; 1.73]	0.64 [0.42; 0.97]	0.78 [0.18; 3.44]	0.88 [0.31; 2.50]	Atez+Vemura+Cobi	.
0.02 [0.00; 0.08]	0.03 [0.01; 0.13]	0.04 [0.01; 0.13]	0.04 [0.01; 0.15]	0.05 [0.01; 0.16]	0.08 [0.02; 0.34]	0.10 [0.02; 0.43]	0.11 [0.03; 0.45]	0.11 [0.02; 0.56]	0.13 [0.03; 0.50]	0.16 [0.04; 0.64]	0.19 [0.06; 0.61]	0.20 [0.05; 0.83]	0.20 [0.05; 0.83]	0.26 [0.06; 1.15]	0.33 [0.07; 1.50]	0.34 [0.08; 1.42]	0.41 [0.13; 1.32]	0.46 [0.09; 2.33]	0.52 [0.12; 2.35]	Ipi+Fote

Therapy discontinuation rate (OR, 95% CI)

Dabra	0.84 [0.16; 4.43]	.	0.56 [0.27; 1.14]	.	.	.	.	.	.	.	.	.	.	.	.	.	.	.	.	.	.
0.84 [0.16; 4.43]	Dac	.	.	0.40 [0.17; 0.94]	.	.	.	0.30 [0.10; 0.93]	.	.	0.26 [0.13; 0.55]	0.22 [0.08; 0.58]	.	.	.	.	.	.	0.07 [0.04; 0.15]	.	0.01 [0.00; 0.1[illegible]
0.79 [0.05; 13.83]	0.94 [0.09; 9.67]	Fote	.	.	.	.	0.35 [0.03; 3.61]	.	.	.	.	.	.	.	.	.	0.09 [0.01; 0.79]	.	.	.	.
0.56 [0.27; 1.14]	0.67 [0.11; 4.08]	0.71 [0.04; 13.47]	Dabra+Trame	.	.	0.61 [0.34; 1.09]	.	.	.	.	.	.	0.29 [0.13; 0.64]	.	.	.	.	.	.	.	.
0.32 [0.05; 2.05]	0.39 [0.17; 0.86]	0.41 [0.05; 3.64]	0.58 [0.08; 4.20]	Nivo	0.93 [0.66; 1.30]	.	.	.	.	.	.	.	.	0.42 [0.18; 0.97]	.	.	0.21 [0.14; 0.31]	.	.	.	.
0.30 [0.05; 1.92]	0.35 [0.15; 0.83]	0.37 [0.04; 3.32]	0.53 [0.07; 3.93]	0.92 [0.66; 1.28]	Ipi	.	.	.	.	.	.	.	.	.	.	.	0.26 [0.18; 0.37]	.	0.28 [0.03; 2.83]	.	.
0.34 [0.14; 0.86]	0.41 [0.06; 2.73]	0.43 [0.02; 8.73]	0.61 [0.34; 1.09]	1.06 [0.13; 8.34]	1.16 [0.14; 9.29]	Sparta+Dabra+Trame	.	.	.	.	.	.	.	.	.	.	.	.	.	.	.
0.28 [0.03; 2.95]	0.33 [0.06; 1.78]	0.35 [0.03; 3.61]	0.49 [0.04; 5.87]	0.85 [0.19; 3.78]	0.93 [0.21; 4.11]	0.81 [0.06; 10.24]	Ipi+Fote	.	.	.	.	.	.	.	.	.	0.26 [0.06; 1.11]	.	.	.	.
0.25 [0.03; 1.87]	0.30 [0.10; 0.93]	0.32 [0.02; 4.20]	0.45 [0.05; 3.79]	0.77 [0.19; 3.10]	0.84 [0.20; 3.49]	0.73 [0.08; 6.68]	0.91 [0.12; 6.91]	Selu+Dac	.	.	.	.	.	.	.	.	.	.	.	.	.
0.22 [0.03; 1.60]	0.26 [0.08; 0.79]	0.27 [0.02; 3.61]	0.39 [0.05; 3.25]	0.67 [0.17; 2.65]	0.73 [0.18; 2.98]	0.63 [0.07; 5.73]	0.78 [0.10; 5.92]	0.86 [0.18; 4.24]	Encora	1.00 [0.57; 1.75]	.	0.86 [0.49; 1.48]	.	.	.	.	.	.	.	.	.
0.22 [0.03; 1.60]	0.26 [0.08; 0.79]	0.27 [0.02; 3.61]	0.39 [0.05; 3.25]	0.67 [0.17; 2.65]	0.73 [0.18; 2.98]	0.63 [0.07; 5.73]	0.78 [0.10; 5.92]	0.86 [0.18; 4.24]	1.00 [0.57; 1.75]	Encora+Bini	.	0.86 [0.49; 1.48]	.	.	.	.	.	.	.	.	.
0.22 [0.04; 1.36]	0.26 [0.13; 0.55]	0.28 [0.02; 3.21]	0.40 [0.06; 2.80]	0.68 [0.23; 2.03]	0.75 [0.24; 2.31]	0.65 [0.08; 4.96]	0.80 [0.13; 5.05]	0.88 [0.23; 3.41]	1.02 [0.27; 3.90]	1.02 [0.27; 3.90]	Bini	.	.	.	.	.	.	.	.	.	.
0.18 [0.03; 1.27]	0.22 [0.08; 0.58]	0.23 [0.02; 2.91]	0.33 [0.04; 2.59]	0.57 [0.16; 2.02]	0.62 [0.17; 2.28]	0.54 [0.06; 4.58]	0.67 [0.10; 4.70]	0.74 [0.17; 3.30]	0.86 [0.49; 1.48]	0.86 [0.49; 1.48]	0.84 [0.25; 2.83]	Vemura	.	.	.	.	.	0.38 [0.23; 0.61]	.	.	.
0.16 [0.05; 0.47]	0.19 [0.03; 1.39]	0.20 [0.01; 4.29]	0.29 [0.13; 0.64]	0.49 [0.06; 4.20]	0.54 [0.06; 4.68]	0.47 [0.17; 1.26]	0.58 [0.04; 7.83]	0.64 [0.06; 6.28]	0.74 [0.08; 7.21]	0.74 [0.08; 7.21]	0.72 [0.09; 5.99]	0.86 [0.09; 7.87]	Pembro+Dabra+Trame	.	.	.	.	.	.	.	.
0.14 [0.02; 1.04]	0.16 [0.05; 0.52]	0.17 [0.02; 1.79]	0.24 [0.03; 2.10]	0.42 [0.18; 0.97]	0.46 [0.19; 1.13]	0.40 [0.04; 3.69]	0.49 [0.09; 2.73]	0.55 [0.11; 2.76]	0.63 [0.13; 3.16]	0.63 [0.13; 3.16]	0.62 [0.16; 2.43]	0.74 [0.16; 3.35]	0.85 [0.09; 8.51]	ICC	0.61 [0.26; 1.43]	.	.	.	.	.	.
0.08 [0.01; 0.75]	0.10 [0.02; 0.42]	0.10 [0.01; 1.26]	0.15 [0.01; 1.50]	0.26 [0.08; 0.85]	0.28 [0.08; 0.97]	0.24 [0.02; 2.63]	0.30 [0.04; 2.03]	0.33 [0.05; 2.07]	0.38 [0.06; 2.37]	0.38 [0.06; 2.37]	0.37 [0.07; 1.89]	0.45 [0.08; 2.55]	0.52 [0.04; 6.03]	0.61 [0.26; 1.43]	Pembro	.	.	.	.	0.44 [0.22; 0.89]	.
0.08 [0.01; 0.60]	0.09 [0.03; 0.30]	0.10 [0.01; 1.33]	0.14 [0.02; 1.21]	0.24 [0.06; 1.00]	0.26 [0.06; 1.13]	0.23 [0.02; 2.12]	0.28 [0.04; 2.20]	0.31 [0.06; 1.60]	0.36 [0.15; 0.86]	0.36 [0.15; 0.86]	0.35 [0.09; 1.41]	0.42 [0.21; 0.82]	0.48 [0.05; 4.90]	0.57 [0.11; 2.98]	0.93 [0.14; 6.04]	Atez+Vemura+Cobi	.	0.90 [0.56; 1.46]	.	.	.
0.07 [0.01; 0.47]	0.09 [0.04; 0.21]	0.09 [0.01; 0.79]	0.13 [0.02; 0.96]	0.22 [0.16; 0.32]	0.24 [0.17; 0.34]	0.21 [0.03; 1.70]	0.26 [0.06; 1.11]	0.29 [0.07; 1.20]	0.33 [0.08; 1.37]	0.33 [0.08; 1.37]	0.33 [0.10; 1.02]	0.39 [0.11; 1.44]	0.45 [0.05; 3.94]	0.53 [0.21; 1.31]	0.87 [0.25; 3.03]	0.93 [0.21; 4.05]	Nivo+Ipi	.	.	.	.
0.07 [0.01; 0.51]	0.08 [0.03; 0.25]	0.09 [0.01; 1.15]	0.12 [0.02; 1.03]	0.22 [0.06; 0.83]	0.24 [0.06; 0.94]	0.20 [0.02; 1.82]	0.25 [0.03; 1.88]	0.28 [0.06; 1.34]	0.32 [0.16; 0.67]	0.32 [0.16; 0.67]	0.32 [0.09; 1.17]	0.38 [0.23; 0.61]	0.44 [0.05; 4.19]	0.51 [0.10; 2.50]	0.84 [0.14; 5.11]	0.90 [0.56; 1.46]	0.97 [0.24; 3.89]	Vemura+Cobi	.	.	.
0.06 [0.01; 0.38]	0.08 [0.04; 0.15]	0.08 [0.01; 0.88]	0.11 [0.02; 0.78]	0.20 [0.07; 0.53]	0.21 [0.08; 0.59]	0.18 [0.02; 1.38]	0.23 [0.04; 1.36]	0.25 [0.07; 0.94]	0.29 [0.08; 1.07]	0.29 [0.08; 1.07]	0.29 [0.11; 0.77]	0.34 [0.11; 1.11]	0.40 [0.05; 3.21]	0.46 [0.13; 1.70]	0.76 [0.16; 3.61]	0.82 [0.21; 3.18]	0.88 [0.31; 2.49]	0.91 [0.26; 3.22]	Ipi+Dac	.	.
0.04 [0.00; 0.37]	0.04 [0.01; 0.22]	0.05 [0.00; 0.61]	0.07 [0.01; 0.73]	0.11 [0.03; 0.45]	0.12 [0.03; 0.51]	0.11 [0.01; 1.28]	0.13 [0.02; 1.01]	0.15 [0.02; 1.04]	0.17 [0.02; 1.19]	0.17 [0.02; 1.19]	0.16 [0.03; 0.96]	0.20 [0.03; 1.29]	0.23 [0.02; 2.93]	0.27 [0.09; 0.81]	0.44 [0.22; 0.89]	0.47 [0.06; 3.46]	0.50 [0.12; 2.13]	0.52 [0.08; 3.62]	0.57 [0.10; 3.17]	Atez+Cobi	.
0.01 [0.00; 0.23]	0.01 [0.00; 0.18]	0.01 [0.00; 0.43]	0.02 [0.00; 0.45]	0.03 [0.00; 0.51]	0.03 [0.00; 0.57]	0.03 [0.00; 0.78]	0.03 [0.00; 0.86]	0.04 [0.00; 0.74]	0.04 [0.00; 0.85]	0.04 [0.00; 0.85]	0.04 [0.00; 0.74]	0.05 [0.00; 0.95]	0.06 [0.00; 1.75]	0.07 [0.00; 1.37]	0.11 [0.00; 2.54]	0.12 [0.01; 2.44]	0.12 [0.01; 2.35]	0.13 [0.01; 2.61]	0.14 [0.01; 2.53]	0.25 [0.01; 6.27]	Pima

Supplementary Table S6.1. Risk of bias table across studies.

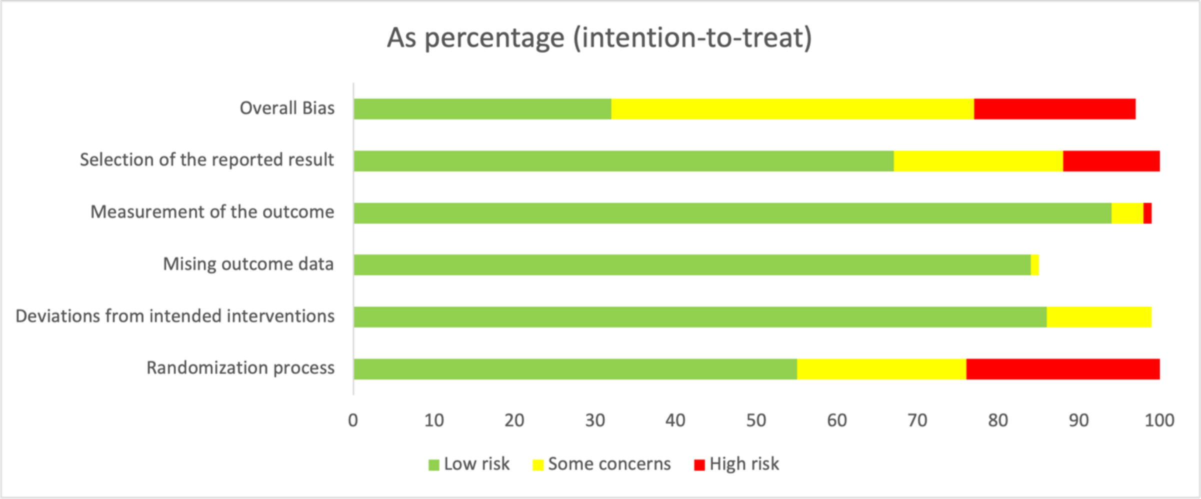

Supplementary Table S6.2. Risk of bias table for all outcomes

Intention-to-treat

- + Low risk
- ! Some concerns
- - High risk

D1 Randomisation process
D2 Deviations from the intended interventions
D3 Missing outcome data
D4 Measurement of the outcome
D5 Selection of the reported result

Study ID	Outcom	Weight	D1	D2	D3	D4	D5	Overall
KEYNOTE-022	PFS	1	+	+	+	+	+	+
KEYNOTE-022	OS	1	+	+	+	+	+	+
KEYNOTE-022	ORR	1	+	+	+	+	+	+
KEYNOTE-022	AE	1	+	+	+	+	+	+
KEYNOTE-022	DAE	1	+	+	+	+	+	+
COMBI-i	PFS	1	+	+	+	+	!	!
COMBI-i	OS	1	+	+	!	+	-	-
COMBI-i	ORR	1	+	+	+	+	+	+
COMBI-i	AE	1	+	+	+	+	+	+
COMBI-i	DAE	1	+	+	+	+	+	+
IMspire150	PFS	1	+	+	+	+	+	+
IMspire150	OS	1	+	+	+	+	-	-
IMspire150	ORR	1	+	+	+	+	+	+
IMspire150	AE	1	+	+	+	+	+	+
IMspire150	DAE	1	+	+	+	+	+	+
IMspire170	PFS	1	-	+	+	+	+	-
IMspire170	OS	1	-	+	+	+	!	-
IMspire170	ORR	1	-	+	+	+	+	-
IMspire170	AE	1	-	+	+	+	-	-
IMspire170	DAE	1	-	+	+	+	-	-
Combi-v	PFS	1	+	!	+	+	+	!
Combi-v	OS	1	+	!	+	+	+	!
Combi-v	ORR	1	+	!	+	+	+	!
Combi-v	AE	1	+	!	+	+	+	!
COMBI-d	PFS	1	+	+	+	+	+	+
COMBI-d	OS	1	+	+	+	+	+	+
COMBI-d	ORR	1	+	+	+	+	+	+
COMBI-d	AE	1	+	+	+	+	+	+
COMBI-d	DAE	1	+	+	+	+	+	+
COLUMBUS	PFS	1	+	+	+	+	!	!
COLUMBUS	OS	1	+	+	+	+	!	!
COLUMBUS	AE	1	+	+	+	+	!	!
COLOMBUS	ORR	1	+	+	+	+	!	!
COLOMBUS	DAE	1	+	+	+	+	!	!
NCT02374242	OS	1	+	+	+	+	!	!
NCT02374242	AE	1	+	+	+	+	!	!
NCT02374242	DAE	1	+	+	+	!	+	!
NCT01072175 (Part C)	PFS	1	!	+	+	+	!	-
NCT01072175 (Part C)	OS	1	!	+	+	+	!	-
NCT01072175 (Part C)	ORR	1	!	+	+	+	+	-
NCT01072175 (Part C)	AE	1	!	+	+	+	+	!
NCT01072175 (Part C)	DAE	1	!	+	+	+	+	-
CheckMate 069	PFS	1	+	+	+	+	-	-
CheckMate 069	OS	1	+	+	+	+	-	-
CheckMate 069	AE	1	+	+	+	+	-	-
CheckMate 069	ORR	1	+	+	+	+	+	+
CheckMate 069	DAE	1	+	+	+	+	+	+
coBRIM	OS	1	+	+	+	+	+	+
coBRIM	PFS	1	+	+	+	+	+	+
coBRIM	ORR	1	+	+	+	+	+	+
coBRIM	AE	1	+	+	+	+	+	+
coBRIM	DAE	1	+	+	+	+	+	+
Keynote-006	PFS	1	+	+	+	+	+	+
Keynote-006	OS	1	+	+	+	+	+	+
Keynote-006	ORR	1	+	+	+	+	+	+
Keynote-006	AE	1	+	+	+	+	+	+
Keynote-006	DAE	1	+	+	+	+	+	+
CheckMate-067	PFS	1	!	!	+	+	+	!
CheckMate-067	OS	1	!	!	+	+	+	!
CheckMate-067	ORR	1	+	+	+	+	+	+
CheckMate-067	AE	1	!	!	+	+	!	!
CheckMate-067	DAE	1	+	+	+	+	+	+
Pacmel	PFS	1	+	+	+	+	+	+
Pacmel	OS	1	+	+	+	+	+	+
Pacmel	AE	1	+	+	+	+	+	+
Pacmel	ORR	1	+	+	+	+	+	+
Break_3	PFS	1	+	!	+	+	+	!
Break_3	OS	1	+	!	+	+	+	!
Break_3	ORR	1	+	!	+	+	+	!

Study ID	Outcome	Weight	D1	D2	D3	D4	D5	Overall
Break_3	AE	1	+	!	+	+	!	-
Break_3	DAE	1	+	!	+	+	!	-
CheckMate037	PFS	1	+	!	+	+	+	!
CheckMate037	OS	1	+	!	+	+	+	!
CheckMate037	ORR	1	+	!	+	+	+	!
CheckMate037	AE	1	+	!	+	+	+	!
CheckMate037	DAE	1	+	!	+	+	+	!
CheckMate066	PFS	1	+	+	+	+	+	+
CheckMate066	OS	1	+	+	+	+	+	+
CheckMate066	ORR	1	+	+	+	+	+	+
CheckMate066	AE	1	+	+	+	+	+	+
CheckMate066	DAE	1	+	+	+	+	+	+
CA 184-024	PFS	1	+	+	+	+	+	+
CA 184-084	OS	1	+	+	+	+	+	+
CA 184-024	ORR	1	+	+	+	+	+	+
CA 184-024	AE	1	+	+	+	+	+	+
CA 184-024	DAE	1	+	+	+	+	+	+
METRIC	PFS	1	!	!	+	+	+	-
METRIC	OS	1	!	!	+	+	+	-
METRIC	ORR	1	!	!	+	+	+	-
METRIC	AE	1	!	!	+	+	+	-
METRIC	DAE	1	!	!	+	+	+	-
Brim3	PFS	1	+	!	+	+	+	+
Brim3	OS	1	+	!	+	+	+	+
Brim3	ORR	1	+	!	+	+	+	+
Brim3	AE	1	+	!	+	+	+	+
Brim3	DAE	1	+	!	+	+	+	+
NCT00936221	PFS	1	+	+	+	+	+	+
NCT00936221	OS	1	+	+	+	+	+	+
NCT00936221	ORR	1	+	+	+	+	+	+
NCT00936221	AE	1	+	+	+	+	+	+
NCT00936221	DAE	1	+	+	+	+	+	+
Keynote002	PFS	1	+	+	+	+	+	+
Keynote002	OS	1	+	+	+	+	+	+
Keynote002	ORR	1	+	+	+	+	+	+
Keynote002	AE	1	+	+	+	+	+	+
Keynote002	DAE	1	+	+	+	+	+	+
Ribas2013	PFS	1	!	!	+	+	+	-
Ribas2013	OS	1	!	!	+	+	+	-
Ribas2013	ORR	1	!	!	+	+	+	-
Ribas2013	AE	1	!	!	+	+	+	-
Ribas2013	DAE	1	!	!	+	+	+	-
NCT00050102	PFS	1	!	!	+	+	+	!
NCT00050102	OS	1	!	!	+	+	+	!
NCT00050102	ORR	1	!	!	+	+	+	!
NCT00050102	AE	1	!	!	+	+	+	!
NCT00050102	DAE	1	!	!	+	+	+	!
NCT01693068	PFS	1	!	+	+	+	!	!
NCT01693068	OS	1	!	+	+	+	!	!
NCT01693068	ORR	1	!	+	+	+	!	!
NCT01693068	AE	1	!	+	+	+	!	!
NCTO1693068	DEA	1	!	+	+	+	!	!
NCT00338130	PFS	1	-	!	+	+	+	-
NCT00338130	ORR	1	-	!	+	-	+	-
NCT00338130	AE	1	-	!	+	!	+	-
NCT00338130	DAE	1	-	!	+	!	+	-
NEMO	PFS	1	-	!	+	+	+	-
NEMO	OS	1	-	!	+	+	+	-
NEMO	ORR	1	-	!	+	+	+	-
NEMO	AE	1	-	!	+	+	+	-
NEMO	DAE	1	-	!	+	+	+	-
NIBIT - M2	PFS	1	-	+	+	+	+	-
NIBIT - M2	OS	1	-	+	+	+	+	-
NIBIT - M2	ORR	1	-	+	+	+	+	-
NIBIT - M2	AE	1	-	+	+	+	+	-
NIBIT - M2	DAE	1	-	+	+	+	+	-
DOC-MEK	PFS	1	+	+	+	!	+	!
DOC-MEK	OS	1	+	+	+	+	+	+
DOC-MEK	ORR	1	+	+	+	+	+	+

Supplementary Figure S4a. Evidence plot for pooled PFS (HR)

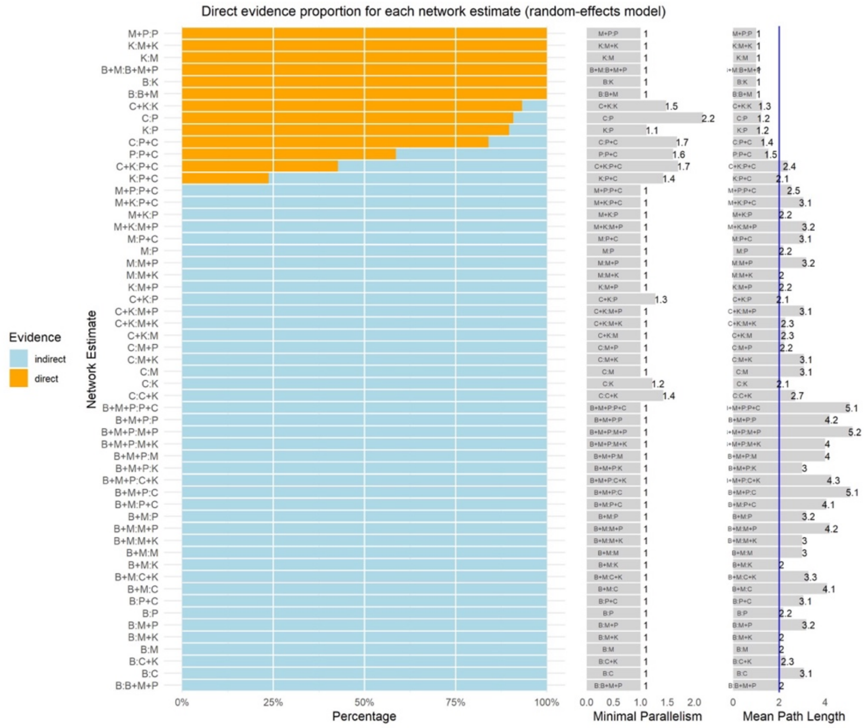

Supplementary Figure S4b. Evidence plot for BRAF subgroup (PFS-HR).

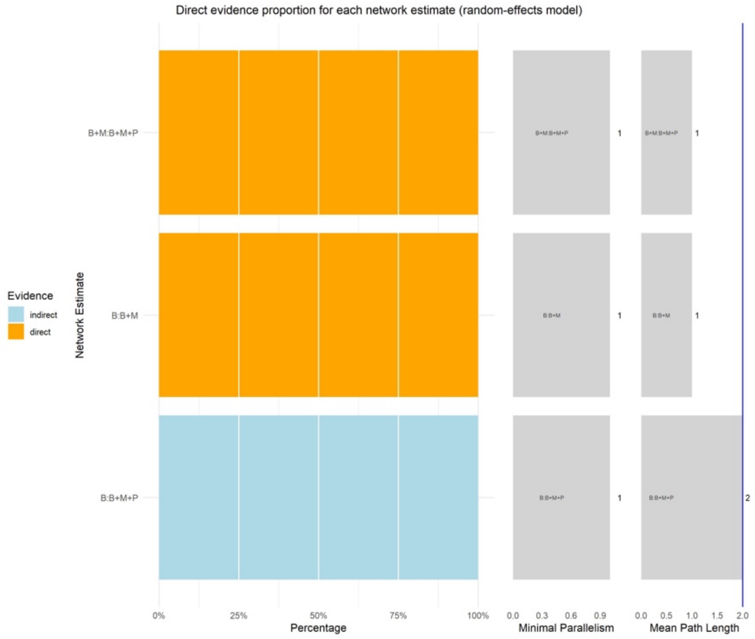

Supplementary Figure S5a. Evidence plot for pooled ORR (OR)

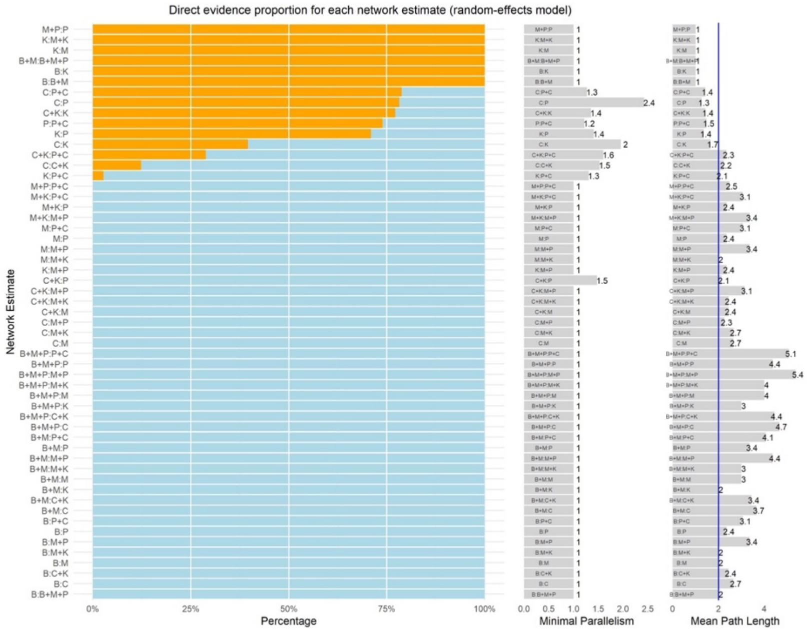

Supplementary Figure S5b. Evidence plot for BRAF subgroup (ORR-OR).

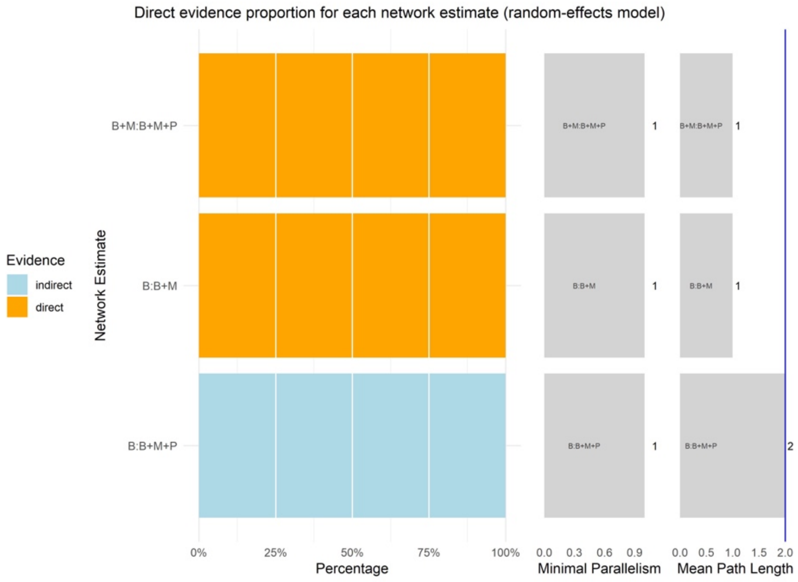

Supplementary Figure S6a. Evidence plot for pooled CRR (OR)

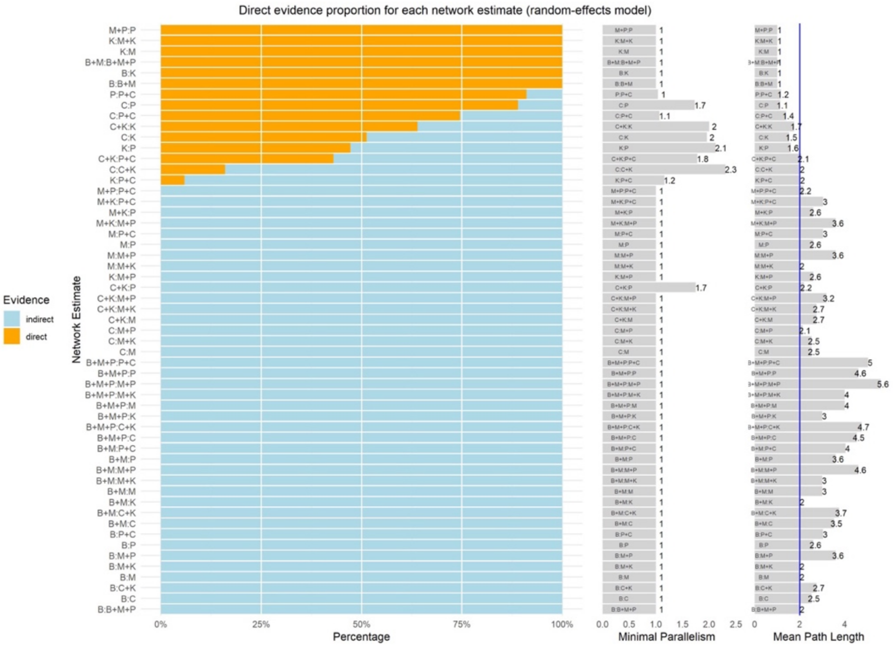

Supplementary Figure S6b. Evidence plot for BRAF subgroup (CRR-OR).

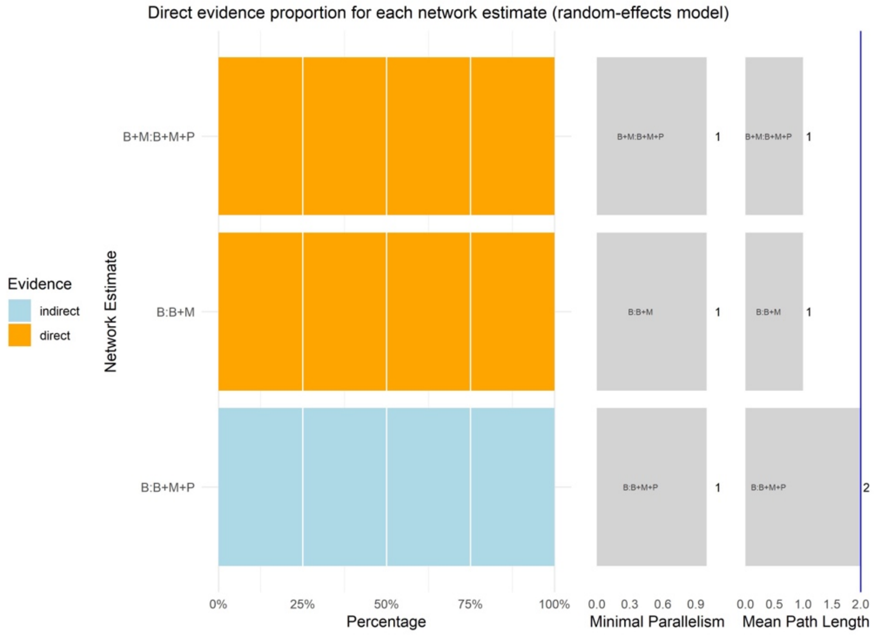

Supplementary Figure S7. Evidence plot for pooled AEs (OR)

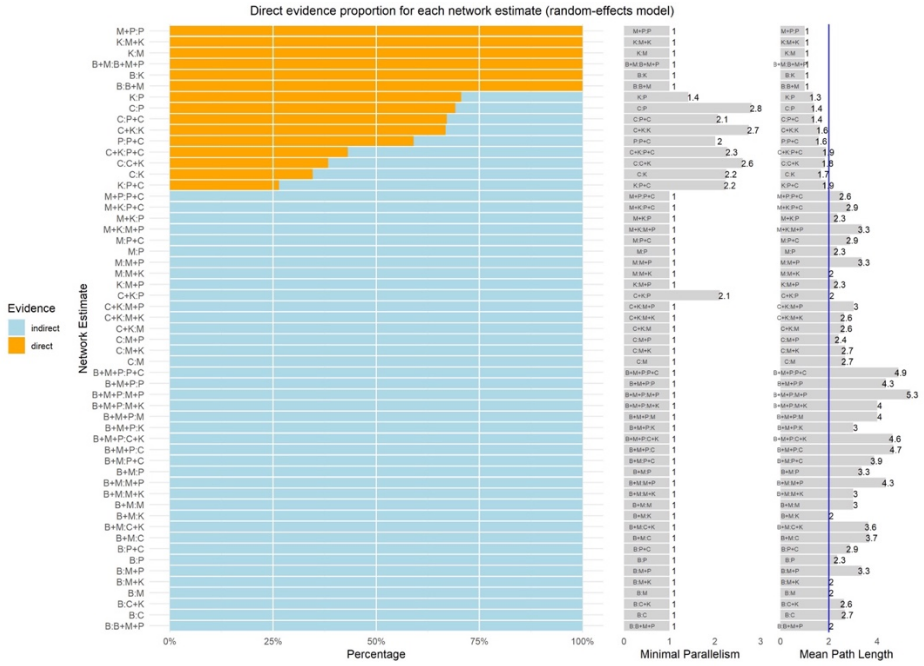

Supplementary Figure S8. Evidence plot for pooled SAEs (OR)

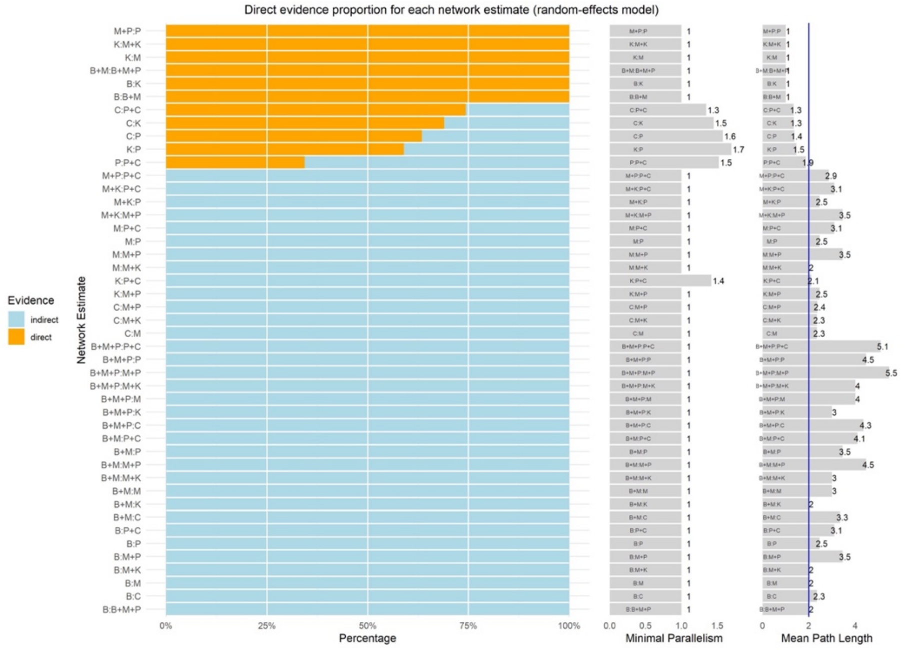

Supplementary Figure S9. Evidence plot for pooled grade 3≤ AEs (OR)

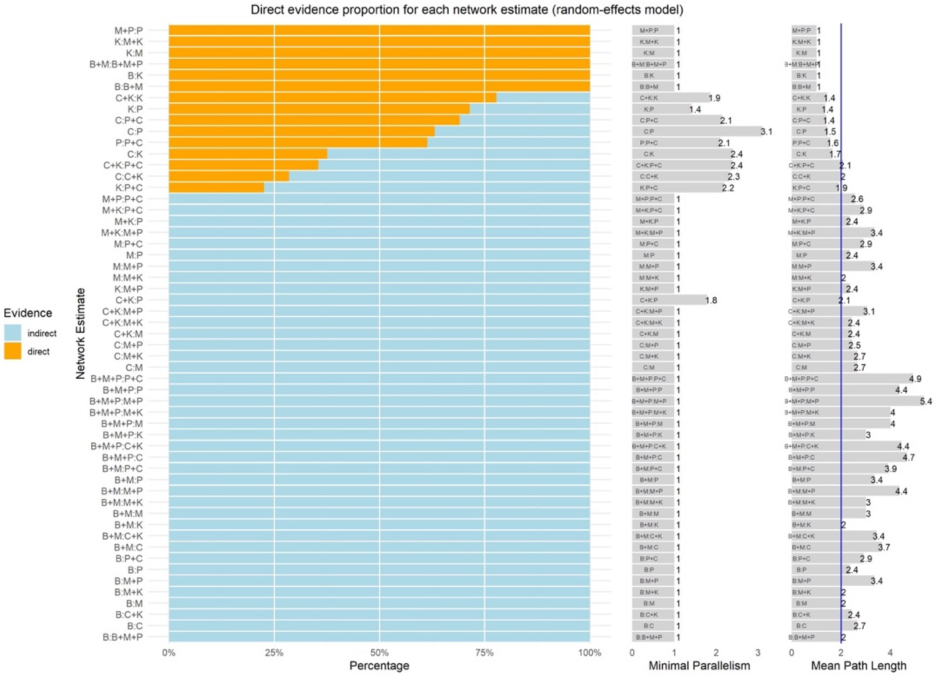

Supplementary Figure S10. Evidence plot for pooled TDR (OR)

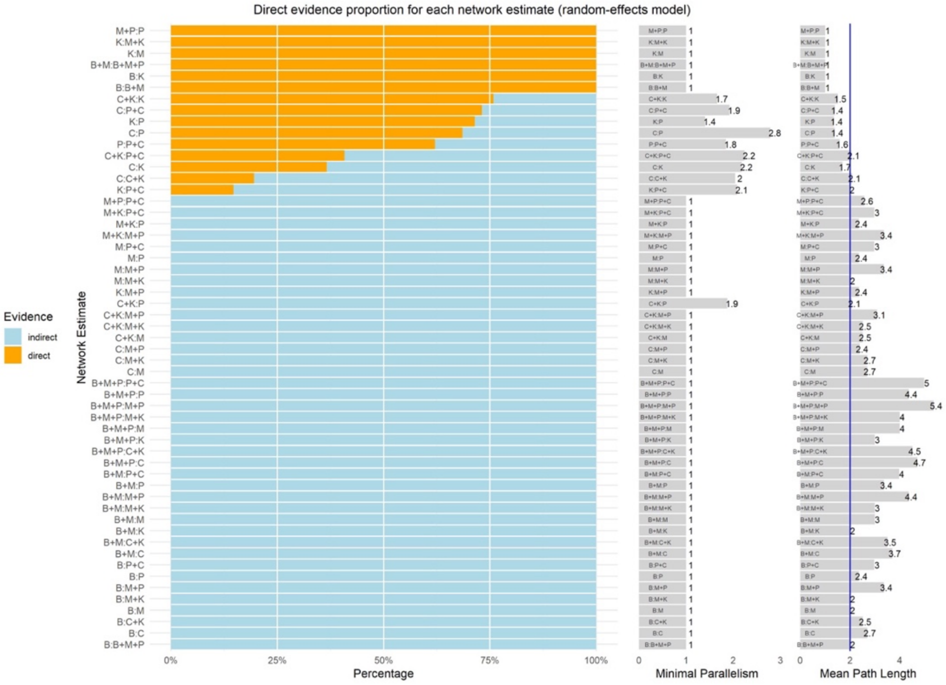

Supplementary Figure S11. Evidence plot for PFS (HR)

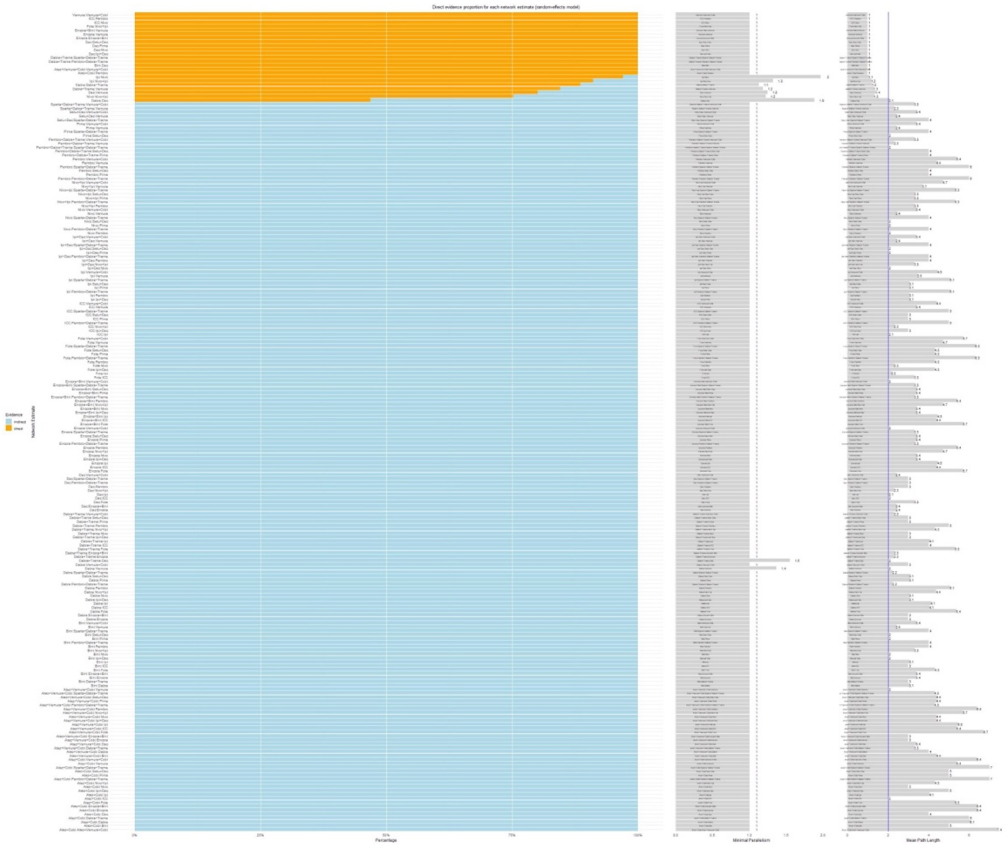

Supplementary Figure S12. Evidence plot for ORR (OR)

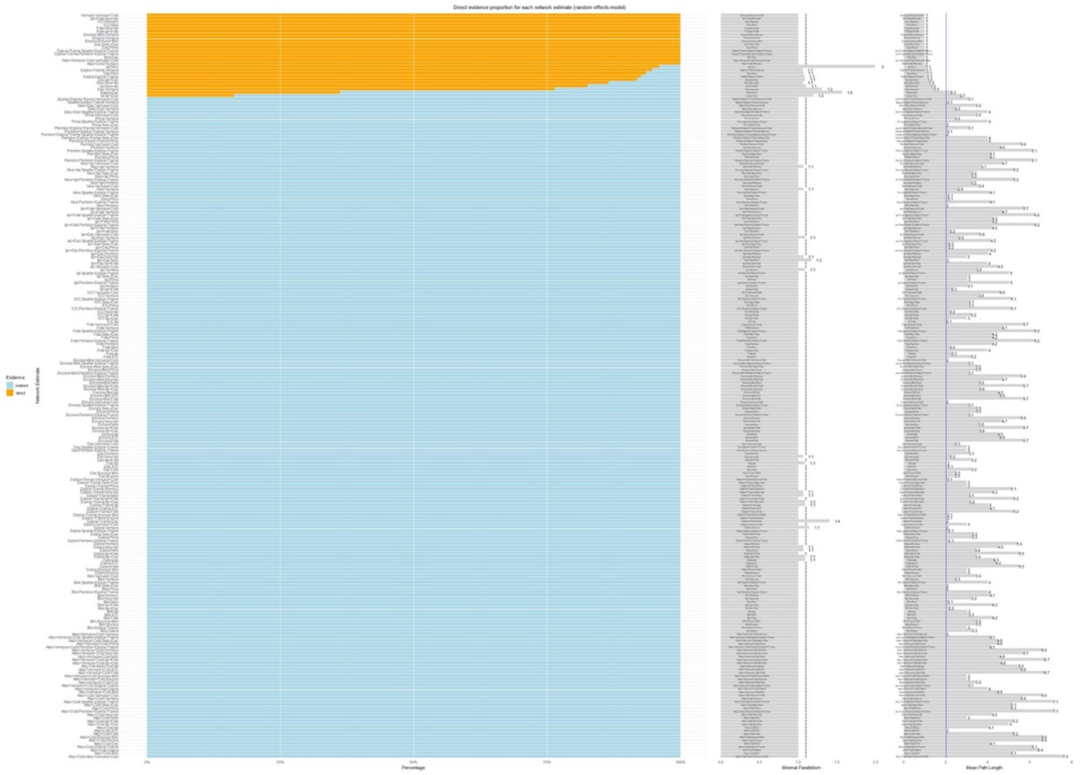

Supplementary Figure S13. Evidence plot for CRR (OR)

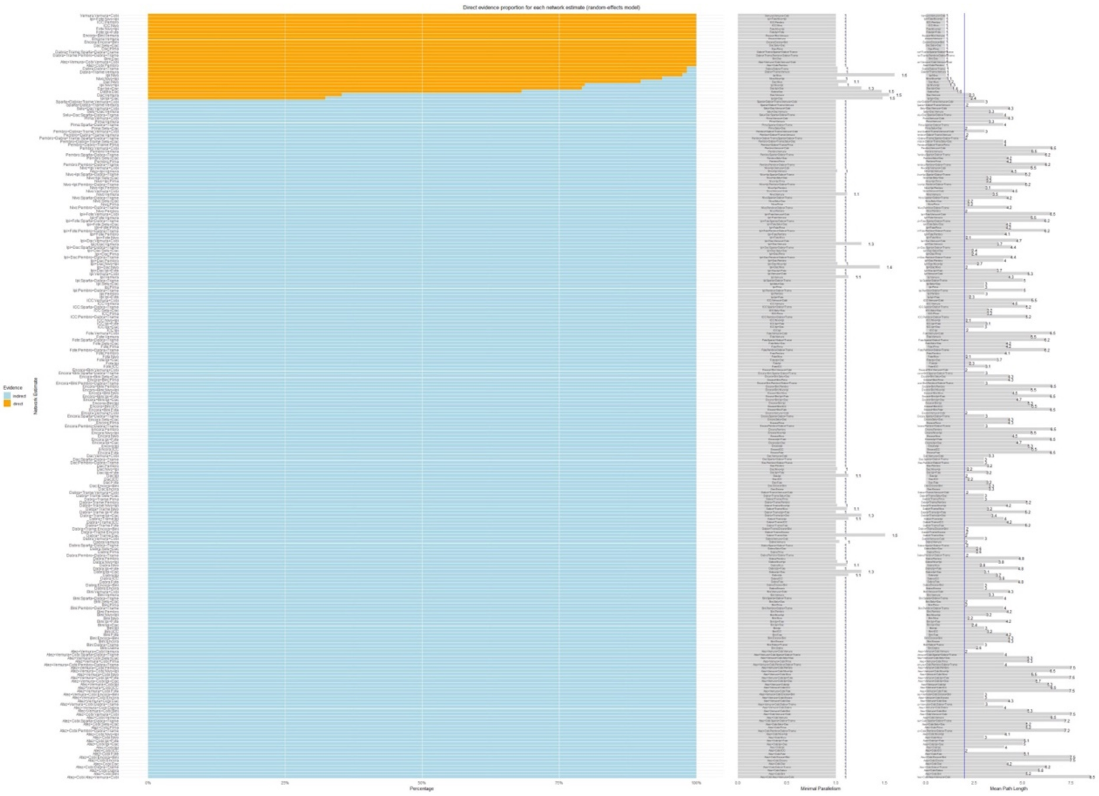

Supplementary Figure S14. Evidence plot for AEs (OR)

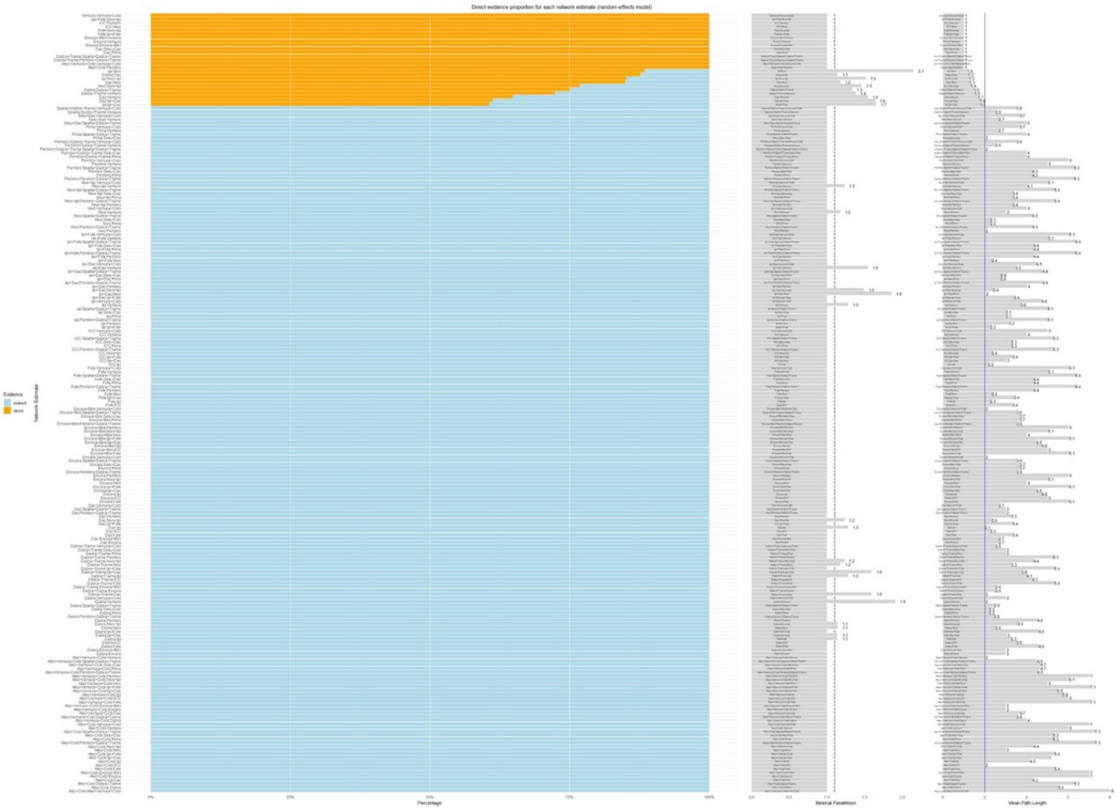

Supplementary Figure S15. Evidence plot for grade 3≤ AEs (OR)

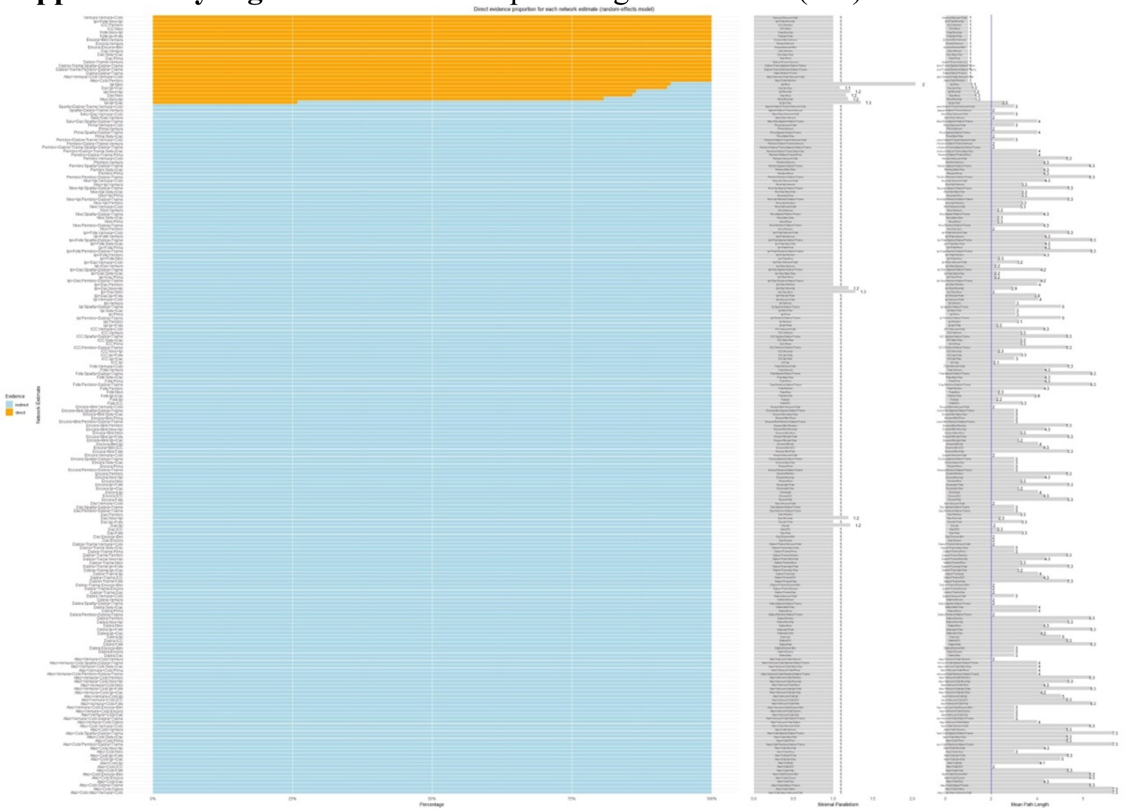

Supplementary Figure S16. Evidence plot for TDR (OR)

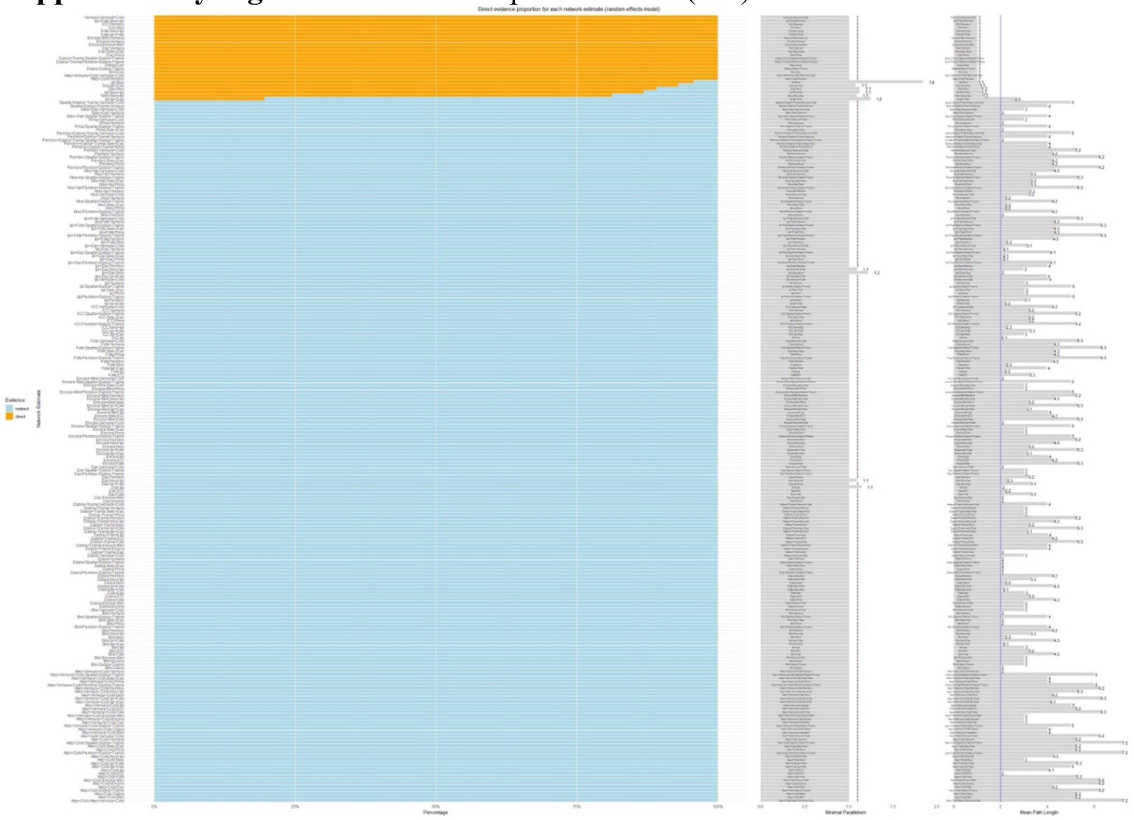

Supplementary Figure S17a. Funnel plot for pooled PFS (HR).

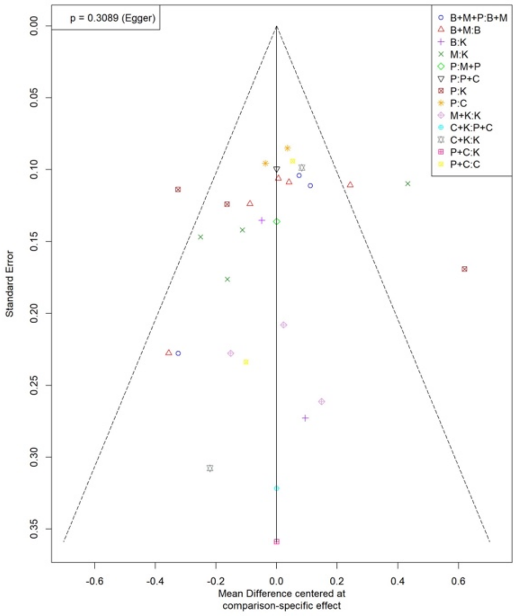

Supplementary Figure S17b. Funnel plot for BRAF subgroup (PFS-HR).

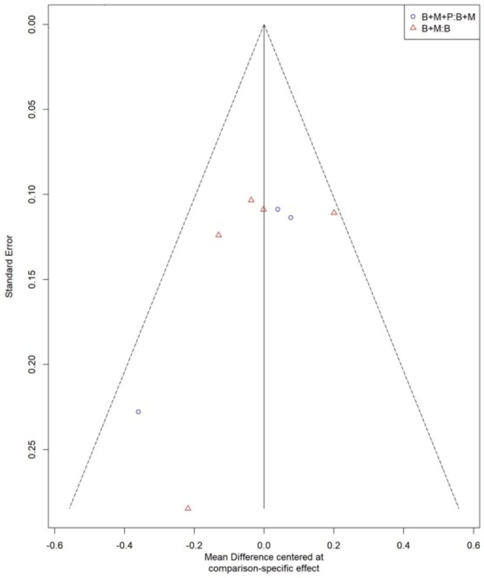

Supplementary Figure S18a. Funnel plot for pooled ORR (OR).

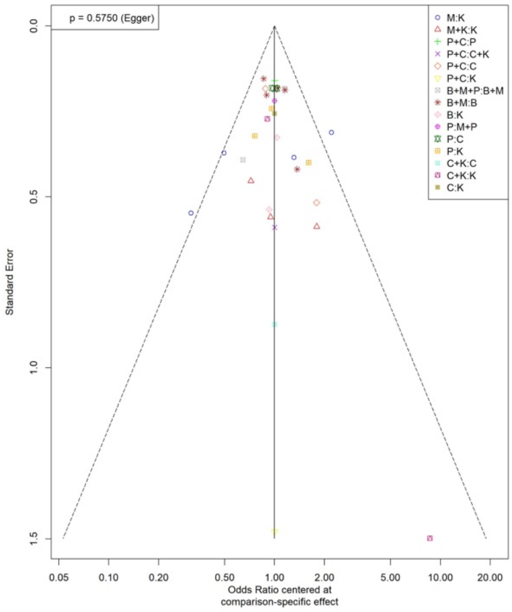

Supplementary Figure S18b. Funnel plot for BRAF subgroup (ORR-OR).

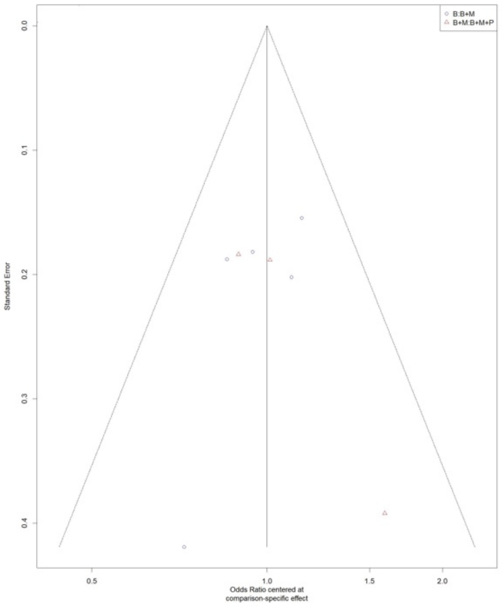

Supplementary Figure S19a. Funnel plot for pooled CRR (OR).

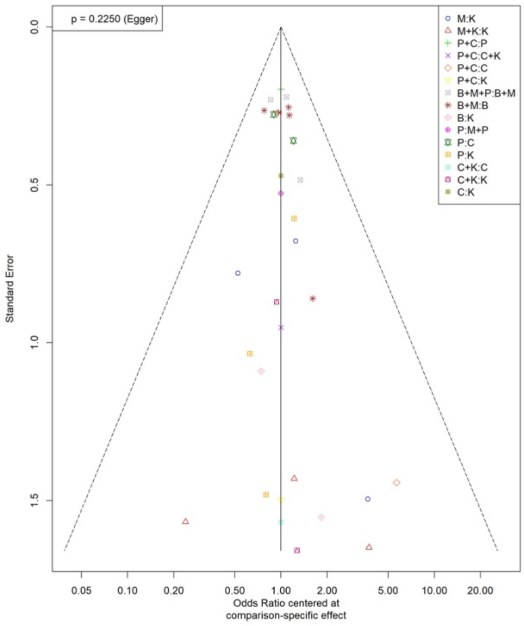

Supplementary Figure S19b. Funnel plot for BRAF subgroup (CRR-OR).

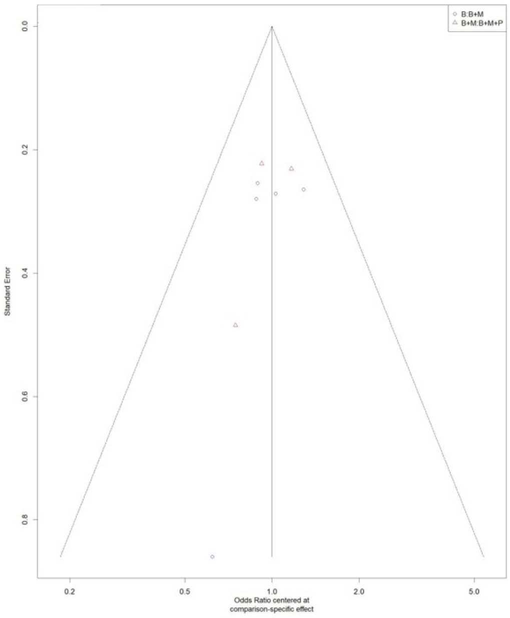

Supplementary Figure S20. Funnel plot for pooled TRAE (OR).

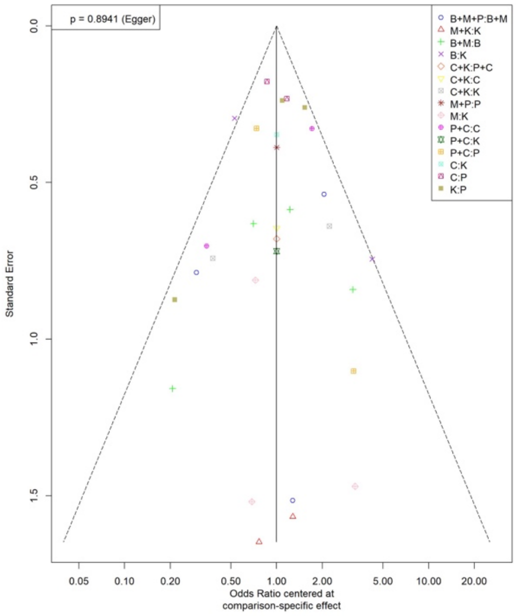

Supplementary Figure S21. Funnel plot for pooled SAE (OR)

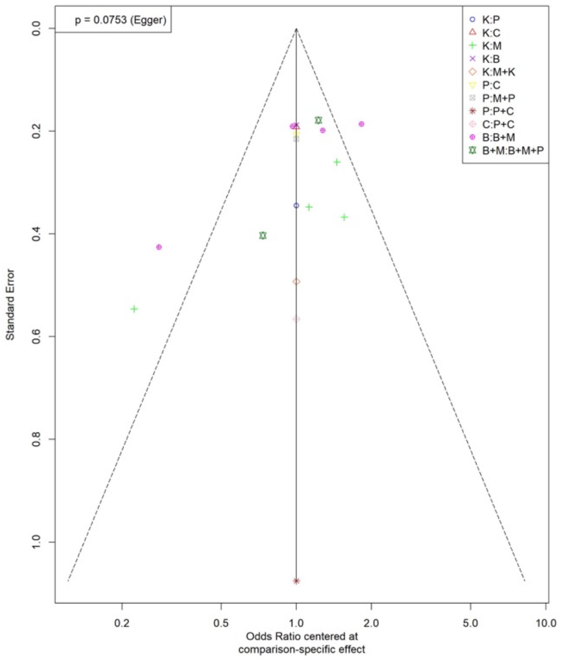

Supplementary Figure S22. Funnel plot for pooled grade 3≤ AE (OR)

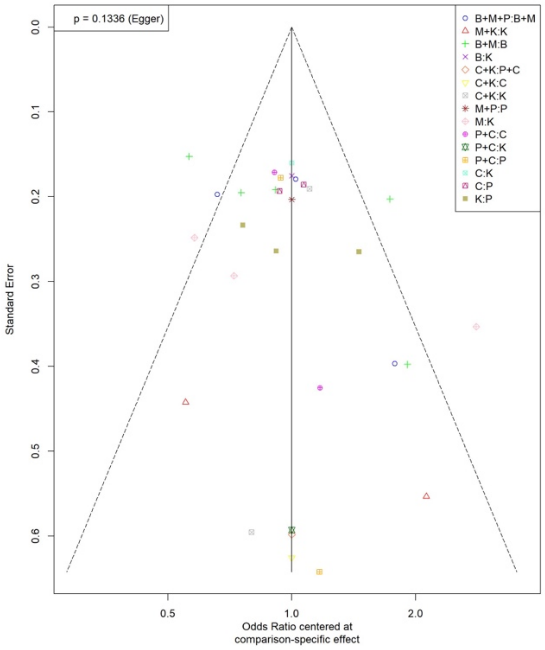

Supplementary Figure S23. Funnel plot for pooled TDR (OR)

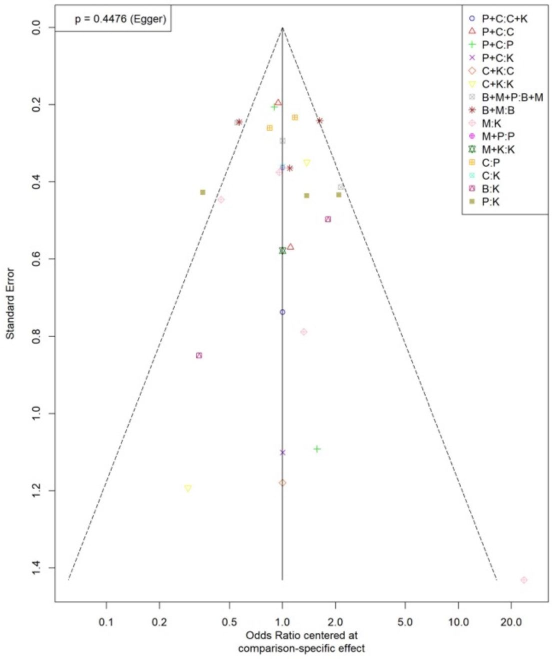

Supplementary Figure S24. Funnel plot for PFS (HR)

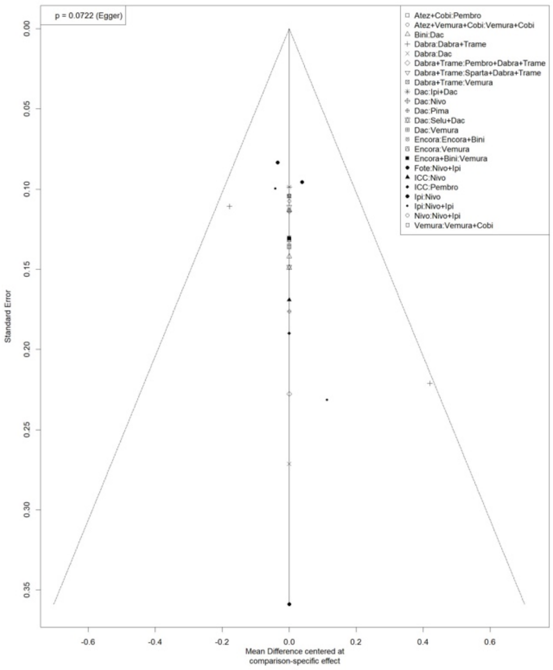

Supplementary Figure S25. Funnel plot for ORR (OR)

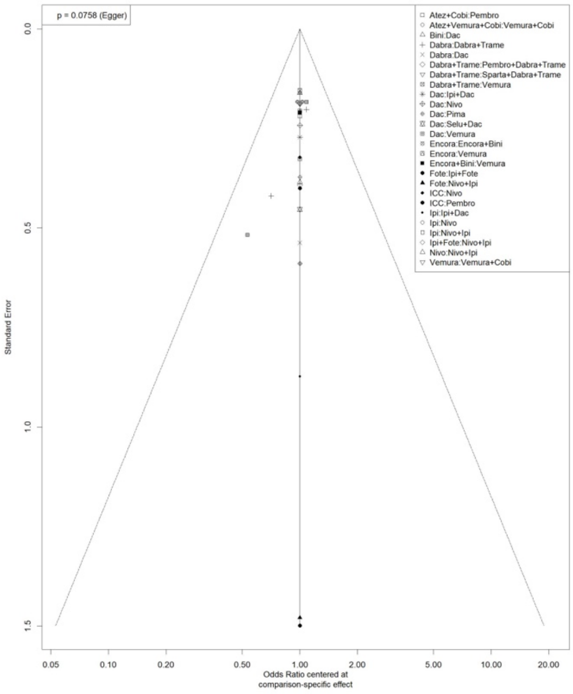

Supplementary Figure S26. Funnel plot for CRR (OR)

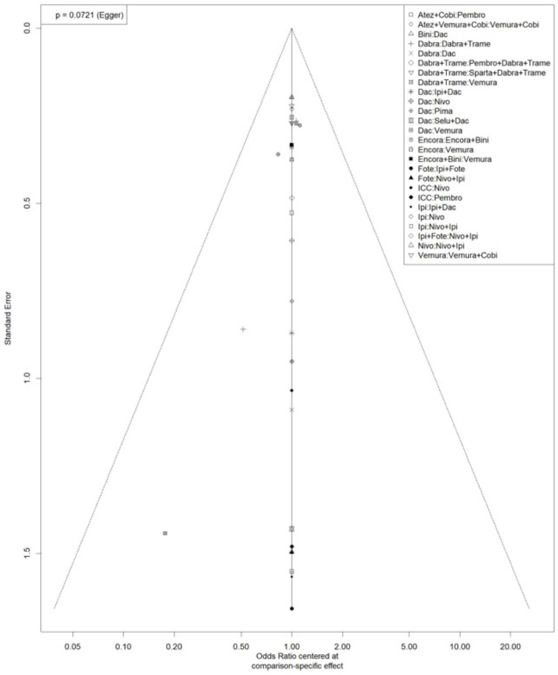

Supplementary Figure S27. Funnel plot for TRAE (OR)

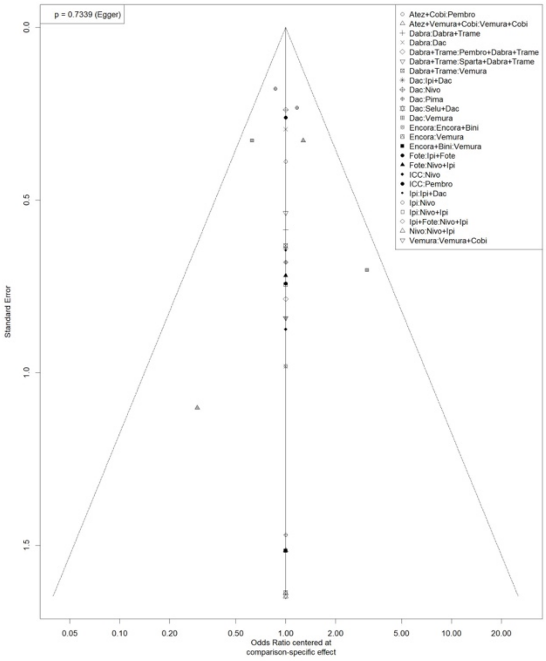

Supplementary Figure S28. Funnel plot for grade 3≤ AEs (OR)

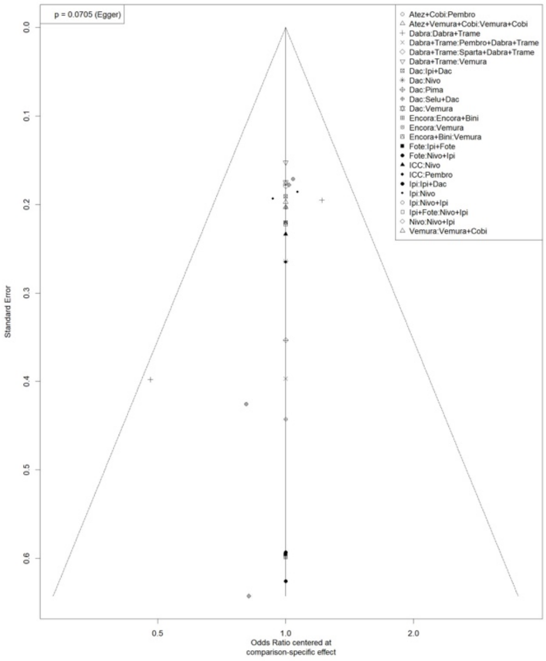

Supplementary Figure S29. Funnel plot for TDR (OR)

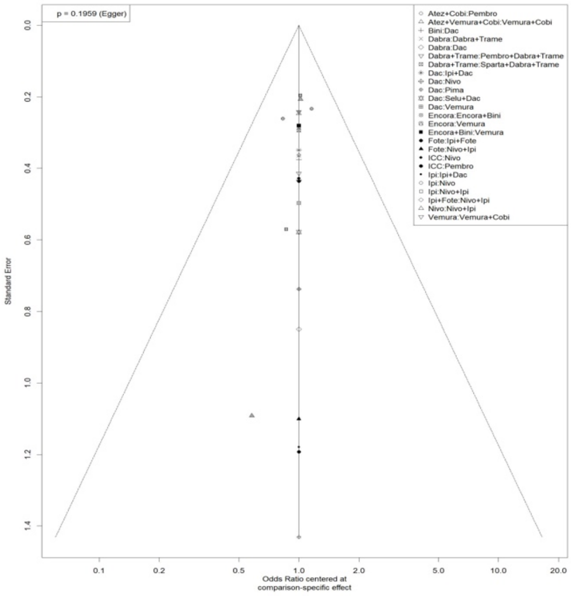

Supplementary Figure S30. Forest plot for pooled PFS (HR)

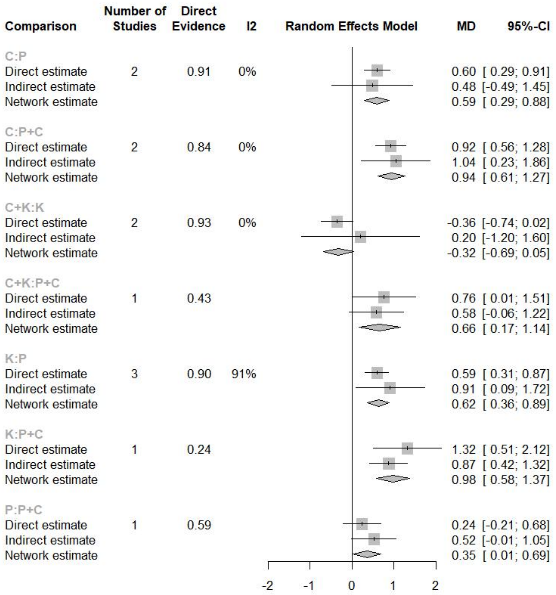

Supplementary Figure S31. Forest plot for pooled ORR (OR)

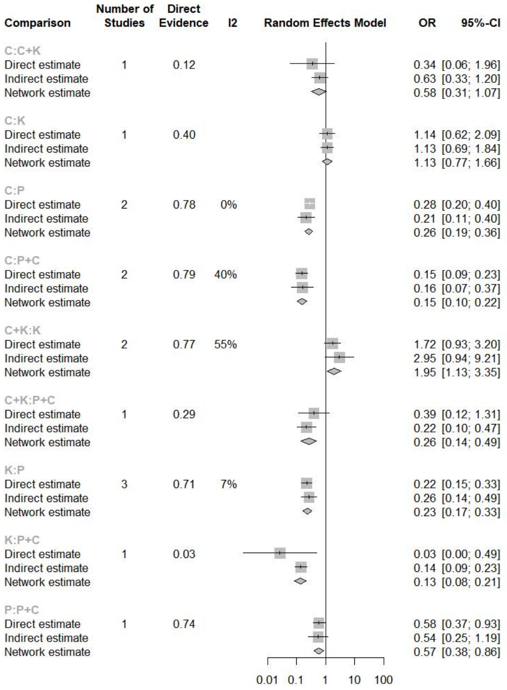

Supplementary Figure S32. Forest plot for pooled CRR (OR)

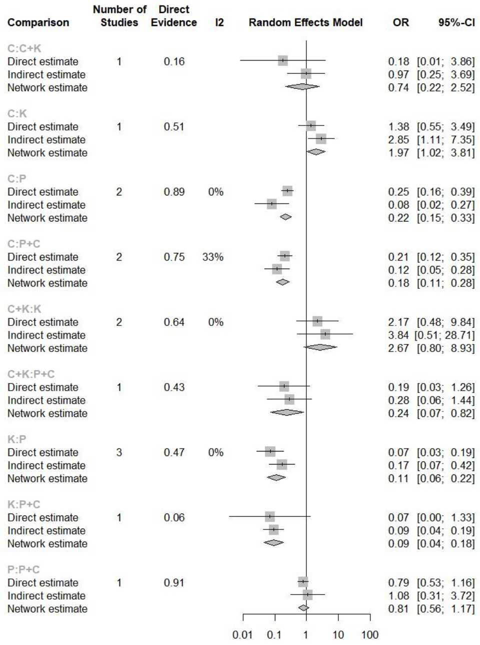

Supplementary Figure S33. Forest plot for pooled TRAE (OR)

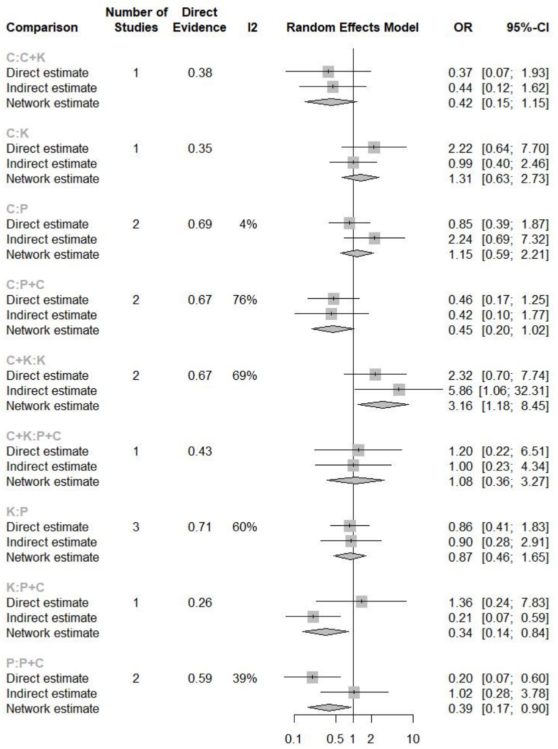

Supplementary Figure S34. Forest plot for pooled SAE (OR)

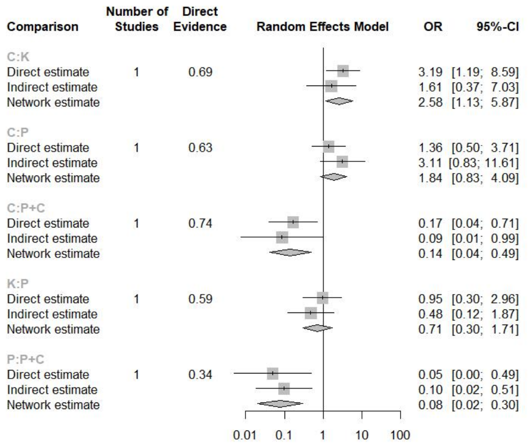

Supplementary Figure S35. Forest plot for pooled grade 3≤ AE (OR)

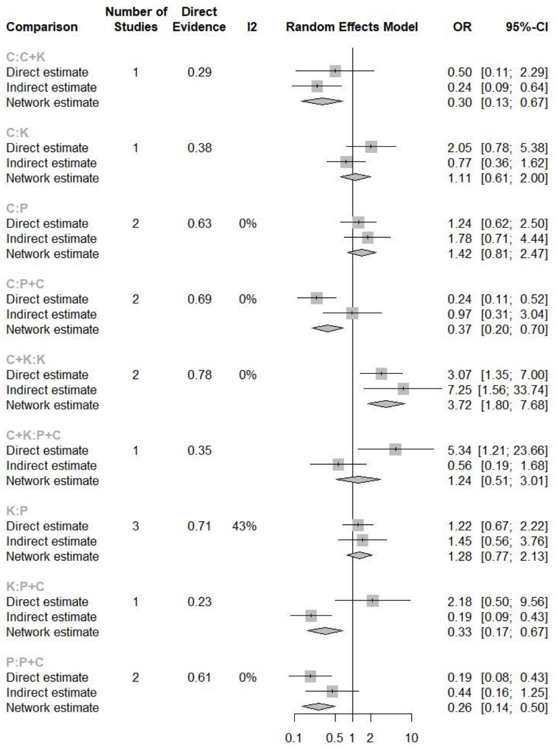

Supplementary Figure S36. Forest plot for pooled TDR (OR)

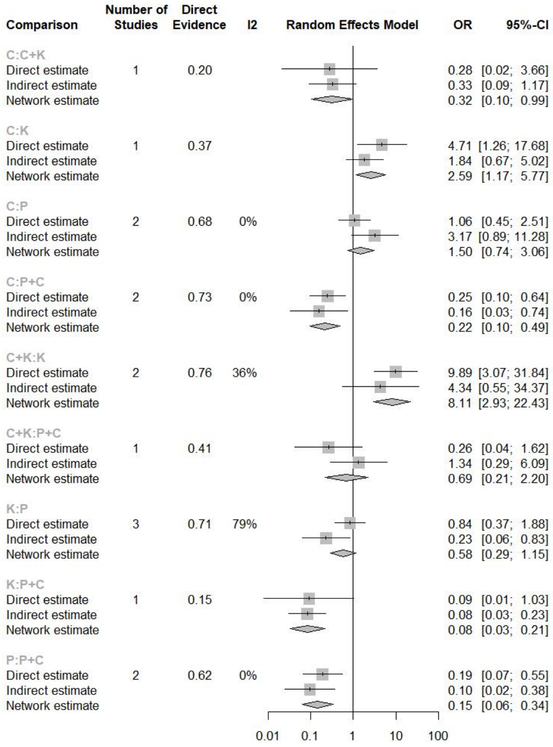

Supplementary Figure S37. Forest plot for PFS (HR)

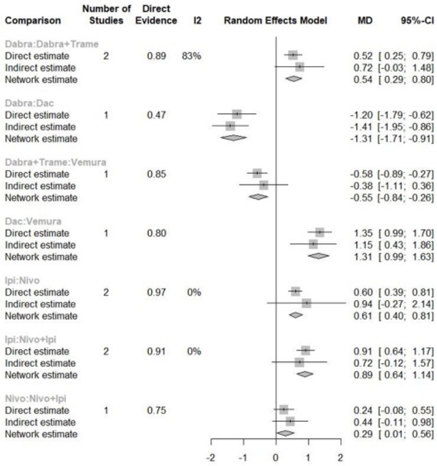

Supplementary Figure S38. Forest plot for ORR (OR)

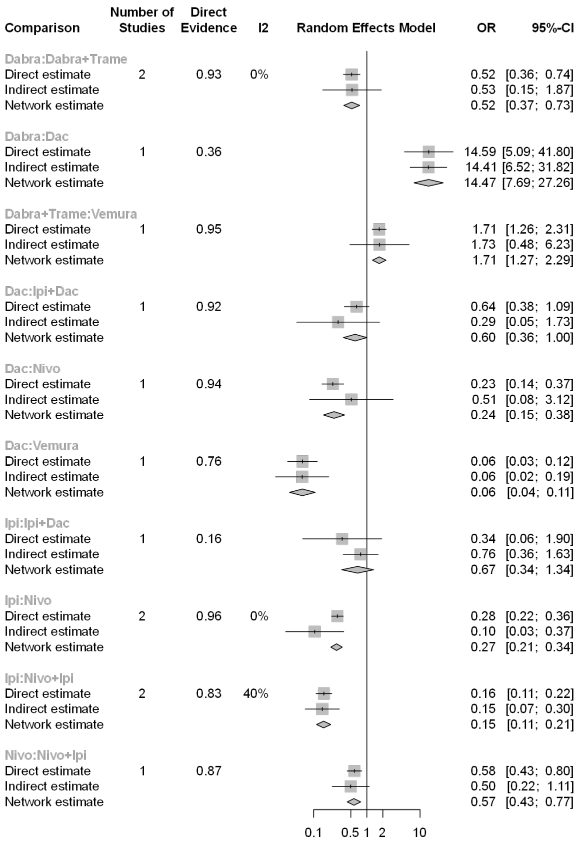

Supplementary Figure S39. Forest plot for CRR (OR)

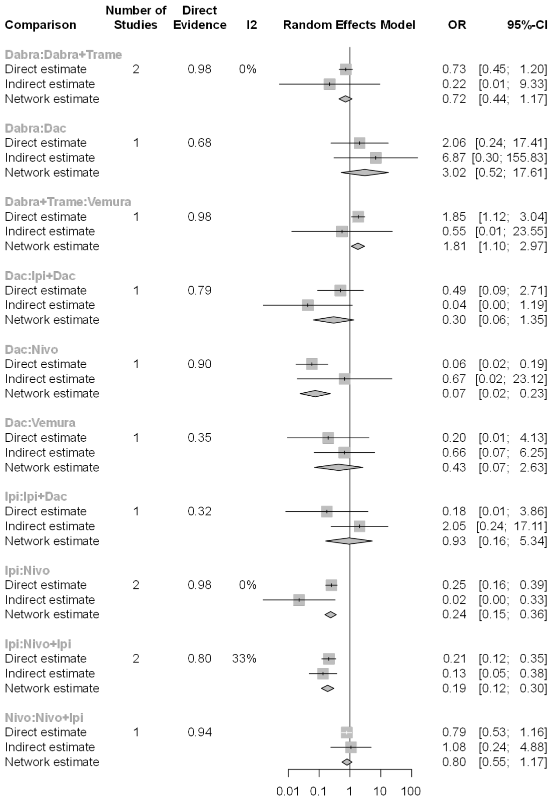

Supplementary Figure S40. Forest plot for TRAE (OR)

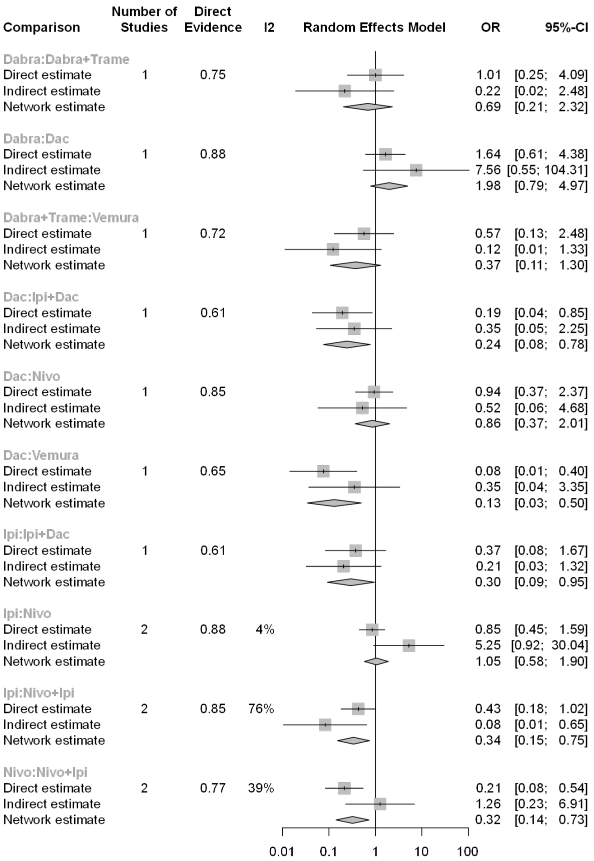

Supplementary Figure S41. Forest plot for grade 3≤ AEs (OR)

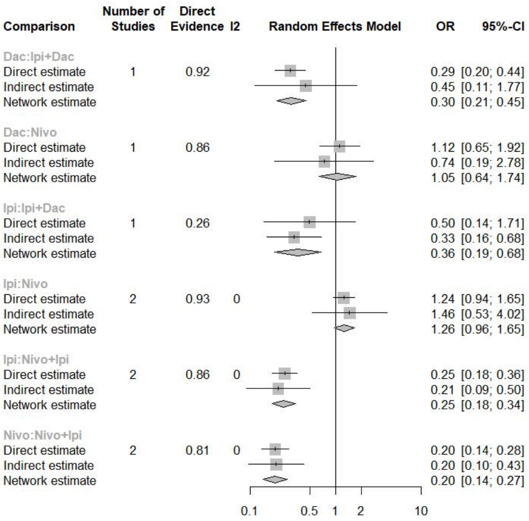

Supplementary Figure S42. Forest plot for TDR (OR)

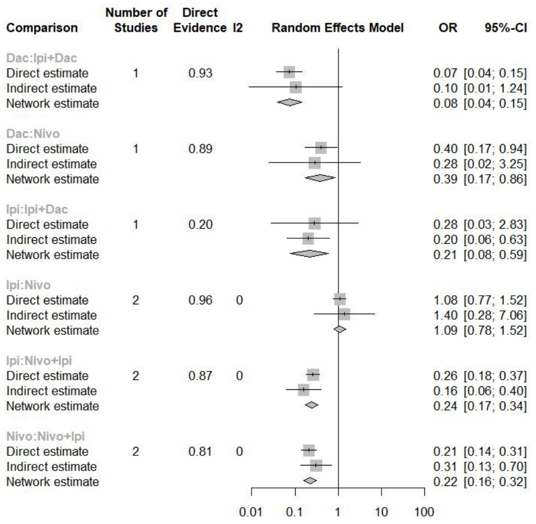

Supplementary Table S7. CINEMA analysis for ORR (OR)

Comparison	Number of studies	Within-study bias	Reporting bias	Indirectness	Imprecision	Heterogeneity	Incoherence	Confidence rating	Reason(s) for downgrading
B:B+M	5	Some concerns	Low risk	No concerns	No concerns	No concerns	No concerns	High	[]
B:K	2	No concerns	Low risk	No concerns	Major concerns	No concerns	No concerns	Low	["Imprecision"]
B+M:B+M+P	3	No concerns	Low risk	No concerns	Major concerns	No concerns	No concerns	Low	["Imprecision"]
C:C+K	1	Some concerns	Low risk	No concerns	Major concerns	No concerns	No concerns	Low	["Within-study bias","Imprecision"]
C:K	1	Some concerns	Low risk	No concerns	No concerns	Some concerns	No concerns	Moderate	["Within-study bias","Heterogeneity"]
C:P	2	Some concerns	Low risk	No concerns	No concerns	No concerns	No concerns	High	[]
C:P+C	2	No concerns	Low risk	No concerns	No concerns	No concerns	No concerns	High	[]
C+K:K	2	Some concerns	Low risk	No concerns	Major concerns	No concerns	No concerns	Low	["Within-study bias","Imprecision"]
C+K:P+C	1	Some concerns	Low risk	No concerns	No concerns	No concerns	No concerns	High	[]
K:M	4	Some concerns	Low risk	No concerns	Major concerns	No concerns	No concerns	Low	["Within-study bias","Imprecision"]
K:M+K	3	No concerns	Low risk	No concerns	Major concerns	No concerns	No concerns	Low	["Imprecision"]
K:P	3	Some concerns	Low risk	No concerns	No concerns	No concerns	No concerns	High	[]
K:P+C	1	No concerns	Low risk	No concerns	No concerns	No concerns	No concerns	High	[]
M+P:P	1	Some concerns	Low risk	No concerns	Major concerns	No concerns	No concerns	Low	["Within-study bias","Imprecision"]
P:P+C	1	No concerns	Low risk	No concerns	Some concerns	No concerns	No concerns	Moderate	["Imprecision"]
B:B+M+P	0	No concerns	Low risk	No concerns	No concerns	No concerns	No concerns	High	[]
B:C	0	No concerns	Low risk	No concerns	Major concerns	No concerns	No concerns	Low	["Imprecision"]
B:C+K	0	No concerns	Low risk	No concerns	Major concerns	No concerns	No concerns	Low	["Imprecision"]
B:M	0	No concerns	Low risk	No concerns	Major concerns	No concerns	No concerns	Low	["Imprecision"]
B:M+K	0	No concerns	Low risk	No concerns	Major concerns	No concerns	No concerns	Low	["Imprecision"]
B:M+P	0	Some concerns	Low risk	No concerns	Major concerns	No concerns	No concerns	Low	["Within-study bias","Imprecision"]
B:P	0	No concerns	Low risk	No concerns	Major concerns	No concerns	No concerns	Low	["Imprecision"]
B:P+C	0	No concerns	Low risk	No concerns	Major concerns	No concerns	No concerns	Low	["Imprecision"]
B+M:C	0	Some concerns	Low risk	No concerns	Major concerns	No concerns	No concerns	Low	["Within-study bias","Imprecision"]
B+M:C+K	0	Some concerns	Low risk	No concerns	Major concerns	No concerns	No concerns	Low	["Within-study bias","Imprecision"]
B+M:K	0	No concerns	Low risk	No concerns	Major concerns	No concerns	No concerns	Low	["Imprecision"]
B+M:M	0	No concerns	Low risk	No concerns	Major concerns	No concerns	No concerns	Low	["Imprecision"]
B+M:M+K	0	No concerns	Low risk	No concerns	Major concerns	No concerns	No concerns	Low	["Imprecision"]
B+M:M+P	0	Some concerns	Low risk	No concerns	Major concerns	No concerns	No concerns	Low	["Within-study bias","Imprecision"]
B+M:P	0	No concerns	Low risk	No concerns	Major concerns	No concerns	No concerns	Low	["Imprecision"]
B+M:P+C	0	No concerns	Low risk	No concerns	Major concerns	No concerns	No concerns	Low	["Imprecision"]
B+M+P:C	0	No concerns	Low risk	No concerns	Major concerns	No concerns	No concerns	Low	["Imprecision"]
B+M+P:C+K	0	No concerns	Low risk	No concerns	Major concerns	No concerns	No concerns	Low	["Imprecision"]
B+M+P:K	0	No concerns	Low risk	No concerns	Major concerns	No concerns	No concerns	Low	["Imprecision"]
B+M+P:M	0	No concerns	Low risk	No concerns	Major concerns	No concerns	No concerns	Low	["Imprecision"]
B+M+P:M+K	0	No concerns	Low risk	No concerns	Major concerns	No concerns	No concerns	Low	["Imprecision"]
B+M+P:M+P	0	No concerns	Low risk	No concerns	Major concerns	No concerns	No concerns	Low	["Imprecision"]
B+M+P:P	0	No concerns	Low risk	No concerns	Major concerns	No concerns	No concerns	Low	["Imprecision"]
B+M+P:P+C	0	No concerns	Low risk	No concerns	Major concerns	No concerns	No concerns	Low	["Imprecision"]
C:M	0	Some concerns	Low risk	No concerns	Major concerns	No concerns	No concerns	Low	["Within-study bias","Imprecision"]
C:M+K	0	Some concerns	Low risk	No concerns	Major concerns	No concerns	No concerns	High	["Within-study bias","Imprecision"]
C:M+P	0	Some concerns	Low risk	No concerns	No concerns	No concerns	No concerns	High	[]
C+K:M	0	Some concerns	Low risk	No concerns	Major concerns	No concerns	No concerns	Low	["Within-study bias","Imprecision"]
C+K:M+K	0	Some concerns	Low risk	No concerns	Major concerns	No concerns	No concerns	Low	["Within-study bias","Imprecision"]
C+K:M+P	0	Some concerns	Low risk	No concerns	Major concerns	No concerns	No concerns	Low	["Within-study bias","Imprecision"]
C+K:P	0	Some concerns	Low risk	No concerns	Some concerns	No concerns	No concerns	Moderate	["Within-study bias","Imprecision"]
K:M+P	0	Some concerns	Low risk	No concerns	No concerns	No concerns	No concerns	High	[]
M:M+K	0	No concerns	Low risk	No concerns	Major concerns	No concerns	No concerns	Low	["Imprecision"]
M:M+P	0	Some concerns	Low risk	No concerns	No concerns	No concerns	No concerns	High	[]
M:P	0	Some concerns	Low risk	No concerns	No concerns	No concerns	No concerns	High	[]
M:P+C	0	No concerns	Low risk	No concerns	No concerns	No concerns	No concerns	High	[]
M+K:M+P	0	Some concerns	Low risk	No concerns	No concerns	Some concerns	No concerns	Moderate	["Within-study bias","Heterogeneity"]
M+K:P	0	No concerns	Low risk	No concerns	No concerns	No concerns	No concerns	High	[]
M+K:P+C	0	No concerns	Low risk	No concerns	No concerns	No concerns	No concerns	High	[]
M+P:P+C	0	Some concerns	Low risk	No concerns	Major concerns	No concerns	No concerns	Low	["Within-study bias","Imprecision"]

Supplementary Table S8. CINEMA analysis for CRR (OR)

Comparison	Number of studies	Within-study bias	Reporting bias	Indirectness	Imprecision	Heterogeneity	Incoherence	Confidence rating	Reason(s) for downgrading
B:B+M	5	Some concerns	Low risk	No concerns	No concerns	No concerns	No concerns	High	[]
B:K	2	No concerns	Low risk	No concerns	Major concerns	No concerns	No concerns	Low	["Imprecision"]
B+M:B+M+P	3	No concerns	Low risk	No concerns	Major concerns	No concerns	No concerns	Low	["Imprecision"]
C:C+K	1	Some concerns	Low risk	No concerns	Major concerns	No concerns	No concerns	Low	["Within-study bias","Imprecision"]
C:K	1	Some concerns	Low risk	No concerns	No concerns	Some concerns	No concerns	Moderate	["Within-study bias","Heterogeneity"]
C:P	2	Some concerns	Low risk	No concerns	No concerns	No concerns	No concerns	High	[]
C:P+C	2	No concerns	Low risk	No concerns	No concerns	No concerns	No concerns	High	[]
C+K:K	2	Some concerns	Low risk	No concerns	Major concerns	No concerns	No concerns	Low	["Within-study bias","Imprecision"]
C+K:P+C	1	Some concerns	Low risk	No concerns	No concerns	No concerns	No concerns	High	[]
K:M	4	Some concerns	Low risk	No concerns	Major concerns	No concerns	No concerns	Low	["Within-study bias","Imprecision"]
K:M+K	3	No concerns	Low risk	No concerns	Major concerns	No concerns	No concerns	Low	["Imprecision"]
K:P	3	Some concerns	Low risk	No concerns	No concerns	No concerns	No concerns	High	[]
K:P+C	1	No concerns	Low risk	No concerns	No concerns	No concerns	No concerns	High	[]
M+P:P	1	Some concerns	Low risk	No concerns	Major concerns	No concerns	No concerns	Low	["Within-study bias","Imprecision"]
P:P+C	1	No concerns	Low risk	No concerns	Some concerns	No concerns	No concerns	Moderate	["Imprecision"]
B:B+M+P	0	No concerns	Low risk	No concerns	No concerns	No concerns	No concerns	High	[]
B:C	0	No concerns	Low risk	No concerns	Major concerns	No concerns	No concerns	Low	["Imprecision"]
B:C+K	0	No concerns	Low risk	No concerns	Major concerns	No concerns	No concerns	Low	["Imprecision"]
B:M	0	No concerns	Low risk	No concerns	Major concerns	No concerns	No concerns	Low	["Imprecision"]
B:M+K	0	No concerns	Low risk	No concerns	Major concerns	No concerns	No concerns	Low	["Imprecision"]
B:M+P	0	Some concerns	Low risk	No concerns	Major concerns	No concerns	No concerns	Low	["Within-study bias","Imprecision"]
B:P	0	No concerns	Low risk	No concerns	Major concerns	No concerns	No concerns	Low	["Imprecision"]
B:P+C	0	No concerns	Low risk	No concerns	Major concerns	No concerns	No concerns	Low	["Imprecision"]
B+M:C	0	Some concerns	Low risk	No concerns	Major concerns	No concerns	No concerns	Low	["Within-study bias","Imprecision"]
B+M:C+K	0	Some concerns	Low risk	No concerns	Major concerns	No concerns	No concerns	Low	["Within-study bias","Imprecision"]
B+M:K	0	No concerns	Low risk	No concerns	Major concerns	No concerns	No concerns	Low	["Imprecision"]
B+M:M	0	No concerns	Low risk	No concerns	Major concerns	No concerns	No concerns	Low	["Imprecision"]
B+M:M+K	0	No concerns	Low risk	No concerns	Major concerns	No concerns	No concerns	Low	["Imprecision"]
B+M:M+P	0	Some concerns	Low risk	No concerns	Major concerns	No concerns	No concerns	Low	["Within-study bias","Imprecision"]
B+M:P	0	No concerns	Low risk	No concerns	Major concerns	No concerns	No concerns	Low	["Imprecision"]
B+M:P+C	0	No concerns	Low risk	No concerns	Major concerns	No concerns	No concerns	Low	["Imprecision"]
B+M+P:C	0	No concerns	Low risk	No concerns	Major concerns	No concerns	No concerns	Low	["Imprecision"]
B+M+P:C+K	0	No concerns	Low risk	No concerns	Major concerns	No concerns	No concerns	Low	["Imprecision"]
B+M+P:K	0	No concerns	Low risk	No concerns	Major concerns	No concerns	No concerns	Low	["Imprecision"]
B+M+P:M	0	No concerns	Low risk	No concerns	Major concerns	No concerns	No concerns	Low	["Imprecision"]
B+M+P:M+K	0	No concerns	Low risk	No concerns	Major concerns	No concerns	No concerns	Low	["Imprecision"]
B+M+P:M+P	0	No concerns	Low risk	No concerns	Major concerns	No concerns	No concerns	Low	["Imprecision"]
B+M+P:P	0	No concerns	Low risk	No concerns	Major concerns	No concerns	No concerns	Low	["Imprecision"]
B+M+P:P+C	0	No concerns	Low risk	No concerns	Major concerns	No concerns	No concerns	Low	["Imprecision"]
C:M	0	Some concerns	Low risk	No concerns	Major concerns	No concerns	No concerns	Low	["Within-study bias","Imprecision"]
C:M+K	0	Some concerns	Low risk	No concerns	Major concerns	No concerns	No concerns	High	["Within-study bias","Imprecision"]
C:M+P	0	Some concerns	Low risk	No concerns	No concerns	No concerns	No concerns	High	[]
C+K:M	0	Some concerns	Low risk	No concerns	Major concerns	No concerns	No concerns	Low	["Within-study bias","Imprecision"]
C+K:M+K	0	Some concerns	Low risk	No concerns	Major concerns	No concerns	No concerns	Low	["Within-study bias","Imprecision"]
C+K:M+P	0	Some concerns	Low risk	No concerns	Major concerns	No concerns	No concerns	Low	["Within-study bias","Imprecision"]
C+K:P	0	Some concerns	Low risk	No concerns	Some concerns	No concerns	No concerns	Moderate	["Within-study bias","Imprecision"]
K:M+P	0	Some concerns	Low risk	No concerns	No concerns	No concerns	No concerns	High	[]
M:M+K	0	No concerns	Low risk	No concerns	Major concerns	No concerns	No concerns	Low	["Imprecision"]
M:M+P	0	Some concerns	Low risk	No concerns	No concerns	No concerns	No concerns	High	[]
M:P	0	Some concerns	Low risk	No concerns	No concerns	No concerns	No concerns	High	[]
M:P+C	0	No concerns	Low risk	No concerns	No concerns	No concerns	No concerns	High	[]
M+K:M+P	0	Some concerns	Low risk	No concerns	No concerns	Some concerns	No concerns	Moderate	["Within-study bias","Heterogeneity"]
M+K:P	0	No concerns	Low risk	No concerns	No concerns	No concerns	No concerns	High	[]
M+K:P+C	0	No concerns	Low risk	No concerns	No concerns	No concerns	No concerns	High	[]
M+P:P+C	0	Some concerns	Low risk	No concerns	Major concerns	No concerns	No concerns	Low	["Within-study bias","Imprecision"]

Supplementary Table S9. CINEMA analysis for TRAE (OR)

Comparison	Number of studies	Within-study bias	Reporting bias	Indirectness	Imprecision	Heterogeneity	Incoherence	Confidence rating	Reason(s) for downgrading
B:B+M	5	Some concerns	Low risk	No concerns	Major concerns	No concerns	No concerns	Low	["Within-study bias","Imprecision"]
B:K	2	No concerns	Low risk	No concerns	No concerns	Major concerns	No concerns	Low	["Heterogeneity"]
B+M:B+M+P	3	No concerns	Low risk	No concerns	No concerns	Some concerns	No concerns	Moderate	["Heterogeneity"]
C:C+K	1	Some concerns	Low risk	No concerns	Some concerns	Some concerns	No concerns	Low	["Within-study bias","Imprecision","Heterogeneity"]
C:K	1	Some concerns	Low risk	No concerns	Major concerns	No concerns	No concerns	Low	["Within-study bias","Imprecision"]
C:P	2	Some concerns	Low risk	No concerns	Major concerns	No concerns	No concerns	Low	["Within-study bias","Imprecision"]
C:P+C	2	Some concerns	Low risk	No concerns	Some concerns	Some concerns	No concerns	Low	["Within-study bias","Imprecision","Heterogeneity"]
C+K:K	2	Some concerns	Low risk	No concerns	No concerns	Major concerns	No concerns	Low	["Within-study bias","Heterogeneity"]
C+K:P+C	1	Some concerns	Low risk	No concerns	Major concerns	No concerns	No concerns	Low	["Within-study bias","Imprecision"]
K:M	3	No concerns	Some concerns	No concerns	No concerns	No concerns	No concerns	Moderate	["Reporting bias"]
K:M+K	2	No concerns	Some concerns	No concerns	Major concerns	No concerns	No concerns	Low	["Reporting bias","Imprecision"]
K:P	3	No concerns	Low risk	No concerns	Major concerns	No concerns	No concerns	Low	[]
K:P+C	1	Some concerns	Low risk	No concerns	No concerns	Major concerns	Major concerns	Very low	["Within-study bias","Heterogeneity","Incoherence"]
M+P:P	1	Major concerns	Low risk	No concerns	No concerns	No concerns	No concerns	Low	["Within-study bias"]
P:P+C	2	Some concerns	Some concerns	No concerns	No concerns	Major concerns	Major concerns	Very low	["Within-study bias","Reporting bias","Heterogeneity","Incoherence"]
B:B+M+P	0	Some concerns	Low risk	No concerns	Some concerns	Some concerns	No concerns	Low	["Within-study bias","Imprecision","Heterogeneity"]
B:C	0	Some concerns	Low risk	No concerns	Major concerns	No concerns	No concerns	Low	["Within-study bias","Imprecision"]
B:C+K	0	No concerns	Low risk	No concerns	Major concerns	No concerns	No concerns	Low	["Imprecision"]
B:M	0	No concerns	Low risk	No concerns	Major concerns	No concerns	No concerns	Low	["Imprecision"]
B:M+K	0	No concerns	Low risk	No concerns	Major concerns	No concerns	No concerns	Low	["Imprecision"]
B:M+P	0	Some concerns	Low risk	No concerns	Some concerns	Some concerns	No concerns	Low	["Within-study bias","Imprecision","Heterogeneity"]
B:P	0	No concerns	Low risk	No concerns	Some concerns	Some concerns	No concerns	Moderate	["Imprecision","Heterogeneity"]
B:P+C	0	No concerns	Low risk	No concerns	Major concerns	No concerns	No concerns	Low	["Imprecision"]
B+M:C	0	Some concerns	Low risk	No concerns	Major concerns	No concerns	No concerns	Low	["Within-study bias","Imprecision"]
B+M:C+K	0	Some concerns	Low risk	No concerns	Major concerns	No concerns	No concerns	Low	["Within-study bias","Imprecision"]
B+M:K	0	Some concerns	Low risk	No concerns	Major concerns	No concerns	No concerns	Low	[]
B+M:M	0	Some concerns	Low risk	No concerns	Major concerns	No concerns	No concerns	Low	["Within-study bias","Imprecision"]
B+M:M+K	0	No concerns	Low risk	No concerns	Major concerns	No concerns	No concerns	Low	["Imprecision"]
B+M:M+P	0	Some concerns	Low risk	No concerns	Some concerns	Some concerns	No concerns	Low	["Within-study bias","Imprecision","Heterogeneity"]
B+M:P	0	Some concerns	Low risk	No concerns	Major concerns	No concerns	No concerns	Low	["Within-study bias","Imprecision"]
B+M:P+C	0	Some concerns	Low risk	No concerns	Major concerns	No concerns	No concerns	Low	["Within-study bias","Imprecision"]
B+M+P:C	0	Some concerns	Low risk	No concerns	No concerns	Some concerns	No concerns	Moderate	["Within-study bias","Heterogeneity"]
B+M+P:C+K	0	No concerns	Low risk	No concerns	Major concerns	No concerns	No concerns	Low	["Imprecision"]
B+M+P:K	0	No concerns	Low risk	No concerns	No concerns	No concerns	No concerns	High	[]
B+M+P:M	0	No concerns	Low risk	No concerns	Major concerns	No concerns	No concerns	Low	["Imprecision"]
B+M+P:M+K	0	No concerns	Low risk	No concerns	Major concerns	No concerns	No concerns	Low	["Imprecision"]
B+M+P:M+P	0	Some concerns	Low risk	No concerns	Major concerns	No concerns	No concerns	Low	["Within-study bias","Imprecision"]
B+M+P:P	0	No concerns	Low risk	No concerns	No concerns	No concerns	No concerns	High	[]
B+M+P:P+C	0	Some concerns	Low risk	No concerns	Major concerns	No concerns	No concerns	Low	["Within-study bias","Imprecision"]
C:M	0	Some concerns	Low risk	No concerns	No concerns	No concerns	No concerns	Moderate	["Within-study bias"]
C:M+K	0	Some concerns	Low risk	No concerns	Major concerns	No concerns	No concerns	High	["Within-study bias","Imprecision"]
C:M+P	0	Some concerns	Low risk	No concerns	No concerns	No concerns	No concerns	Moderate	["Within-study bias"]
C+K:M	0	Some concerns	Low risk	No concerns	Major concerns	No concerns	No concerns	Low	["Within-study bias","Imprecision"]
C+K:M+K	0	No concerns	Low risk	No concerns	Major concerns	No concerns	No concerns	Low	["Imprecision"]
C+K:M+P	0	Some concerns	Low risk	No concerns	Some concerns	Some concerns	No concerns	Low	["Within-study bias","Imprecision","Heterogeneity"]
C+K:P	0	Some concerns	Low risk	No concerns	Some concerns	Some concerns	No concerns	Low	["Within-study bias","Imprecision","Heterogeneity"]
K:M+P	0	Some concerns	Low risk	No concerns	No concerns	No concerns	No concerns	High	["Within-study bias"]
M:M+K	0	No concerns	Low risk	No concerns	Major concerns	No concerns	No concerns	Low	["Imprecision"]
M:M+P	0	Some concerns	Low risk	No concerns	Major concerns	No concerns	No concerns	Low	["Within-study bias","Imprecision"]
M:P	0	No concerns	Low risk	No concerns	No concerns	No concerns	No concerns	High	[]
M:P+C	0	Some concerns	Low risk	No concerns	Major concerns	No concerns	No concerns	Low	["Within-study bias","Imprecision"]
M+K:M+P	0	Some concerns	Low risk	No concerns	Major concerns	No concerns	No concerns	Low	["Within-study bias","Imprecision"]
M+K:P	0	No concerns	Low risk	No concerns	Major concerns	No concerns	No concerns	Low	["Imprecision"]
M+K:P+C	0	No concerns	Low risk	No concerns	Major concerns	No concerns	No concerns	Low	["Imprecision"]
M+P:P+C	0	Some concerns	Low risk	No concerns	No concerns	Major concerns	No concerns	Low	["Within-study bias","Heterogeneity"]

Supplementary Table S10. CINEMA analysis for SAE (OR)

Comparison	Number of studies	Within-study bias	Reporting bias	Indirectness	Imprecision	Heterogeneity	Incoherence	Confidence rating	Reason(s) for downgrading
B:B+M	4	Some concerns	Low risk	No concerns	No concerns	Major concerns	No concerns	Low	["Within-study bias","Heterogeneity"]
B:K	1	No concerns	Low risk	No concerns	No concerns	Some concerns	No concerns	High	[]
B+M:B+M+P	2	No concerns	Some concerns	No concerns	Major concerns	No concerns	No concerns	Low	["Reporting bias","Imprecision"]
C:K	1	Some concerns	Some concerns	No concerns	No concerns	Major concerns	No concerns	Very low	["Within-study bias","Reporting bias","Heterogeneity"]
C:P	1	Some concerns	Low risk	No concerns	Major concerns	No concerns	No concerns	Low	["Within-study bias","Imprecision"]
C:P+C	1	No concerns	Some concerns	No concerns	No concerns	No concerns	No concerns	High	[]
K:M	4	Some concerns	Low risk	No concerns	No concerns	Major concerns	No concerns	Low	["Within-study bias","Heterogeneity"]
K:M+K	1	No concerns	Some concerns	No concerns	No concerns	Major concerns	No concerns	Low	["Reporting bias","Heterogeneity"]
K:P	1	Some concerns	High risk	No concerns	Major concerns	No concerns	No concerns	Very low	["Within-study bias","Reporting bias","Imprecision"]
M+P:P	1	Major concerns	Low risk	No concerns	No concerns	Major concerns	No concerns	Very low	["Within-study bias","Heterogeneity"]
P:P+C	1	Some concerns	Low risk	No concerns	No concerns	No concerns	No concerns	High	[]
B:B+M+P	0	No concerns	Low risk	No concerns	No concerns	Major concerns	No concerns	Low	["Heterogeneity"]
B:C	0	Some concerns	Low risk	No concerns	Major concerns	No concerns	No concerns	Low	["Within-study bias","Imprecision"]
B:M	0	No concerns	Low risk	No concerns	Major concerns	No concerns	No concerns	Low	["Imprecision"]
B:M+K	0	No concerns	Some concerns	No concerns	Major concerns	No concerns	No concerns	Low	["Reporting bias","Imprecision"]
B:M+P	0	Some concerns	Low risk	No concerns	Major concerns	No concerns	No concerns	Low	["Within-study bias","Imprecision"]
B:P	0	No concerns	Low risk	No concerns	Some concerns	Some concerns	No concerns	Moderate	["Imprecision","Heterogeneity"]
B:P+C	0	No concerns	Low risk	No concerns	Major concerns	No concerns	No concerns	Low	["Imprecision"]
B+M:C	0	Some concerns	Low risk	No concerns	Major concerns	No concerns	No concerns	Low	["Within-study bias","Imprecision"]
B+M:K	0	No concerns	Low risk	No concerns	No concerns	No concerns	No concerns	High	[]
B+M:M	0	Some concerns	Low risk	No concerns	Major concerns	No concerns	No concerns	Low	["Within-study bias","Imprecision"]
B+M:M+K	0	No concerns	Some concerns	No concerns	Major concerns	No concerns	No concerns	Low	["Reporting bias","Imprecision"]
B+M:M+P	0	Some concerns	Low risk	No concerns	Major concerns	No concerns	No concerns	Low	["Within-study bias","Imprecision"]
B+M:P	0	Some concerns	Low risk	No concerns	No concerns	Major concerns	No concerns	Low	["Within-study bias","Heterogeneity"]
B+M:P+C	0	No concerns	Low risk	No concerns	Major concerns	No concerns	No concerns	Low	["Imprecision"]
B+M+P:C	0	No concerns	Low risk	No concerns	Some concerns	Some concerns	No concerns	Moderate	["Imprecision","Heterogeneity"]
B+M+P:K	0	No concerns	Low risk	No concerns	No concerns	No concerns	No concerns	High	[]
B+M+P:M	0	No concerns	Low risk	No concerns	No concerns	Major concerns	No concerns	Low	["Heterogeneity"]
B+M+P:M+K	0	No concerns	Low risk	No concerns	Major concerns	No concerns	No concerns	Low	["Imprecision"]
B+M+P:M+P	0	Some concerns	Low risk	No concerns	Major concerns	No concerns	No concerns	Low	["Within-study bias","Imprecision"]
B+M+P:P	0	No concerns	Low risk	No concerns	No concerns	Some concerns	No concerns	High	[]
B+M+P:P+C	0	No concerns	Low risk	No concerns	Major concerns	No concerns	No concerns	Low	["Imprecision"]
C:M	0	Some concerns	Low risk	No concerns	Major concerns	No concerns	No concerns	Low	["Within-study bias","Imprecision"]
C:M+K	0	Some concerns	Low risk	No concerns	Major concerns	No concerns	No concerns	Low	["Within-study bias","Imprecision"]
C:M+P	0	Major concerns	Low risk	No concerns	Major concerns	No concerns	No concerns	Very low	["Within-study bias","Imprecision"]
K:M+P	0	Some concerns	Low risk	No concerns	No concerns	Major concerns	No concerns	Low	["Within-study bias","Heterogeneity"]
K:P+C	0	Some concerns	Some concerns	No concerns	No concerns	No concerns	No concerns	Moderate	["Within-study bias","Reporting bias"]
M:M+K	0	No concerns	Low risk	No concerns	Major concerns	No concerns	No concerns	Low	["Imprecision"]
M:M+P	0	Some concerns	Low risk	No concerns	Major concerns	No concerns	No concerns	Low	["Within-study bias","Imprecision"]
M:P	0	Some concerns	Low risk	No concerns	Major concerns	No concerns	No concerns	Low	["Within-study bias","Imprecision"]
M:P+C	0	Some concerns	Low risk	No concerns	No concerns	Major concerns	No concerns	Low	["Within-study bias","Heterogeneity"]
M+K:M+P	0	Some concerns	Low risk	No concerns	Major concerns	No concerns	No concerns	Low	["Within-study bias","Imprecision"]
M+K:P	0	No concerns	Low risk	No concerns	Major concerns	No concerns	No concerns	Low	["Imprecision"]
M+K:P+C	0	No concerns	Low risk	No concerns	Major concerns	No concerns	No concerns	Low	["Imprecision"]
M+P:P+C	0	Some concerns	Low risk	No concerns	Major concerns	No concerns	No concerns	Low	["Within-study bias","Imprecision"]

Supplementary Table S11. CINEMA analysis for grade 3≤ AE (OR)

Comparison	Number of studies	Within-study bias	Reporting bias	Indirectness	Imprecision	Heterogeneity	Incoherence	Confidence rating	Reason(s) for downgrading
B:B+M	5	Some concerns	Low risk	No concerns	Major concerns	No concerns	No concerns	Low	["Within-study bias","Imprecision"]
B:K	1	No concerns	Some concerns	No concerns	No concerns	Some concerns	No concerns	Moderate	["Reporting bias","Heterogeneity"]
B+M:B+M+P	3	No concerns	Low risk	No concerns	No concerns	Major concerns	No concerns	Low	["Heterogeneity"]
C:C+K	1	Some concerns	Low risk	No concerns	No concerns	Some concerns	No concerns	Moderate	["Within-study bias","Heterogeneity"]
C:K	1	Some concerns	Low risk	No concerns	Major concerns	No concerns	No concerns	Low	["Within-study bias","Imprecision"]
C:P	2	Some concerns	Low risk	No concerns	Major concerns	No concerns	No concerns	Low	["Within-study bias","Imprecision"]
C:P+C	2	Some concerns	Low risk	No concerns	No concerns	Major concerns	Major concerns	Very low	["Within-study bias","Heterogeneity","Incoherence"]
C+K:K	2	No concerns	Low risk	No concerns	No concerns	No concerns	No concerns	High	[]
C+K:P+C	1	Some concerns	Low risk	No concerns	Major concerns	No concerns	Major concerns	Very low	["Within-study bias","Imprecision","Incoherence"]
K:M	3	Some concerns	Some concerns	No concerns	No concerns	Some concerns	No concerns	Low	["Within-study bias","Reporting bias","Heterogeneity"]
K:M+K	2	No concerns	Low risk	No concerns	No concerns	No concerns	No concerns	High	[]
K:P	3	Some concerns	Low risk	No concerns	Major concerns	No concerns	No concerns	Low	["Within-study bias","Imprecision"]
K:P+C	1	Some concerns	Low risk	No concerns	No concerns	Some concerns	Major concerns	Low	["Within-study bias","Heterogeneity","Incoherence"]
M+P:P	1	Major concerns	Low risk	No concerns	No concerns	Some concerns	No concerns	Low	["Within-study bias","Heterogeneity"]
P:P+C	2	Some concerns	Low risk	No concerns	No concerns	No concerns	No concerns	High	[]
B:B+M+P	0	Some concerns	Low risk	No concerns	No concerns	Major concerns	No concerns	Low	["Within-study bias","Heterogeneity"]
B:C	0	Some concerns	Low risk	No concerns	No concerns	Major concerns	No concerns	Low	["Within-study bias","Heterogeneity"]
B:C+K	0	No concerns	Low risk	No concerns	Major concerns	No concerns	No concerns	Low	["Imprecision"]
B:M	0	No concerns	Low risk	No concerns	Major concerns	No concerns	No concerns	Low	["Imprecision"]
B:M+K	0	No concerns	Low risk	No concerns	Major concerns	No concerns	No concerns	Low	["Imprecision"]
B:M+P	0	Some concerns	Low risk	No concerns	Major concerns	No concerns	No concerns	Low	["Within-study bias","Imprecision"]
B:P	0	No concerns	Low risk	No concerns	No concerns	No concerns	No concerns	High	[]
B:P+C	0	No concerns	Low risk	No concerns	Major concerns	No concerns	No concerns	Low	["Imprecision"]
B+M:C	0	Some concerns	Low risk	No concerns	No concerns	Some concerns	No concerns	Moderate	["Within-study bias","Heterogeneity"]
B+M:C+K	0	Some concerns	Low risk	No concerns	Major concerns	No concerns	No concerns	Low	["Within-study bias","Imprecision"]
B+M:K	0	Some concerns	Low risk	No concerns	No concerns	No concerns	No concerns	Moderate	["Within-study bias"]
B+M:M	0	Some concerns	Low risk	No concerns	Major concerns	No concerns	No concerns	Low	["Within-study bias","Imprecision"]
B+M:M+K	0	No concerns	Low risk	No concerns	Major concerns	No concerns	No concerns	Low	["Imprecision"]
B+M:M+P	0	Some concerns	Low risk	No concerns	Major concerns	No concerns	No concerns	Low	["Within-study bias","Imprecision"]
B+M:P	0	Some concerns	Low risk	No concerns	No concerns	No concerns	No concerns	Moderate	["Within-study bias"]
B+M:P+C	0	Some concerns	Low risk	No concerns	Major concerns	No concerns	No concerns	Low	["Within-study bias","Imprecision"]
B+M+P:C	0	Some concerns	Low risk	No concerns	No concerns	No concerns	No concerns	Moderate	["Within-study bias"]
B+M+P:C+K	0	No concerns	Low risk	No concerns	Major concerns	No concerns	No concerns	Low	["Imprecision"]
B+M+P:K	0	No concerns	Low risk	No concerns	No concerns	No concerns	No concerns	High	[]
B+M+P:M	0	No concerns	Low risk	No concerns	Some concerns	Some concerns	No concerns	Moderate	["Imprecision","Heterogeneity"]
B+M+P:M+K	0	No concerns	Low risk	No concerns	Major concerns	No concerns	No concerns	Low	["Imprecision"]
B+M+P:M+P	0	Some concerns	Low risk	No concerns	Major concerns	No concerns	No concerns	Low	["Within-study bias","Imprecision"]
B+M+P:P	0	No concerns	Low risk	No concerns	No concerns	No concerns	No concerns	High	[]
B+M+P:P+C	0	Some concerns	Low risk	No concerns	Some concerns	Some concerns	No concerns	Low	["Within-study bias","Imprecision","Heterogeneity"]
C:M	0	Some concerns	Low risk	No concerns	No concerns	Major concerns	No concerns	Low	["Within-study bias","Heterogeneity"]
C:M+K	0	Some concerns	Low risk	No concerns	No concerns	No concerns	No concerns	Moderate	["Within-study bias"]
C:M+P	0	Some concerns	Low risk	No concerns	Some concerns	Some concerns	No concerns	Low	["Within-study bias","Imprecision","Heterogeneity"]
C+K:M	0	Some concerns	Low risk	No concerns	Major concerns	No concerns	No concerns	Low	["Within-study bias","Imprecision"]
C+K:M+K	0	No concerns	Low risk	No concerns	Major concerns	No concerns	No concerns	Low	["Imprecision"]
C+K:M+P	0	Some concerns	Low risk	No concerns	Major concerns	No concerns	No concerns	Low	["Within-study bias","Imprecision"]
C+K:P	0	Some concerns	Low risk	No concerns	No concerns	No concerns	No concerns	Moderate	["Within-study bias"]
K:M+P	0	Some concerns	Low risk	No concerns	No concerns	Major concerns	No concerns	Low	["Within-study bias","Heterogeneity"]
M:M+K	0	No concerns	Low risk	No concerns	Major concerns	No concerns	No concerns	Low	["Imprecision"]
M:M+P	0	Some concerns	Low risk	No concerns	Major concerns	No concerns	No concerns	Low	["Within-study bias","Imprecision"]
M:P	0	Some concerns	Low risk	No concerns	No concerns	No concerns	No concerns	Moderate	["Within-study bias"]
M:P+C	0	Some concerns	Low risk	No concerns	Major concerns	No concerns	No concerns	Low	["Within-study bias","Imprecision"]
M+K:M+P	0	Some concerns	Low risk	No concerns	Major concerns	No concerns	No concerns	Low	["Within-study bias","Imprecision"]
M+K:P	0	No concerns	Low risk	No concerns	No concerns	No concerns	No concerns	High	[]
M+K:P+C	0	No concerns	Low risk	No concerns	Major concerns	No concerns	No concerns	Low	["Imprecision"]
M+P:P+C	0	Some concerns	Low risk	No concerns	Major concerns	No concerns	No concerns	Low	["Within-study bias","Imprecision"]

Supplementary Table S12. CINEMA analysis for TDR (OR)

Comparison	Number of studies	Within-study bias	Reporting bias	Indirectness	Imprecision	Heterogeneity	Incoherence	Confidence rating	Reason(s) for downgrading
B:B+M	5	Some concerns	Low risk	No concerns	No concerns	No concerns	No concerns	High	[]
B:K	2	No concerns	Low risk	No concerns	Major concerns	No concerns	No concerns	Low	["Imprecision"]
B+M:B+M+P	3	No concerns	Low risk	No concerns	Major concerns	No concerns	No concerns	Low	["Imprecision"]
C:C+K	1	Some concerns	Low risk	No concerns	Major concerns	No concerns	No concerns	Low	["Within-study bias","Imprecision"]
C:K	1	Some concerns	Low risk	No concerns	No concerns	Some concerns	No concerns	Moderate	["Within-study bias","Heterogeneity"]
C:P	2	Some concerns	Low risk	No concerns	No concerns	No concerns	No concerns	High	[]
C:P+C	2	No concerns	Low risk	No concerns	No concerns	No concerns	No concerns	High	[]
C+K:K	2	Some concerns	Low risk	No concerns	Major concerns	No concerns	No concerns	Low	["Within-study bias","Imprecision"]
C+K:P+C	1	Some concerns	Low risk	No concerns	No concerns	No concerns	No concerns	High	[]
K:M	4	Some concerns	Low risk	No concerns	Major concerns	No concerns	No concerns	Low	["Within-study bias","Imprecision"]
K:M+K	3	No concerns	Low risk	No concerns	Major concerns	No concerns	No concerns	Low	["Imprecision"]
K:P	3	Some concerns	Low risk	No concerns	No concerns	No concerns	No concerns	High	[]
K:P+C	1	No concerns	Low risk	No concerns	No concerns	No concerns	No concerns	High	[]
M+P:P	1	Some concerns	Low risk	No concerns	Major concerns	No concerns	No concerns	Low	["Within-study bias","Imprecision"]
P:P+C	1	No concerns	Low risk	No concerns	Some concerns	No concerns	No concerns	Moderate	["Imprecision"]
B:B+M+P	0	No concerns	Low risk	No concerns	No concerns	No concerns	No concerns	High	[]
B:C	0	No concerns	Low risk	No concerns	Major concerns	No concerns	No concerns	Low	["Imprecision"]
B:C+K	0	No concerns	Low risk	No concerns	Major concerns	No concerns	No concerns	Low	["Imprecision"]
B:M	0	No concerns	Low risk	No concerns	Major concerns	No concerns	No concerns	Low	["Imprecision"]
B:M+K	0	No concerns	Low risk	No concerns	Major concerns	No concerns	No concerns	Low	["Imprecision"]
B:M+P	0	Some concerns	Low risk	No concerns	Major concerns	No concerns	No concerns	Low	["Within-study bias","Imprecision"]
B:P	0	No concerns	Low risk	No concerns	Major concerns	No concerns	No concerns	Low	["Imprecision"]
B:P+C	0	No concerns	Low risk	No concerns	Major concerns	No concerns	No concerns	Low	["Imprecision"]
B+M:C	0	Some concerns	Low risk	No concerns	Major concerns	No concerns	No concerns	Low	["Within-study bias","Imprecision"]
B+M:C+K	0	Some concerns	Low risk	No concerns	Major concerns	No concerns	No concerns	Low	["Within-study bias","Imprecision"]
B+M:K	0	No concerns	Low risk	No concerns	Major concerns	No concerns	No concerns	Low	["Imprecision"]
B+M:M	0	No concerns	Low risk	No concerns	Major concerns	No concerns	No concerns	Low	["Imprecision"]
B+M:M+K	0	No concerns	Low risk	No concerns	Major concerns	No concerns	No concerns	Low	["Imprecision"]
B+M:M+P	0	Some concerns	Low risk	No concerns	Major concerns	No concerns	No concerns	Low	["Within-study bias","Imprecision"]
B+M:P	0	No concerns	Low risk	No concerns	Major concerns	No concerns	No concerns	Low	["Imprecision"]
B+M:P+C	0	No concerns	Low risk	No concerns	Major concerns	No concerns	No concerns	Low	["Imprecision"]
B+M+P:C	0	No concerns	Low risk	No concerns	Major concerns	No concerns	No concerns	Low	["Imprecision"]
B+M+P:C+K	0	No concerns	Low risk	No concerns	Major concerns	No concerns	No concerns	Low	["Imprecision"]
B+M+P:K	0	No concerns	Low risk	No concerns	Major concerns	No concerns	No concerns	Low	["Imprecision"]
B+M+P:M	0	No concerns	Low risk	No concerns	Major concerns	No concerns	No concerns	Low	["Imprecision"]
B+M+P:M+K	0	No concerns	Low risk	No concerns	Major concerns	No concerns	No concerns	Low	["Imprecision"]
B+M+P:M+P	0	No concerns	Low risk	No concerns	Major concerns	No concerns	No concerns	Low	["Imprecision"]
B+M+P:P	0	No concerns	Low risk	No concerns	Major concerns	No concerns	No concerns	Low	["Imprecision"]
B+M+P:P+C	0	No concerns	Low risk	No concerns	Major concerns	No concerns	No concerns	Low	["Imprecision"]
C:M	0	Some concerns	Low risk	No concerns	Major concerns	No concerns	No concerns	Low	["Within-study bias","Imprecision"]
C:M+K	0	Some concerns	Low risk	No concerns	Major concerns	No concerns	No concerns	High	["Within-study bias","Imprecision"]
C:M+P	0	Some concerns	Low risk	No concerns	No concerns	No concerns	No concerns	High	[]
C+K:M	0	Some concerns	Low risk	No concerns	Major concerns	No concerns	No concerns	Low	["Within-study bias","Imprecision"]
C+K:M+K	0	Some concerns	Low risk	No concerns	Major concerns	No concerns	No concerns	Low	["Within-study bias","Imprecision"]
C+K:M+P	0	Some concerns	Low risk	No concerns	Major concerns	No concerns	No concerns	Low	["Within-study bias","Imprecision"]
C+K:P	0	Some concerns	Low risk	No concerns	Some concerns	No concerns	No concerns	Moderate	["Within-study bias","Imprecision"]
K:M+P	0	Some concerns	Low risk	No concerns	No concerns	No concerns	No concerns	High	[]
M:M+K	0	No concerns	Low risk	No concerns	Major concerns	No concerns	No concerns	Low	["Imprecision"]
M:M+P	0	Some concerns	Low risk	No concerns	No concerns	No concerns	No concerns	High	[]
M:P	0	Some concerns	Low risk	No concerns	No concerns	No concerns	No concerns	High	[]
M:P+C	0	No concerns	Low risk	No concerns	No concerns	No concerns	No concerns	High	[]
M+K:M+P	0	Some concerns	Low risk	No concerns	No concerns	Some concerns	No concerns	Moderate	["Within-study bias","Heterogeneity"]
M+K:P	0	No concerns	Low risk	No concerns	No concerns	No concerns	No concerns	High	[]
M+K:P+C	0	No concerns	Low risk	No concerns	No concerns	No concerns	No concerns	High	[]
M+P:P+C	0	Some concerns	Low risk	No concerns	Major concerns	No concerns	No concerns	Low	["Within-study bias","Imprecision"]

Supplementary Table S13. GRADE assessment for PFS and OS (HR).

Outcomes	№ of participants (studies)	Certainty of the evidence (GRADE)	Relative effect (95% CI)	Outcomes	№ of participants (studies)	Certainty of the evidence (GRADE)	Relative effect* (95% CI)
**B+M+P compared to B+M**				**B+M+P compared to B**			
PFS (HR)	1166 (3 RCTs)	⊕⊕⊕◯ Moderate	**HR 0.73** (0.55 to 0.97)	PFS (HR)	1640 (0 RCTs)	⊕⊕◯◯ Low	**HR 0.41** (0.29 to 0.58)
OS (HR)	1166 (3 RCTs)	⊕⊕◯◯ Low	**HR 0.79** (0.64 to 0.98)	OS (HR)	1640 (0 RCTs)	⊕◯◯◯ Very low	**HR 0.54** (0.42 to 0.70)
**B+M+P compared to P+C**				**B+M+P compared to P**			
PFS (HR)	1018 (0 RCTs)	⊕◯◯◯ Very low	**HR 0.30** (0.15 to 0.57)	PFS (HR)	2338 (0 RCTs)	⊕◯◯◯ Very low	**HR 0.21** (0.12 to 0.38)
OS (HR)	1053 (0 RCTs)	⊕◯◯◯ Very low	**HR 0.85** (0.55 to 1.31)	OS (HR)	2363 (0 RCTs)	⊕◯◯◯ Very low	**HR 0.67** (0.45 to 0.99)
**B+M+P compared to M+P**				**B+M+P compared to M**			
PFS (HR)	805 (0 RCTs)	⊕⊕⊕◯ Moderate	**HR 0.18** (0.08 to 0.39)	PFS (HR)	1300 (0 RCTs)	⊕⊕⊕⊕ High	**HR 0.16** (0.09 to 0.29)
OS (HR)	805 (0 RCTs)	⊕◯◯◯ Very low	**HR 0.63** (0.35 to 1.16)	OS (HR)	1300 (0 RCTs)	⊕⊕⊕◯ Moderate	**HR 0.44** (0.30 to 0.66)

Outcome	Participants (studies)	Certainty	Effect
**B+M+P compared to C+K**			
PFS (HR)	833 (0 RCTs)	⊕⊕⊕◯ Moderate	**HR 0.15** (0.08 to 0.29)
OS (HR)	894 (0 RCTs)	⊕◯◯◯ Very low	**HR 0.58** (0.38 to 0.88)
**B+M+P compared to M+K**			
PFS (HR)	705 (0 RCTs)	⊕⊕⊕◯ Moderate	**HR 0.15** (0.08 to 0.29)
OS (HR)	705 (0 RCTs)	⊕⊕◯◯ Low	**HR 0.41** (0.26 to 0.64)
**B+M compared to B**			
PFS (HR)	2113 (5 RCTs)	⊕⊕⊕◯ Moderate	**HR 0.55** (0.45 to 0.69)
OS (HR)	2113 (5 RCTs)	⊕⊕⊕◯ Moderate	**HR 0.69** (0.59 to 0.80)
**B+M compared to P**			
PFS (HR)	2811 (0 RCTs)	⊕⊕◯◯ Low	**HR 0.28** (0.17 to 0.48)
OS (HR)	2836 (0 RCTs)	⊕◯◯◯ Very low	**HR 0.85** (0.61 to 1.19)
**B+M compared to M**			
PFS (HR)	1773 (0 RCTs)	⊕⊕⊕◯ Moderate	**HR 0.22** (0.13 to 0.36)
OS (HR)	1773 (0 RCTs)	⊕⊕◯◯ Low	**HR 0.56** (0.40 to 0.78)
**B+M compared to M+K**			
PFS (HR)	1178 (0 RCTs)	⊕⊕◯◯ Low	**HR 0.21** (0.12 to 0.36)
OS (HR)	1178 (0 RCTs)	⊕◯◯◯ Very low	**HR 0.52** (0.35 to 0.77)
**B+M compared to K**			
PFS (HR)	2684 (0 RCTs)	⊕⊕⊕◯ Moderate	**HR 0.15** (0.10 to 0.24)
OS (HR)	3075 (0 RCTs)	⊕⊕◯◯ Low	**HR 0.55** (0.41 to 0.73)
**B compared to P**			
PFS (HR)	2812 (0 RCTs)	⊕◯◯◯ Very low	**HR 0.51** (0.32 to 0.81)
**P compared to B**			
OS (HR)	2837 (0 RCTs)	⊕◯◯◯ Very low	**HR 0.81** (0.61 to 1.08)
**B compared to M**			
PFS (HR)	1774 (0 RCTs)	⊕⊕⊕◯ Moderate	**HR 0.39** (0.25 to 0.63)
OS (HR)	1774 (0 RCTs)	⊕⊕◯◯ Low	**HR 0.81** (0.59 to 1.09)
**B compared to M+K**			
PFS (HR)	1179 (0 RCTs)	⊕⊕◯◯ Low	**HR 0.37** (0.22 to 0.63)
OS (HR)	1179 (0 RCTs)	⊕◯◯◯ Very low	**HR 0.76** (0.53 to 1.08)

Outcome	Participants (studies)	Certainty	Effect
**B+M+P compared to C**			
PFS (HR)	1222 (0 RCTs)	⊕⊕⊕◯ Moderate	**HR 0.12** (0.06 to 0.22)
OS (HR)	1587 (0 RCTs)	⊕⊕◯◯ Low	**HR 0.47** (0.32 to 0.70)
**B+M+P compared to K**			
PFS (HR)	2211 (0 RCTs)	⊕⊕⊕◯ Moderate	**HR 0.11** (0.07 to 0.19)
OS (HR)	2602 (0 RCTs)	⊕⊕◯◯ Low	**HR 0.44** (0.30 to 0.62)
**B+M compared to P+C**			
PFS (HR)	1491 (0 RCTs)	⊕⊕◯◯ Low	**HR 0.40** (0.22 to 0.73)
**P+C compared to B+M**			
OS (HR)	1526 (0 RCTs)	⊕◯◯◯ Very low	**HR 0.92** (0.63 to 1.35)
**B+M compared to M+P**			
PFS (HR)	1278 (0 RCTs)	⊕⊕◯◯ Low	**HR 0.25** (0.12 to 0.50)
OS (HR)	1278 (0 RCTs)	⊕◯◯◯ Very low	**HR 0.80** (0.46 to 1.42)
**B+M compared to C+K**			
PFS (HR)	1306 (0 RCTs)	⊕◯◯◯ Very low	**HR 0.21** (0.12 to 0.37)
OS (HR)	1367 (0 RCTs)	⊕◯◯◯ Very low	**HR 0.73** (0.51 to 1.05)
**B+M compared to C**			
PFS (HR)	1695 (0 RCTs)	⊕⊕⊕◯ Moderate	**HR 0.16** (0.09 to 0.28)
OS (HR)	2060 (0 RCTs)	⊕◯◯◯ Very low	**HR 0.60** (0.43 to 0.84)
**B compared to P+C**			
PFS (HR)	1492 (0 RCTs)	⊕◯◯◯ Very low	**HR 0.73** (0.41 to 1.26)
**P+C compared to B**			
OS (HR)	1527 (0 RCTs)	⊕⊕◯◯ Low	**HR 0.64** (0.45 to 0.90)
**B compared to M+P**			
PFS (HR)	1279 (0 RCTs)	⊕⊕◯◯ Low	**HR 0.44** (0.23 to 0.87)
**M+P compared to B**			
OS (HR)	1279 (0 RCTs)	⊕◯◯◯ Very low	**HR 0.86** (0.50 to 1.48)
**B compared to C+K**			
PFS (HR)	1307 (0 RCTs)	⊕◯◯◯ Very low	**HR 0.38** (0.22 to 0.64)
**C+K compared to B**			
OS (HR)	1368 (0 RCTs)	⊕◯◯◯ Very low	**HR 0.94** (0.68 to 1.31)
**B compared to C**			
PFS (HR)	1696 (0 RCTs)	⊕⊕◯◯ Low	**HR 0.28** (0.17 to 0.49)
OS (HR)	2061 (0 RCTs)	⊕◯◯◯ Very low	**HR 0.87** (0.64 to 1.17)

**B compared to K**			
PFS (HR)	2685<br>(2 RCTs)	⊕⊕⊕⊕<br>High	**HR 0.27**<br>(0.18 to 0.40)
OS (HR)	3076<br>(2 RCTs)	⊕⊕⊕◯<br>Moderate	**HR 0.79**<br>(0.63 to 1.01)
**P+C compared to M+P**			
PFS (HR)	657<br>(0 RCTs)	⊕⊕◯◯<br>Low	**HR 0.61**<br>(0.34 to 1.11)
OS (HR)	692<br>(0 RCTs)	⊕⊕◯◯<br>Low	**HR 0.74**<br>(0.44 to 1.23)
**P+C compared to C+K**			
PFS (HR)	685<br>(1 RCT)	⊕⊕⊕◯<br>Moderate	**HR 0.52**<br>(0.32 to 0.84)
OS (HR)	781<br>(1 RCT)	⊕⊕⊕◯<br>Moderate	**HR 0.68**<br>(0.50 to 0.92)
**P+C compared to C**			
PFS (HR)	1074<br>(2 RCTs)	⊕⊕⊕⊕<br>High	**HR 0.39**<br>(0.28 to 0.54)
OS (HR)	1474<br>(2 RCTs)	⊕⊕⊕⊕<br>High	**HR 0.39**<br>(0.28 to 0.54)
**P compared to M+P**			
PFS (HR)	1977<br>(1 RCT)	⊕⊕⊕◯<br>Moderate	**HR 0.87**<br>(0.54 to 1.40)
OS (HR)	2002<br>(1 RCT)	⊕⊕⊕◯<br>Moderate	**HR 0.94**<br>(0.59 to 1.49)
**P compared to M+K**			
PFS (HR)	1877<br>(0 RCTs)	⊕◯◯◯<br>Very low	**HR 0.73**<br>(0.47 to 1.13)
OS (HR)	1902<br>(0 RCTs)	⊕◯◯◯<br>Very low	**HR 0.61**<br>(0.45 to 0.84)
**P compared to C**			
PFS (HR)	2394<br>(2 RCTs)	⊕⊕⊕⊕<br>High	**HR 0.55**<br>(0.41 to 0.75)
OS (HR)	2784<br>(2 RCTs)	⊕⊕⊕⊕<br>High	**HR 0.70**<br>(0.60 to 0.83)
**M+P compared to M+K**			
PFS (HR)	344<br>(0 RCTs)	⊕◯◯◯<br>Very low	**HR 0.84**<br>(0.44 to 1.62)
OS (HR)	344<br>(0 RCTs)	⊕◯◯◯<br>Very low	**HR 0.65**<br>(0.37 to 1.14)
**M+P compared to C+K**			
PFS (HR)	472<br>(0 RCTs)	⊕◯◯◯<br>Very low	**HR 0.85**<br>(0.44 to 1.26)
OS (HR)	533<br>(0 RCTs)	⊕◯◯◯<br>Very low	**HR 0.91**<br>(0.54 to 1.54)
**M+P compared to K**			
PFS (HR)	1850<br>(0 RCTs)	⊕◯◯◯<br>Very low	**HR 0.62**<br>(0.36 to 1.06)
OS (HR)	2241<br>(0 RCTs)	⊕◯◯◯<br>Very low	**HR 0.68**<br>(0.42 to 1.12)
**C+K compared to M**			
OS (HR)	1028<br>(0 RCTs)	⊕◯◯◯<br>Very low	**HR 0.76**<br>(0.57 to 1.02)
**M compared to C+K**			
PFS (HR)	967<br>(0 RCTs)	⊕◯◯◯<br>Very low	**HR 0.95**<br>(0.62 to 1.48)
**C+K compared to M+K**			
PFS (HR)	372	⊕◯◯◯	**HR 0.99**

**P+C compared to P**			
PFS (HR)	2190<br>(1 RCT)	⊕⊕⊕⊕<br>High	**HR 0.70**<br>(0.50 to 0.99)
OS (HR)	2250<br>(2 RCTs)	⊕⊕⊕⊕<br>High	**HR 0.79**<br>(0.63 to 0.98)
**P+C compared to M**			
PFS (HR)	1152<br>(0 RCTs)	⊕◯◯◯<br>Very low	**HR 0.54**<br>(0.34 to 0.86)
OS (HR)	1187<br>(0 RCTs)	⊕◯◯◯<br>Very low	**HR 0.52**<br>(0.38 to 0.70)
**P+C compared to M+K**			
PFS (HR)	557<br>(0 RCTs)	⊕◯◯◯<br>Very low	**HR 0.51**<br>(0.30 to 0.87)
OS (HR)	592<br>(0 RCTs)	⊕◯◯◯<br>Very low	**HR 0.48**<br>(0.34 to 0.70)
**P+C compared to K**			
PFS (HR)	2063<br>(1 RCT)	⊕⊕⊕⊕<br>High	**HR 0.38**<br>(0.25 to 0.56)
OS (HR)	2489<br>(1 RCT)	⊕⊕⊕◯<br>Moderate	**HR 0.51**<br>(0.40 to 0.66)
**P compared to M**			
PFS (HR)	2472<br>(0 RCTs)	⊕◯◯◯<br>Very low	**HR 0.77**<br>(0.54 to 1.11)
OS (HR)	2497<br>(0 RCTs)	⊕◯◯◯<br>Very low	**HR 0.66**<br>(0.51 to 0.84)
**P compared to C+K**			
PFS (HR)	2005<br>(0 RCTs)	⊕◯◯◯<br>Very low	**HR 0.74**<br>(0.48 to 1.14)
OS (HR)	2091<br>(0 RCTs)	⊕◯◯◯<br>Very low	**HR 0.86**<br>(0.66 to 1.12)
**P compared to K**			
PFS (HR)	3383<br>(3 RCTs)	⊕⊕◯◯<br>Low	**HR 0.54**<br>(0.41 to 0.70)
OS (HR)	3799<br>(3 RCTs)	⊕⊕◯◯<br>Low	**HR 0.65**<br>(0.55 to 0.76)
**M+P compared to M**			
PFS (HR)	939<br>(0 RCTs)	⊕◯◯◯<br>Very low	**HR 0.89**<br>(0.49 to 1.62)
OS (HR)	939<br>(0 RCTs)	⊕◯◯◯<br>Very low	**HR 0.69**<br>(0.41 to 1.17)
**M+P compared to C**			
PFS (HR)	861<br>(0 RCTs)	⊕◯◯◯<br>Very low	**HR 0.64**<br>(0.36 to 1.13)
OS (HR)	1226<br>(0 RCTs)	⊕◯◯◯<br>Very low	**HR 0.75**<br>(0.46 to 1.21)
**C+K compared to C**			
PFS (HR)	889<br>(0 RCTs)	⊕◯◯◯<br>Very low	**HR 0.76**<br>(0.46 to 1.23)
OS (HR)	1315<br>(1 RCT)	⊕⊕◯◯<br>Low	**HR 0.82**<br>(0.63 to 1.06)
**C+K compared to K**			
PFS (HR)	1878<br>(2 RCTs)	⊕◯◯◯<br>Very low	**HR 0.73**<br>(0.50 to 1.05)
OS (HR)	2330<br>(2 RCTs)	⊕◯◯◯<br>Very low	**HR 0.76**<br>(0.61 to 0.94)
**M compared to M+K**			
PFS (HR)	839	⊕◯◯◯	**HR 0.95**

	(0 RCTs)	Very low	(0.60 to 1.65)
OS (HR)	433	⊕○○○	**HR 0.72**
	(0 RCTs)	Very low	(0.51 to 1.01)
**M compared to C**			
PFS (HR)	1356	⊕○○○	**HR 0.72**
	(0 RCTs)	Very low	(0.46 to 1.13)
**C compared to M**			
OS (HR)	1721	⊕○○○	**HR 0.93**
	(0 RCTs)	Very low	(0.72 to 1.21)
**C compared to K**			
PFS (HR)	2267	⊕○○○	**HR 0.96**
	(0 RCTs)	Very low	(0.66 to 1.40)
OS (HR)	3023	⊕⊕○○	**HR 0.92**
	(1 RCT)	Low	(0.76 to 1.11)
**M+K compared to K**			
PFS (HR)	1750	⊕⊕⊕○	**HR 0.73**
	(3 RCTs)	Moderate	(0.52 to 1.04)
	(0 RCTs)	Very low	(0.62 to 1.45)
OS (HR)	839	⊕○○○	**HR 0.94**
	(0 RCTs)	Very low	(0.68 to 1.30)
**M compared to K**			
PFS (HR)	2345	⊕⊕○○	**HR 0.70**
	(4 RCTs)	Low	(0.54 to 0.89)
OS (HR)	2736	⊕⊕⊕○	**HR 0.99**
	(4 RCTs)	Moderate	(0.83 to 1.19)
**C compared to M+K**			
OS (HR)	1126	⊕○○○	**HR 0.87**
	(0 RCTs)	Very low	(0.63 to 1.21)
**M+K compared to C**			
PFS (HR)	761	⊕○○○	**HR 0.76**
	(0 RCTs)	Very low	(0.45 to 1.27)
**K compared to M+K**			
OS (HR)	2141	⊕⊕⊕○	**HR 0.95**
	(3 RCTs)	Moderate	(0.73 to 1.25)

GRADE Working Group grades of evidence
High certainty: we are very confident that the true effect lies clOS (HR)e to that of the estimate of the effect.
Low certainty: our confidence in the effect estimate is limited: the true effect may be substantially different from the estimate of the effect.
Very low certainty: we have very little confidence in the effect estimate: the true effect is likely to be substantially different from the estimate of effect.
CI: confidence interval; HR: hazard ratio; B: BRAF inhibitor; M: MEK inhibitor; P: PD-(L)1 inhibitor; C: CTLA-4 inhibitor; K: chemotherapy; PFS: progression-free survival; OS: overall survival; RCT: randomized controlled trial
